# One-Step Synthesis of Chiral Oxindole-type Analogues with Potent Anti-inflammatory and Analgesic Activities

**DOI:** 10.1038/srep13699

**Published:** 2015-09-01

**Authors:** Yulong Sun, Jia Liu, Xianxing Jiang, Tao Sun, Luping Liu, Xiaoyuan Zhang, Shaoli Ding, Jingyi Li, Yan Zhuang, Yiqing Wang, Rui Wang

**Affiliations:** 1 School of Pharmaceutical Sciences, Sun Yat-Sen University, Guangzhou, China, 510006; 2 Shanghai Institute for Advanced Immunochemical Studies, ShanghaiTech University, Shanghai, China, 200031; 3 Key Laboratory of Preclinical Study for New Drugs of Gansu Province, School of Basic Medical Sciences, Lanzhou University, China, 730000; 4 Anesthesia department of Gansu Provincial Hospital, China, 730000; 5 Key Laboratory for Reproductive Medicine and Embryo, The Reproductive Medicine Research Center of the First Hospital of Lanzhou University, Gansu, China, 730000

## Abstract

Here we report a facile approach to synthesize highly optically active oxindole-type analogues with both high yield and enantioselectivity. This single-step synthesis strategy represents a substantial improvement upon existing methods that are often involved with multi-step routes and have suboptimal atomic economy. One such compound, namely Q4c, showed remarkable *in vivo* anti-inflammatory activity with efficiency approaching to that of a steroidal compound dexamethasone. Moreover, Q4c alleviated pain in mouse models with comparable activity to morphine. Further investigation suggested that nitric oxide signaling pathway is involved in the anti-inflammatory and analgesic activities of Q4c. Notably, this is the first time that chiral oxindole-type analogues have been identified to be both anti-inflammatory and analgesic, and our study also paved the way for future development of oxindoles as drug candidates in this field.

Nonsteroidal anti-inflammatory drugs (NSAIDs) are among the most commonly prescribed medications for reducing pain and inflammation. While NSAIDs exert their pharmacological activity by inhibiting cyclooxygenases (COXs)^1^, they represent a wide range of compounds with chemically distinct structures. Despite their notable anti-inflammatory activity, NSAIDs are usually associated with gastrointestinal, cardiovascular and renal complications^2^,^3^,^4^. Further improvements on anti-inflammatory medications require more detailed understanding of inflammatory biology and perhaps development of novel anti-inflammatory drugs with high efficacy yet low side effects.

Traditional NSAIDs usually require multi-step synthesis procedure. For example, ibuprofen, one of the most widely used NSAIDs on the market, is produced through a three-step synthesis route^5^. The complexity of synthesis reaction has been a major obstacle to reducing the cost of anti-inflammatory agents. Rapid, efficient synthesis methodologies play a critical role in current medicinal chemistry. Construction of chiral compounds on the basis of biologically relevant frameworks has been the focus of medicinal chemists for decades. This target-oriented organic synthesis has greatly advanced the overall speed of drug discovery^6^,^7^. To date, however, it remains a great challenge to develop a highly efficient and simply-convenient strategy, thereby contributing to the development of new therapeutic agents.

Chiral oxindole-type molecules are increasingly important lead compounds for a wide range of biologically active molecules^8^,^9^,^10^,^11^ Evaluation of optically active oxindoles as therapeutic agents requires the development of a facile, robust synthesis route. On the basis of our recent efforts to develop novel methods to synthesize biologically and pharmaceutically active chiral spirooxindole-type alkaloids by asymmetric strategies in our group (Fig. 1A,B)^12^,^13^. Interestingly, one such compound, JP-8 g, exhibited not only a wide range of anti-cancer activity^13^, but also potent *in vivo* anti-inflammatory activity^14^. *In vitro* and *in vivo* experiments suggested that JP-8g exerted anti-inflammatory activity through nitric oxide (NO) signaling pathway^14^. Most interestingly, when compared with a traditional NSAID, indomethacin, JP-8 g showed dramatically reduced acute gastric damage^15^. These results suggested that spirooxindoles as anti-inflammaory agents may possess some advantages over conventional NSAIDs.

In order to expedite the evaluation of their therapeutic potential, we sought to establish a simple and robust approach for the synthesis of oxindole derivatives. Although our previous methods are highly efficient, SPXs^12^ and JPs^13^ series compounds still require multi-step synthesis. In this study, we first designed a single-step approach for the asymmetric synthesis of spirooxindole-type compounds and explored the feasibility of using these compounds as anti-inflammatory and analgesic drug candidates. This single-step synthesis strategy represents a substantial improvement upon existing methods that are often involved with multi-step routes and have suboptimal atomic economy.

## Results

### Synthesis of chiral oxindole-type compounds

**Figure 1:**
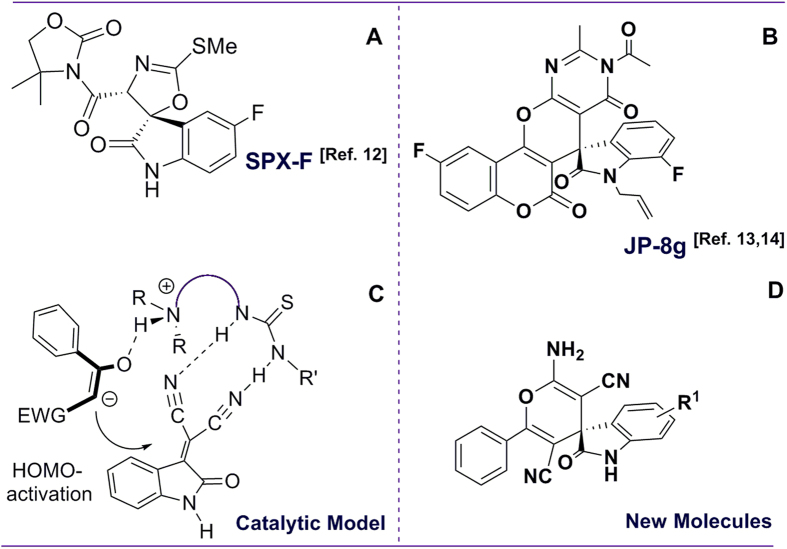
Synthesis of optically and biologically active spirooxindole-type alkaloids by asymmetric strategies. (**A**) SPX-F with antipyretic activity, three-step synthesis^12^; (**B**) JP-8 g with inflammatory and anti-cancer activities, three-step synthesis^13^,^14^; (**C**) proposed catalytic mechanism for the one-step synthesis of oxindoles in this study; (**D**) the structure of new oxindole-type molecules.

Inspired by the bifunctional catalytic system-based asymmetric synthesis^16^,^17^,^18^, we rationalized that isatylidene malononitrile can be activated by two thiourea hydrogen atoms through weak hydrogen bonds. This can be achieved by the nucleophilic attack of enolate, which is promoted by the HOMO energy raised from the interaction between the tertiary amine moiety of catalyst and the carbonyl group of benzoylacetonitrile. As a result, this process leads to the formation of spirooxindole-type product (Fig. 1C,D), (Supplementary Fig. 1).

**Figure 2:**
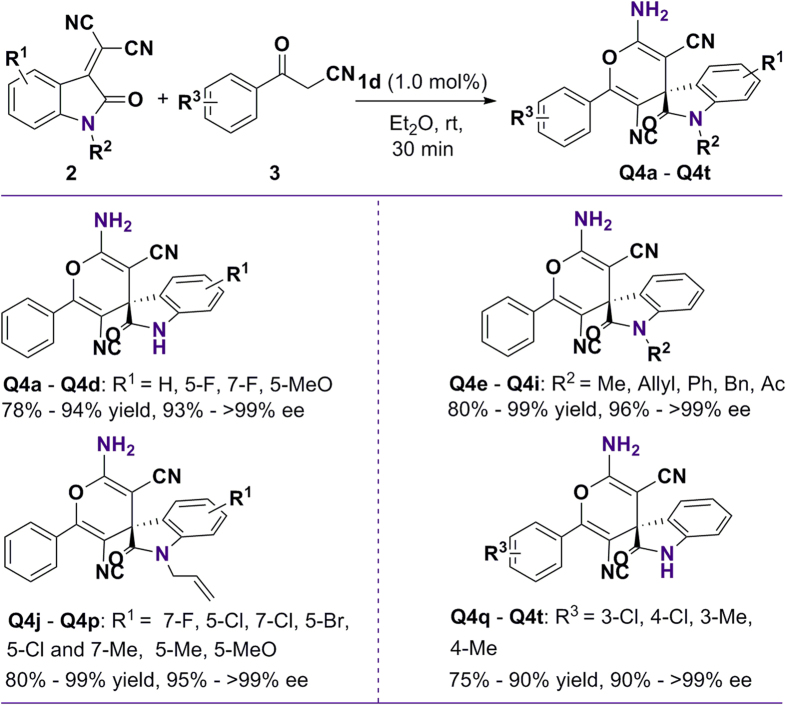
Synthesis of various isatylidene malononitriles under optimized conditions. Unless noted, the reactions were conducted with 2 (0.22 mmol) and 3 (0.20 mmol) using 1.0 mol% catalyst in Et_2_O for 30 min at room temperature. Yield and ee values were determined by HPLC, and configuration was assigned by comparison of retention time and specific rotation of obtained compounds with the data reported in previous literatures^13^,^14^. See Supplementary Fig. 3 for more details.

To explore the possibility of the proposed cyclization process, our investigation began with screening of reaction conditions to evaluate their catalytic activities (Supplementary Fig. 2). Subsequently, the results of experiments in which a variety of diversely structured spirooxindole-type compounds were ingeniously constructed under the optimized conditions are summarized in Fig. 2. In general, variation of the electronic properties of the substituent at different sites of the *N*-protected or unprotected isatylidenemalononitriles with different steric parameters was tolerated, giving the corresponding spiro[indoline-3,4′-pyran]s in excellent enantioselectivities (93–>99% ee, **Q4a** – **Q4p**) and good to high yields. As expected, various substituted benzoylacetonitriles at different positions on the aromatic ring could also be tolerated, and gave the corresponding compounds **Q4q** – **Q4t** with excellent enantioselectivities (90–>99% ee) in yields ranging from 75 to 90%. Our synthetic method shows higher atom economy without using any metal, as well as operationally simple process with low costs will benefit for using of these potential compounds in pharmaceuticals discovery.

### Structure and activity relationship (SAR) of Q series compounds

**Figure 3:**
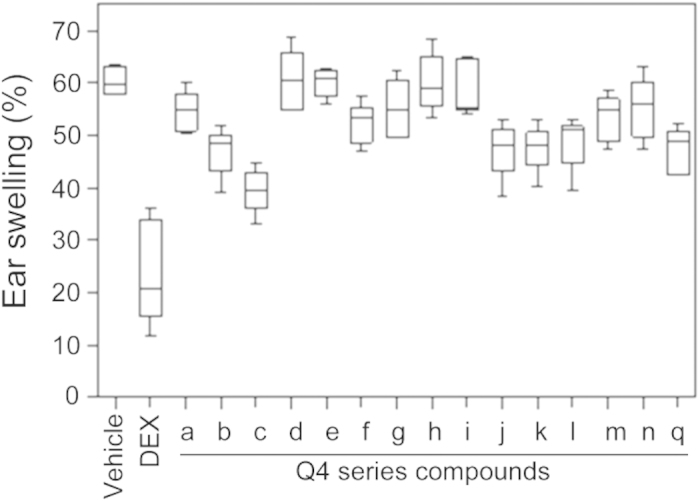
Structure-activity relationship of Q series compounds. Synthesized spirooxindole compounds (12.5 mg/kg) were tested using mouse ear inflammation model (*n *= 6). A steroidal anti-inflammatory drug dexamethasone (DEX, 5.0 mg/kg) was used as a reference compound.

Despite structurally different from traditional NSAIDs, chiral spirooxindole framework has been discovered to exhibit antipyretic, anti-cancer and anti-inflammatory activities^12^,^13^,^14^. After constructing a series of compounds with optically active spiro [indoline-3,4′-pyran] frameworks, we sought to evaluate their therapeutic potential as anti-inflammatory and analgesic agents. We first studied the structure-activity relationship (SAR) of selected compounds using a mouse ear inflammation model. As shown in Fig. 3, many of these compounds showed significant suppression of ear swelling. Excitingly, one compound, Q4c, exhibited remarkable *in vivo* anti-inflammatory activity.

### Anti-inflammatory and analgesic activity of Q4c

**Figure 4:**
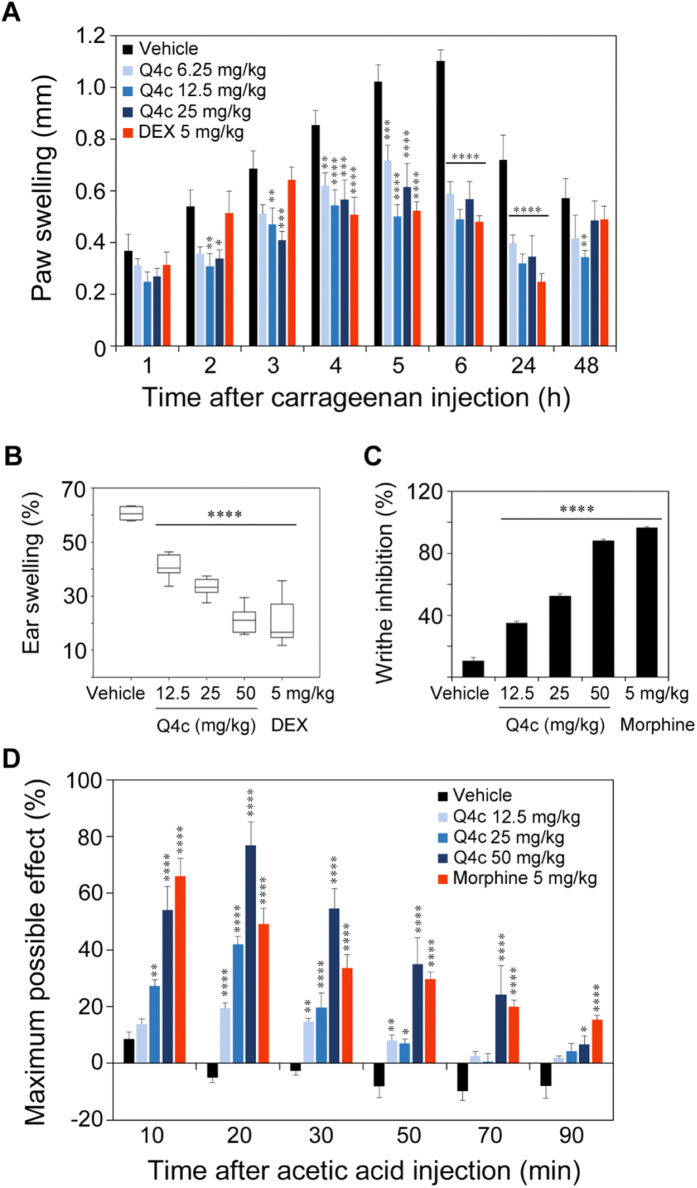
The *in vivo* anti-inflammatory and analgesic activity of Q4c. (**A**) Carrageenan-induced paw inflammation model (*n *= 7). (**B**) Xylene-induced ear inflammation model (*n *= 10). (**C**) Tail flick pain model (*n *= 6). (**D**) Acetic acid twist body pain model (*n *= 6). Data are expressed as mean ± SEM. Statistical evaluation was performed by two-way ANOVA, followed by Tukey post-tests (*p < 0.05; **p < 0.01; ***p < 0.005; ****p < 0.001).

We next evaluated the time- and dose-dependency of the anti-inflammatory activity of Q4c using mouse paw inflammation model. Swelling of paws was efficiently induced by carrageenan injection during the entire 48 h period and peaked at 4–6 h (Fig. 4A, vehicle). A reference drug dexamethasone (DEX) showed significant suppression (p < 0.001, Tukey’s post-tests) of paw swelling at 4–24 h after carrageenan injection. Q4c of doses ranging from 6.25 to 25.0 mg/kg inhibited the progression of inflammation with efficacy comparable to that of DEX. Similarly, Q4c showed potent anti-inflammatory activity on mouse ear inflammation with the highest dose (50.0 mg/kg) as potent as 5.0 mg/kg DEX (Fig. 4B).

To further understand the therapeutic potential of Q4c, we sought to test whether it had analgesic activity. As seen in Fig. 4C, Q4c effectively suppressed radiant heat-stimulated tail flick. Q4c of high dose (50.0 mg/kg) exhibited efficacy approaching to that of morphine (5.0 mg/kg). In addition, we studied the time- and dose-response of Q4c activity on mouse acetic acid-induced twist model. Mice were pre-treated with vehicle and test compounds for 15 min before the administration of acetic acid. We found that Q4c showed potent *in vivo* analgesic activity toward the stimulation of acetic acid. It is also worth noting that this activity was largely dose dependent. Consistent with the results on tail flick pain model, high dose of Q4c (50.0 mg/kg) attenuated acetic acid-induced body twist with efficacy comparable to that of morphine (5.0 mg/kg) (Fig. 4D).

### *In vitro* and *in vivo* toxicity of Q4c

To evaluate the potential of Q4c as a safe medication, we first analyzed its effect on the viability of isolated mouse primary peritoneal macrophages. Minor cytotoxicity was observed only at the highest concentration (50 μM), while Q4c at or below 40 μM had no significant effect on cell viability (Supplementary Fig. 4). We next evaluated the *in vivo* toxicity of Q4c on Wistar rat using acute gastric damage model. In comparison with indomethacin, Q4c exhibited significantly reduced gastric damage (Supplementary Fig. 5). Nevertheless, it is worth noting that Q4c may not bear the same *in vivo* target as indomethacin does. Indomethacin is a nonselective inhibitor of COX-1 and COX-2 whereas the target of Q4c is unknown. *In vitro* inhibition experiments suggested that the median inhibition concentrations (IC_50_) of Q4c on COX-1 and COX-2 are both above 50 μM, indicating that Q4c is not a potent COX inhibitor (Supplementary Fig. 6). We speculate that Q4c may either require *in vivo* metabolic processing for COX targeting, or bear a completely novel target for its *in vivo* activity.

### Mechanism of actions of Q4c

**Figure 5:**
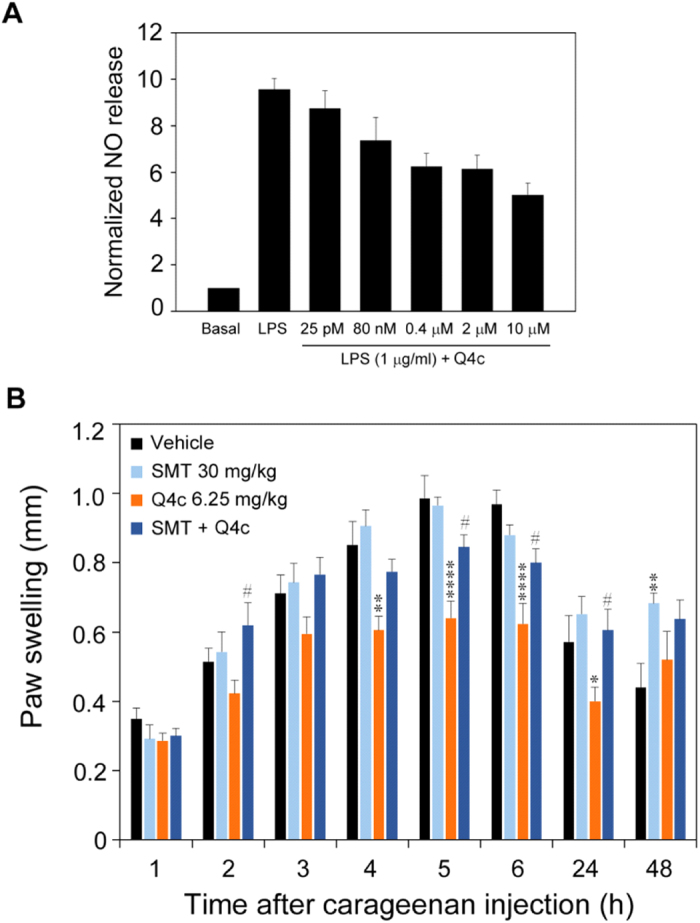
Mechanism of actions of Q4c. (**A**) The effect of Q4c on LPS-stimulated NO release from primary peritoneal macrophages. Nitrite levels were assessed by Griess reagent. Data are shown as mean ± SEM (*n *= 5). Significant difference between LPS and test groups are indicated, **p < 0.01; ***p < 0.001.(**B**) The effect of iNOS antagonist SMT on the *in vivo* anti-inflammatory activity of Q4c. Data are shown as mean ± SEM (*n *= 7). Significant difference from vehicle, *p < 0.05; **p < 0.01; ***p < 0.005; ****p < 0.001. Significant difference between Q4c treatments in the absence and presence of SMT, #p < 0.05. Statistical analysis was performed by two-way ANOVA, followed by Tukey’s post-tests.

To explore how Q4c exerts its anti-inflammatory and analgesic activities, we next examined the signaling pathways that may be involved in its function. Our previous study suggested that nitric oxide synthase (NOS) signaling pathway was involved in the anti-inflammatory activity of JP-8 g, another spirooxindole compound^14^. Therefore, we sought to determine the effect of Q4c on LPS-stimulated nitric oxide (NO) release in isolated primary macrophages. As seen in Fig. 5A, Q4c suppressed NO release in a dose-dependent manner. Q4c of 10 μM achieved more than 50% suppression. To confirm the role of NOS signaling pathway, we evaluated the effect of NOS inhibitors on the anti-inflammatory activity of Q4c using mouse paw swelling model. It was observed that 30 mg/kg of inducible NOS (iNOS) inhibitor SMT significantly (p < 0.05) reduced the anti-inflammatory activity of Q4c 5–24 h after carrageenan injection (Fig. 5B). In contrast, NF-kB inhibitor BAY 11–7082 did not appear to affect the *in vivo* activity of Q4c (Supplementary Fig. 7). This is consistent with our previous findings that NOS pathway, but not NF-κB pathway, is involved in the anti-inflammatory activity of spirooxindole compounds^15^.

## Discussion

Here we described a facile method to synthesize chiral oxindole analogues in a single step and explored the feasibility of using these compounds as anti-inflammatory and analgesic drug candidates. Due to its simplicity, atomic economy, metal-free feature, and high yield and enantioselectivity, this reaction could provide very valuable means for preparing biologically active spirooxindoles. Importantly, one product from this reaction, Q4c, exhibited potent *in vivo* anti-inflammatory and analgesic activity. Q4c is different from traditional NSAIDs in their chemical structure, and *in vitro* inhibition experiments suggested that Q4c could not efficiently inhibit COX enzymes. Further studies indicated that Q4c exerts the anti-inflammatory activity through NOS signaling pathway, though its *in vivo* target and detailed mechanism of actions are yet to be elucidated. Our data also showed that Q4c had only minor *in vitro* cytotoxicity to primary peritoneal macrophage and caused no appreciable acute gastric damage. It is important, however, to carefully assess the safety of Q4c using other animal models before its therapeutic potential can be considered.

## Methods

Synthesis of Q series compounds, and evaluation of *in vivo* anti-inflammatory and analgesic activities of Q4c. See Supplementary Information for more details.

### Ethics Statement

All animal experiments were carried out in accordance with the approved guidelines of China Council on Animal Care and Use. All animal procedures performed in this study were approved by the Institutional Animal Care and Use Committee of the Ethics Committee of Lanzhou University, China.

### Animal Experiments

All animals (C57BL/6J mice and Wistar rats) were maintained and the experiments were carried out in accordance with the European Community guidelines for the use of experimental animals (86/609/EEC). All the protocols in this study were executed under the guideline of the Ethics Committee of Lanzhou University, China. Find Supplementary Information for further details.

### Statistical Analysis

Statistical significance of results was analyzed by one- or two-way ANOVA using Prism software version 6.01 (GraphPad Software, San Diego, CA, USA) followed by Tukey’s post-tests, respectively. Data were presented as mean ± SEM unless noted otherwise.

## Additional Information

**How to cite this article**: Sun, Y. *et al.* One-Step Synthesis of Chiral Oxindole-type Analogues with Potent Anti-inflammatory and Analgesic Activities. *Sci. Rep.* **5**, 13699; doi: 10.1038/srep13699 (2015).

## Supplementary Material

Supplementary Information
