# Supplementary material for: One-Step Synthesis of Chiral Oxindole-type Analogues with Potent Anti-inflammatory and Analgesic Activities
Source: Sci Rep. 2015 Sep 1;5:13699. doi: 10.1038/srep13699 (PMC4555174; doi:10.1038/srep13699)
Supplement: Supplementary Information [file srep13699-s1.pdf]

Supplementary Information

One-Step Synthesis of Chiral Oxindole-type Analogues with Potent Anti-inflammatory and Analgesic Activities

Yulong Sun[1,3†], Jia Liu[2†], Xianxing Jiang[1†]*, Tao Sun[3], Luping Liu[3], Xiaoyuan Zhang[3], Shaoli Ding[4], Jingyi Li[3], Yan Zhuang[3], Yiqing Wang[5] & Rui Wang[1,3]*

[1] School of Pharmaceutical Sciences, Sun Yat-Sen University, Guangzhou, China, 510006.

[2] Shanghai Institute for Advanced Immunochemical Studies, Shanghaitech University, Shanghai, China, 200031.

[3] Key Laboratory of Preclinical Study for New Drugs of Gansu Province, School of Basic Medical Sciences, Lanzhou University, China, 730000.

[4] Anesthesia department of Gansu Provincial Hospital, China, 730000.

[5]Key Laboratory for Reproductive Medicine and Embryo, The Reproductive Medicine Research Center of the First Hospital of Lanzhou University, Gansu, China, 730000

[†]Co-first author (these authors contributed equally to this work)

Table of contents

1. **Supplementary Materials and Methods (S3-S5)**
2. **Figures (S6-S12)**
3. **Supplementary References (S13-S14)**
4. **Characterization of Synthesized Compounds (S15-S82)**

Supplementary Materials and Methods

1. *General methods for synthesis of spirooxindole compounds.*

All solvents were dried according to established procedures. Reactions were monitored by thin layer chromatography (TLC) and column chromatography purifications were carried out using silica gel GF254. Proton and carbon nuclear magnetic resonance (^{1}H NMR and ^{13}C NMR) spectra were recorded on Brucker 300 MHz spectrometer in $CDCl_3$ or [D^6]DMSO using tetramethylsilane (TMS) as internal standard unless otherwise noted. Data are presented as follows: chemical shift, integration, multiplicity (br = broad, s = singlet, d = doublet, t = triplet, q = quartet, m = multiplet) and coupling constant in Hertz (Hz). Infrared (IR) spectra were recorded on a FT-IR spectrometer. Optical rotations were recorded on a Perkin-Elmer 341 polarimeter. HR-MS was measured with an APEX II 47e mass spectrometer. The ee value determination was carried out using chiral high-performance liquid chromatography (HPLC) with Daicel Chiracel AD-H column on Waters with a 2998 UV-detector.

2. *Ethics Statement*

All animal experiments were performed in accordance with the guidelines of China Council on Animal Care and Use. All animal procedures carried out in this study were reviewed, approved, and supervised by the Institutional Animal Care and Use Committee of the Ethics Committee of Lanzhou University, China.

3. *Isolation of primary peritoneal macrophages*

This method has been used elsewhere [1]. Briefly, primary mouse peritoneal macrophages were obtained from C57BL/6 male mice (25-31 g) by intraperitoneal (i.p.) injection of 2 mL of sterile 3% Brewer thioglycollate broth. Four days later, cells were harvested by a lavage of the peritoneal cavity with 5 ml of RPMI 1640 medium containing 10% fetal bovine serum (FBS, Life Technologies). The primary peritoneal macrophages were seeded on to 96-well plates, kept for 2 h on plates to adhere and then washed three times with pre-warmed medium to remove the non-adherent cells. Macrophages were allowed to rest for 24 h in fresh complete medium before treatment with different drugs.

4. *Cytotoxic activity assays*

Cytotoxicity of compounds was assessed by standard MTT assay. Mouse primary peritoneal macrophage cells (5×10^3/well in 90 µL medium) were seeded in 96-well plates and cultured for 24 h. Compounds of different concentrations ranging from 10 – 50 µM were added to the corresponding wells, and the plates were incubated for 72 h. Then, 10 µL of MTT (Sigma-Aldrich, St. Louis, MO, USA) solution (5.0 mg/mL) was added to each well. After 4 h of incubation at 37 ºC, the medium was aspirated and the dye crystals were removed by DMSO wash. The absorbance at 490 nm of each sample was then determined by ELISA plate reader (Model 680, BioRad, Hercules, CA, USA).

5. *Nitrite assay for estimation of NO production*

The nitrite assay used were described previously [2]. Primary peritoneal macrophages (1×10^4/well) were plated in 96-well plates in MEM with 10% heat-inactivated FBS, and then treated with test compounds for 72 h. The supernatant was removed and fresh media containing 1 µg/mL lipopolysaccharide (LPS, *Escherichia coli* serotype 0111:B4, Life Technologies). The culture was incubated for 72 h and the accumulation of stable nitrite in culture supernatants was evaluated by Griess reaction [3]. Briefly, 100 µL medium supernatant from each well was transferred into an empty 96-well plate and then 100 µL Griess reagent (Sigma) was added into each well. The reaction was kept at room temperature for 15 min and the absorbance at 540 nm was determined using Tecan infinite M200 (Tecan Group, Männedorf, Switzerland). NO production was calculated according to a standard curve of sodium nitrite of known concentrations.

6. *Acute gastric damage assay*

Wistar rats (180-200 g) were placed in the operation room 72 h before the experiments for acclimatization. At 12 h before the test, rats were placed in cages with rinsed mesh bottoms with free access to water, but no food. Animals were assigned randomly to three groups with five animals per group. Indomethacin (30 mg/kg), Q4c (50 mg/kg) or vehicle only was orally administrated to rats in a volume/weight ratio of 5 ml/kg. All compounds were freshly prepared before dosing. At 6 h post dosing, rats were sacrificed using carbon dioxide. The stomach was removed from rats, opened along the greater curvature, gently rinsed with water, placed on petri dishes, and then digitally photographed by SONY W80 Cyber-shot camera (SONY, Japan).

7. *Eevaluation of in vivo anti-inflammatory and analgesic activities of Q4c*

Prior to experiments, animals (C57BL/6 mice and Wistar rats)were housed for 1 week at stable conditions with temperature of 20-23 °C and humidity of 65-75%. Settled artificial light cycle and access to food and water *ad libitum* were provided. All animals were kept and experiments were performed in accordance with the European Community guidelines for the use of experimental animals (86/609/EEC). All the protocols in this study were executed under the guideline of the Ethics Committee of Lanzhou University, China. Animals received human care and all efforts were undertaken to minimize animal suffering before and during experiments.

Xylene-induced ear edema model. Topical application of xylene can cause acute inflammation which is characterized by edema and granulocytic cell infiltration into the skin. Experiments were executed as described previously[4]. Briefly, mice were i.p. treated with vehicle, Q4c of varying concentrations and a traditional NSAID indomethacin as a reference compound. At 30 min after drug administration, 10.0 µL of 2.5% (v/v) xylene dissolved in acetone solution was applied to the anterior and posterior surface of the right ear. The left ear remained untreated. Control animals received only the irritant, while the test mice received both the irritant and drugs. At the time of maximum inflammatory response (~3 h after xylene treatment), mice were sacrificed by cervical dislocation and a tissue disk of 6 mm diameter was cut from each ear lobe. Edema was calculated by the difference in weight between the samples from right treated and left untreated ears. The anti-edematous activity of compounds was presented as percent inhibition of ear swelling in mice treated with the test substances with respect to that in control

mice treated with the irritant alone[5, 6, 7, 8]. A total of ten mice were employed for each group of treatment.

Carrageenan-induced paw inflammation model. Acute inflammation was induced by intraplantar (i.pl.) injection of 25.0 μL of carrageenan (1% w/v in saline) into the right hind paw of male mice[9]. The experimental setup constituted five groups with seven mice per group. Group 1 received an i.p. treatment of 60.0 μL vehicle saline solution containing 5% (v/v) DMSO and 5% (v/v) Cremophor EL. At 30 min after vehicle administration, 25.0 μL of 1%(w/v) carrageenan (Life Technologies) in saline solution was s.c. injected in the right footpad and 25.0 μL of saline in the left footpad. Group 2-4 were injected i.p. with 300.0μL of Q4c for varying doses followed by carrageenan treatment. Group 5 was treated i.p. with 300.0 μL of DEX (5.0 mg/kg, a reference drug) followed by carrageenan injections. As for the groups to test the effects of various inhibitors, mice were administrated with Q4c (25.0 mg/kg, i.p.) in the presence or absence of different inhibitors. To examine the effects of inhibitors, mice were i.pl. administrated with 25.0 mg/kg of Q4c in the presence or absence of different inhibitors. NF-κB pathway inhibitor BAY 11-7082 and iNOS inhibitor *S*-methyl-ITU (SMT) were examined in this study. The thickness of left and right footpads was measured using a caliper at 1, 2, 3, 4, 5, 6, 24 and 48 h post dosing. The difference in the thickness of left untreated and right treated footpads was calculated. This study was executed in a blinded manner.

Acetic acid writhingtest. The writhing test was carried out according to the method of previous study[10, 11]. Male mice were treated with Q4c (12.5, 25.0, 50.0 mg/kg, i.p.) or morphine (5.0 mg/kg, i.p.)for 15 min and then 0.6% freshly prepared aqueous solution of acetic acidwas i.p. administrated. The number of writhes was recorded at 15min after injection of acetic acid. The analgesic activity of compounds was presented as the percentage decrease in the number of writhes and calculatedusing the following formula: % inhibition of writhes = $(V - D)/V \times 100$, where V and D present the number of writhes in vehicle-treated group or drug-treated group, respectively.

Tail-flick test. The nociceptive response was assessed by the radiant heat tail-flick test. Male mice weighing 20.0-22.0 g were used. The animals were gently restrained by hand, and a light beam was focused onto the tail. At the beginning of the study, the lamp intensity was adjusted to elicit a response in control animals within 3-5 s. A cut-off time was set at 10 s to minimize tissue damage. Tail-flick time was determined before injection and 5, 10, 15, 20, 30, 40, 50 and 60 min after injection. Every male mouse was used only once.

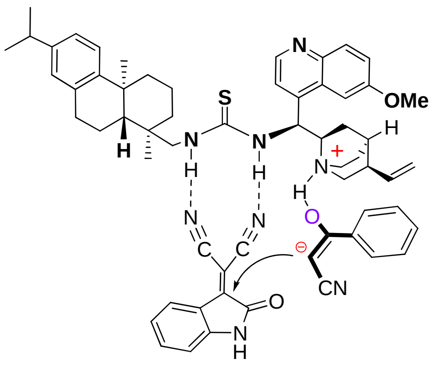

Supplementary Figure 1. Proposed mechanism of the asymmetric synthesis of novel spirooxindole-type pyranopyrimidines.

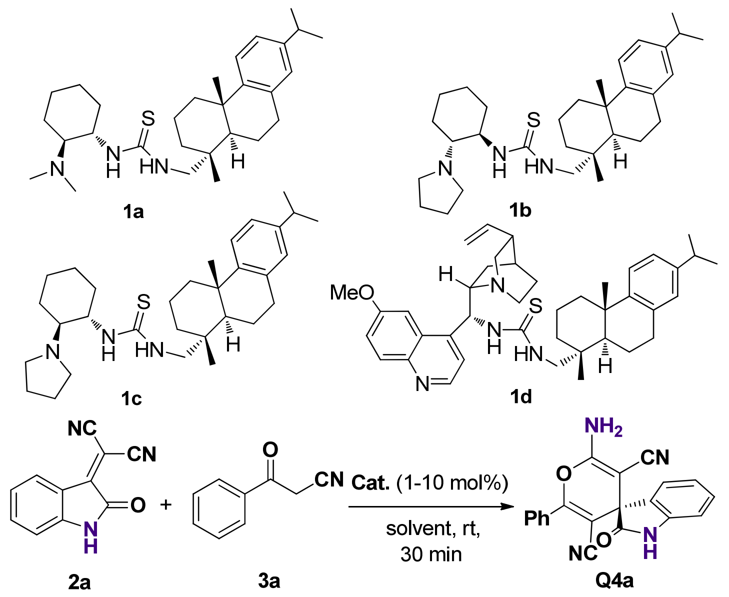

Entry	Cat.	Solvent	Yield [%]	ee [%]
1	**1a**	CH_2Cl_2	91	65
2	**1b**	CH_2Cl_2	92	85
3	**1c**	CH_2Cl_2	88	-79
4	**1d**	CH_2Cl_2	90	98
5	**1d**	toluene	80	55
6	**1d**	THF	40	36
7	**1d**	Et_2O	94	99
8	**1d**	MeOH	50	10

Supplementary Figure 2. Optimization of reaction parameters. Unless noted, the reaction was conducted with **2a** (0.22 mmol) and **3a** (0.20 mmol) using 1.0 mol% catalyst for 30 min at room temperature. Yield and ee values were determined by HPLC, and configuration was assigned by comparison of retention time and specific rotation of the obtained compounds with data reported in literature. [12,13]

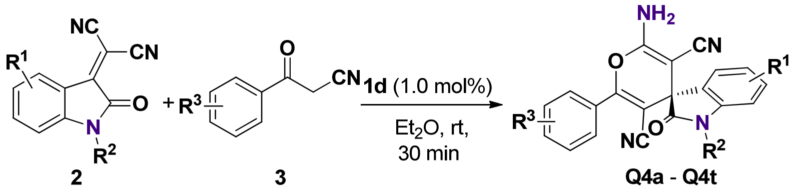

entry	R[1]	R[2]	R[3]	Yield[%] [b]	ee [%] [c]
1	H	H	H	94 (Q**4a**)	99
2	5-F	H	H	78 (Q**4b**)	>99
3	7-F	H	H	92 (Q**4c**)	93
4	5-MeO	H	H	84 (Q**4d**)	94
5	H	Me	H	80 (Q**4e**)	95
6	H	Ally	H	99 (Q**4f**)	95
7	H	Ph	H	87 (Q**4g**)	98
8	H	Bn	H	89 (Q**4h**)	96
9	H	Ac	H	85 (Q**4i**)	>99
10	7-F	Ally	H	93 (Q**4j**)	95
11	5-Cl	Ally	H	80 (Q**4k**)	>99
12	7-Cl	Ally	H	87 (Q**4l**)	97
13	5-Cl,7-Me	Ally	H	99 (Q**4m**)	99
14	5-Br	Ally	H	90 (Q**4n**)	>99
15	5-Me	Ally	H	92 (Q**4o**)	>99
16	5-MeO	Ally	H	99 (Q**4p**)	>99
17	H	H	3-Cl	75 (Q**4q**)	>99
18	H	H	4-Cl	78 (Q**4r**)	>99
19	H	H	3-Me	88 (Q**4s**)	>99
20	H	H	4-Me	90 (Q**4t**)	90

Supplementary Figure 3. Synthesis of various isatylidene malononitriles under optimized conditions. Unless noted, the reactions were conducted with **2** (0.22 mmol) and **3** (0.20 mmol) using 1.0 mol% catalyst in Et_2O for 30 min at room temperature. Yield and ee values were determined by HPLC, and configuration was assigned by comparison of retention time and specific rotation of the obtained compounds with data reported in literature.[12,13]

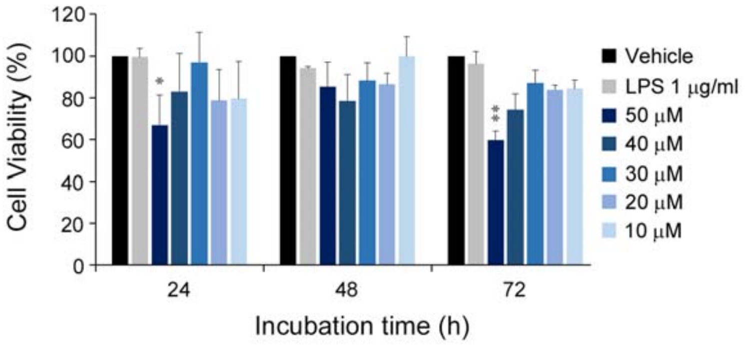

Supplementary Figure 4. Cytotoxicity of Q4c on mouse primary peritoneal macrophages. Q4c of various concentrations was incubated with primary peritoneal macrophages for 24, 48 and 72 h and cell viability was determined by MTT assay. Data are shown as mean ± s.e. (n = 3). Significant difference between vehicle and test groups was evaluated by two-way ANOVA, followed by Tukey post-tests. *, significantly different from vehicle, *, $p < 0.05$; **, $p < 0.01$.

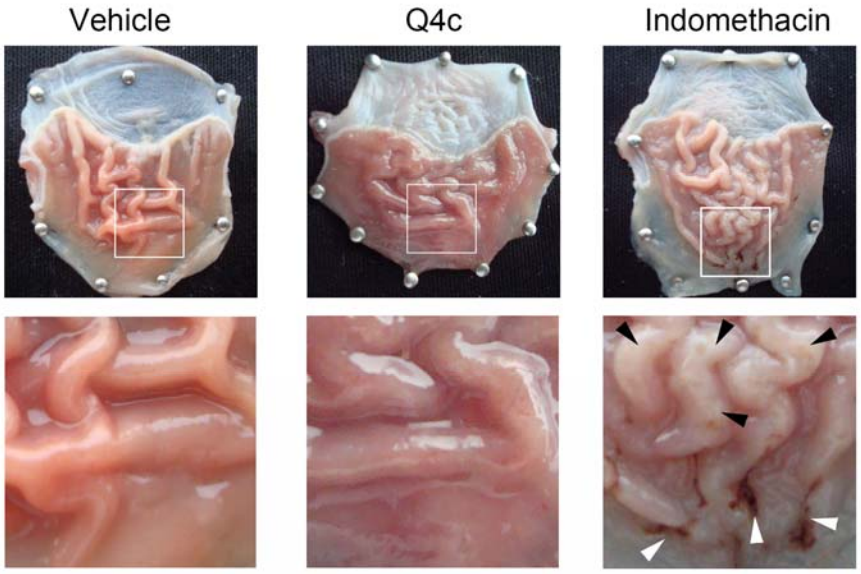

Supplementary Figure 5. Analysis of acute gastric damage of Q4c (30 mg/kg) and indomethacin (30 mg/kg). White and black arrows indicate hemorrhages and ulcers, respectively.

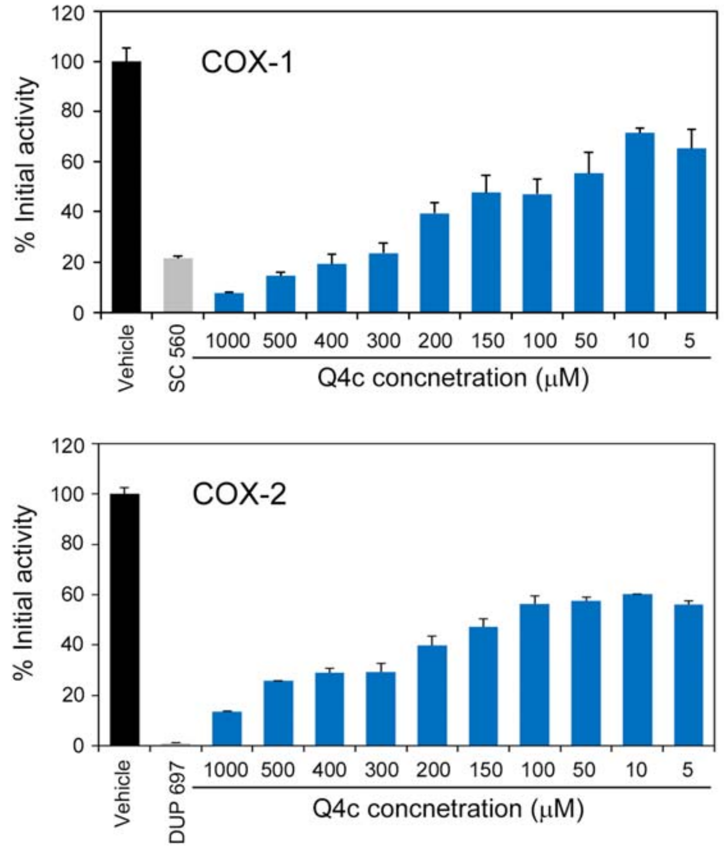

Supplementary Figure 6. *In vitro* inhibitory activity of Q4c to COX-1 and COX-2. The inhibition assay was performed using COX Fluorescent Inhibitor Screening Assay Kit (Cayman Chemical, Ann Arbor, MI, USA) following manufacturer's instructions. COX-1 inhibitor SC 560 (5 nM) and COX-2 inhibitor DUP 697 (25 nM) were used as controls[14,15].

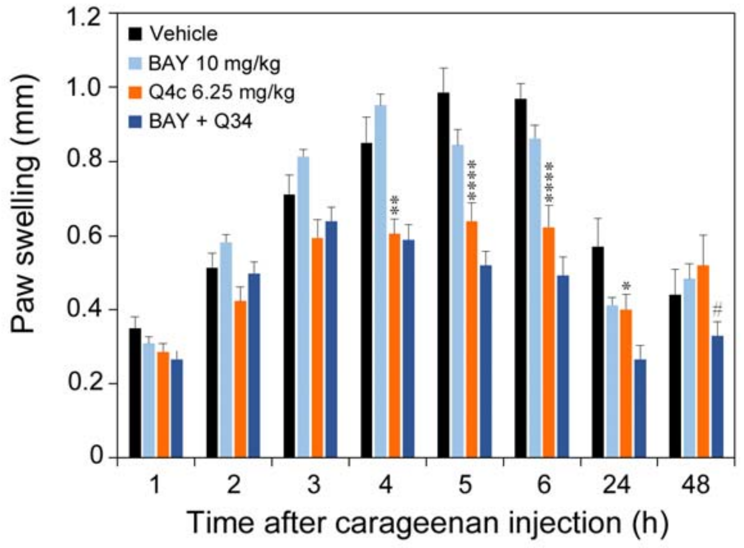

Supplementary Figure 7. The effect of NF-κB antagonist BAY 11-7082 on the *in vivo* anti-inflammatory activity of Q4c. The data of vehicle and Q4c in this figure were from the same experiments as represented in Fig. 5B. Data are shown as mean ± SEM (n = 7). Significant difference from vehicle, *, $p < 0.05$; **, $p < 0.01$; ***, $p < 0.005$; ****, $p < 0.001$. Significant difference between Q4c treatment in the absence and presence of BAY 11-7082, #, $p < 0.05$. Statistical analysis was performed by two-way ANOVA, followed by Tukey post-tests.

Supplementary References

1. Sun YL, Zhang XY, Sun T, He N, Li JY, Zhuang Y, *et al.* The anti-inflammatory potential of neuropeptide FF in vitro and in vivo. *Peptides* 2013, **47**(1)**:** 124-132.

2. Sun YL, Zhang XY, He N, Sun T, Zhuang Y, Fang Q, *et al.* Neuropeptide FF activates ERK and NF kappa B signal pathways in differentiated SH-SY5Y cells. *Peptides* 2012, **38**(1)**:** 110-117.

3. Ding AH, Nathan CF, Stuehr DJ. Release of reactive nitrogen intermediates and reactive oxygen intermediates from mouse peritoneal macrophages. Comparison of activating cytokines and evidence for independent production. *J Immunol* 1988, **141**(7)**:** 2407-2412.

4. Rotelli AE, Guardia T, Juarez AO, de la Rocha NE, Pelzer LE. Comparative study of flavonoids in experimental models of inflammation. *Pharmacol Res* 2003, **48**(6)**:** 601-606.

5. Schiantarelli P, Cadel S, Acerbi D, Pavesi L. Antiinflammatory activity and bioavailability of percutaneous piroxicam. *Arzneimittelforschung* 1982, **32**(3)**:** 230-235.

6. Tubaro A, Dri P, Delbello G, Zilli C, Della Loggia R. The croton oil ear test revisited. *Agents Actions* 1986, **17**(3-4)**:** 347-349.

7. Tubaro A, Dri P, Melato M, Mulas G, Bianchi P, Del Negro P, *et al.* In the croton oil ear test the effects of non steroidal antiinflammatory drug (NSAIDs) are dependent on the dose of the irritant. *Agents Actions* 1986, **19**(5-6)**:** 371-373.

8. Berg DJ, Leach MW, Kuhn R, Rajewsky K, Muller W, Davidson NJ, *et al.* Interleukin 10 but not interleukin 4 is a natural suppressant of cutaneous inflammatory responses. *J Exp Med* 1995, **182**(1)**:** 99-108.

9. Henriques MG, Silva PM, Martins MA, Flores CA, Cunha FQ, Assreuy-Filho J, *et al.* Mouse paw edema. A new model for inflammation? *Braz J Med Biol Res* 1987, **20**(2)**:** 243-249.

10. Fukawa K, Kawano O, Hibi M, Misaki N, Ohba S, Hatanaka Y. A method for evaluating analgesic agents in rats. *J Pharmacol Methods* 1980, **4**(3)**:** 251-259.

11. Ribeiro RA, Vale ML, Thomazzi SM, Paschoalato AB, Poole S, Ferreira SH, *et al.* Involvement of resident macrophages and mast cells in the writhing nociceptive response induced by zymosan and acetic acid in mice. *Eur J Pharmacol* 2000, **387**(1)**:** 111-118.

12. Jiang XX, Sun YL, Yao J, Cao YM, Kai M, He N, *et al.* Core Scaffold-Inspired Concise Synthesis of Chiral Spirooxindole-Pyranopyrimidines with Broad-Spectrum Anticancer Potency. *Adv Synth Catal* 2012, **354**(5)**:** 917-925.

13. Sun YL, Liu J, Sun T, Zhang XY, Yao J, Kai M, *et al.* Anti-cancer small molecule JP-8g exhibits potent in vivo anti-inflammatory activity. *Sci Rep* 2014, **4:** 4372.

14. Kargman S, Wong E, Greig GM, Falgueyret JP, Cromlish W, Ethier D, *et al.* Mechanism of selective inhibition of human prostaglandin G/H synthase-1 and -2 in intact cells. *Biochem Pharmacol* 1996, **52**(7)**:** 1113-1125.

15. Smith CJ, Zhang Y, Koboldt CM, Muhammad J, Zweifel BS, Shaffer A, *et al.* Pharmacological analysis of cyclooxygenase-1 in inflammation. *Proc Natl Acad Sci U S A* 1998, **95**(22)**:** 13313-13318.

Characterization of Synthesized Compounds

1. NMR, MS and HPLC data of synthesized compounds

(S)-2'-amino-2-oxo-6'-phenylspiro[indoline-3,4'-pyran]-3',5'-dicarbonitrile ***(Q4a)***

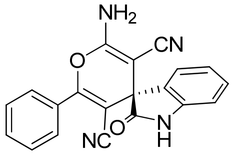

The title compound was isolated by column chromatography (CH_2Cl_2/EtOAc = 4/1) in 94% yield as white solid with a melting point (mp) of 178 °C.

^{1}H NMR (300 MHz, [D^6]DMSO): δ 10.86(s, 1 H), 7.81-7.81(d, *J* = 1.5 Hz, 2 H), 7.56-7.79(m, 5 H), 7.44-7.46(d, *J* = 7.2 Hz, 1 H), 7.30-7.36(m, 1 H), 7.08-7.13(t, *J* = 6.9 Hz, 1 H), 6.92-6.95(d, *J* = 7.8 Hz, 1 H)

^{13}C NMR (75 MHz, [D^6]DMSO): δ 176.4, 159.8, 159.6, 141.5, 132.1, 131.2, 130.1, 129.5, 128.8, 127.9, 125.3, 122.9, 117.0, 115.4, 110.2, 87.5, 53.9, 50.2.

HRMS-ESI (*m/z*): calculated for $C_{20}H_{12}N_4O_2$+Na^+: 363.0852; found: 363.0846, 1.7ppm.

IR: 3427.4, 2206.7, 1726.8, 1668.3, 1627.8, 1470.2, 1412.1, 1318.9, 1262.2, 1152.8, 744.3, 671.0 cm^{-1}.

HPLC: ee was determined by HPLC analysis (Chiralcel AD-H, *i*-PrOH/ Hexane = 30/70, 1.0 mL/min, 246 nm.) Retention time: t_{minor} = 8.89 min, t_{major} = 7.24 min, ee = 99%.

(S)-2'-amino-5-fluoro-2-oxo-6'-phenylspiro[indoline-3,4'-pyran]-3',5'-dicarbonitrile ***(Q4b)***

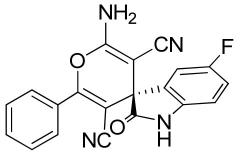

The title compound was isolated by column chromatography (CH_2Cl_2/EtOAc = 4/1) in 78% yield as white solid with an mp of 189 °C.

^{1}H NMR (300 MHz, [D^6]DMSO): δ 10.89(s, 1 H), 7.79-7.82(m, 2 H), 7.72(s, 2 H), 7.51-7.64(m, 4 H), 7.14-7.21(m, 1 H), 6.91-6.96(m, 1 H).

^{13}C NMR (75 MHz, [D^6]DMSO): δ 176.5, 160.0, 159.6, 157.1, 137.7, 132.8, 132.1, 129.5, 128.8, 128.0, 117.0, 116.8, 116.5, 115.3, 113.5, 113.2, 111.1, 86.9, 53.4, 50.7.

HRMS-ESI (*m/z*): calculated for $C_{20}H_{11}FN_4O_2$+Na^+: 381.0758; found:381.0751, 1.8ppm.

IR: 3280.8, 2200.3, 1729.6, 1669.0, 1630.5, 1594.1, 1486.1, 1412.9, 1318.4, 1271.7, 1155.7, 687.9, 599.0 cm^{-1}.

HPLC: ee was determined by HPLC analysis (Chiralcel AD-H, *i*-PrOH/ Hexane = 15/85, 1.0 mL/min, 254 nm.) Retention time: t_{minor} = 18.51 min, t_{major} = 15.98 min, ee = >99%.

(S)-2'-amino-7-fluoro-2-oxo-6'-phenylspiro[indoline-3,4'-pyran]-3',5'-dicarbonitrile ***(Q4c)***

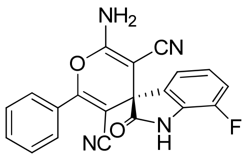

The title compound was isolated by column chromatography (CH_2Cl_2/EtOAc = 4/1) in 92% yield as white solid with an mp of 220 °C.

^{1}H NMR (300 MHz, [D^6]DMSO): δ 11.45(s, 1 H), 7.75-7.83(m, 4 H), 7.56-7.65(m, 3 H), 7.35-7.38(d, J = 7.5Hz, 1 H), 7.26-7.32(t, J = 18.0Hz 1 H), 7.11-7.18(m, 1 H).

^{13}C NMR (75 MHz, [D^6]DMSO): δ 176.8, 160.5, 160.1, 134.4, 132.7, 129.9, 129.3, 128.4, 122.0, 117.5, 117.4, 115.8, 87.4, 53.9, 51.1, 40.8, 40.5, 40.2, 39.9, 39.7, 39.4, 39.1.

HRMS-ESI (*m/z*): calculated for $C_{20}H_{11}FN_4O_2+H^+$: 359.0939; found: 359.0955, 4.4ppm.

IR: 3349.3, 2926.2, 2201.1, 1724.2, 1667.3, 1462.0, 1409.4, 1369.9, 1316.2, 1253.6, 1203.2, 1152.7, 1086.7, 1039.0, 869.4 cm^{-1}.

HPLC: ee was determined by HPLC analysis (Chiralcel AD-H, *i*-PrOH/ Hexane = 30/70, 1.0 mL/min, 254 nm.) Retention time: t_{minor} = 9.15 min, t_{major} = 7.37 min, ee = 93%.

(S)-2'-amino-5-methoxy-2-oxo-6'-phenylspiro[indoline-3,4'-pyran]-3',5'-dicarbonitrile ***(Q4d)***

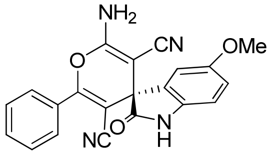

The title compound was isolated by column chromatography (CH_2Cl_2/EtOAc = 4/1) in 84% yield as white solid with an mp of 193 °C.

^{1}H NMR (300 MHz, [D^6]DMSO): δ 10.67(s, 1 H), 7.79-7.82(m, 2 H), 7.58-7.64(m, 5 H), 7.13-7.14(d, J = 2.4 Hz, 1 H), 6.83-6.91(m, 2 H), 3.75(s, 3 H).

^{13}C NMR (75 MHz, [D^6]DMSO): δ 176.3, 159.7, 159.5, 155.7, 134.6, 132.5, 132.1, 129.6, 128.8, 128.0, 117.1, 115.5, 115.0, 111.8, 110.7, 87.5, 55.5, 54.0, 50.7.

HRMS-ESI (*m/z*): calculated for $C_{21}H_{14}N_4O_3+Na^+$: 393.0958; found: 393.0955, 0.8ppm.

IR: 3280.1, 3176.1, 2203.4, 1716.7, 1670.1, 1630.4, 1601.8, 1491.2, 1407.6, 1301.2, 1268.0, 1203.1, 1157.1, 1025.5, 772.6, 686.2, 632.0, 599.3 cm^{-1}.

HPLC: ee was determined by HPLC analysis (Chiralcel AD-H, *i*-PrOH/ Hexane = 15/85, 1.0 mL/min, 254 nm.) Retention time: t_{minor} = 10.43 min, t_{major} = 8.06 min, ee = 94%.

(S)-2'-amino-1-methyl-2-oxo-6'-phenylspiro[indoline-3,4'-pyran]-3',5'-dicarbonitrile ***(Q4e)***

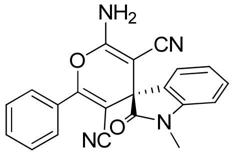

The title compound was isolated by column chromatography (CH_2Cl_2/EtOAc = 8/1) in 80% yield as white solid with an mp of 190 °C.

^{1}H NMR (300 MHz, [D^6]DMSO): δ 7.80-7.82(d, *J* = 6.9 Hz, 2 H), 7.72(s, 2 H), 7.42-7.64(m, 5 H), 7.16-7.22(m, 2 H), 2.23-2.34(d, *J* = 32.4 Hz, 3 H).

^{13}C NMR (75 MHz, [D^6]DMSO): δ 174.8, 160.0, 159.7, 142.9, 132.2, 130.4, 130.2, 129.5, 128.8, 127.9, 125.1, 123.6, 116.9, 115.2, 109.2, 87.3, 53.6, 49.8, 26.6.

HRMS-ESI (*m/z*): calculated for $C_{21}H_{14}N_4O_2$+Na^+: 377.1009; found: 377.1003, 1.6ppm.

IR: 3427.2, 2202.4, 1711.8, 1663.9, 1468.4, 1414.8, 1366.0, 1308.0, 1264.3, 1148.5, 747.7, 576.5 cm^{-1}.

HPLC: ee was determined by HPLC analysis (Chiralcel AD-H, *i*-PrOH/ Hexane = 30/70, 1.0 mL/min, 277 nm.) Retention time: t_{minor} = 11.10 min, t_{major} = 7.53 min, ee = 95%.

*(S)-1-allyl-2'-amino-2-oxo-6'-phenylspiro[indoline-3,4'-pyran]-3',5'-dicarbonitrile **(Q4f)***

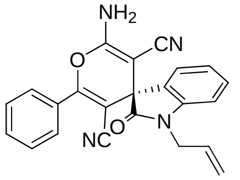

The title compound was isolated by column chromatography (CH_2Cl_2/EtOAc = 8/1) in 99% yield. White solid, mp 206 °C.

^{1}H NMR (300 MHz, [D^6]DMSO): δ 7.80-7.83(m, 2 H), 7.74(s, 1 H), 7.54-7.67(m, 4 H), 7.39-7.44(t, *J* = 7.5 Hz, 1 H), 7.17-7.22(t, *J* = 7.2 Hz, 1 H), 7.04-7.07(d, *J* = 7.8 Hz, 1 H), 5.79-5.91(m, 1 H), 5.15-5.25(m, 2 H), 4.40(s, 2 H);

^{13}C NMR (75 MHz, [D^6]DMSO): δ 174.7, 160.1, 159.7, 142.0, 132.2, 130.9, 130.4, 130.1, 129.5, 128.8, 128.0, 125.2, 123.6, 116.9, 116.4, 115.4, 109.8, 87.2, 53.6, 49.9, 41.9.

HRMS-ESI (*m/z*): calculated for $C_{23}H_{16}N_4O_2$+Na^+:403.1165; found: 403.1169, 1.0ppm.

IR: 3448.4, 3299.9, 2200.6, 1709.0, 11669.5, 1603.1, 1467.3, 1409.4, 1362.2, 1305.9, 1189.5, 1149.0, 921.7, 756.1, 699.4 cm^{-1}.

HPLC: ee was determined by HPLC analysis (Chiralcel AD-H, *i*-PrOH/ Hexane = 30/70, 1.0 mL/min, 277 nm.) Retention time: t_{minor} = 13.69 min, t_{major} = 8.26 min, ee = 95%.

*(S)-2'-amino-2-oxo-1,6'-diphenylspiro[indoline-3,4'-pyran]-3',5'-dicarbonitrile **(Q4g)***

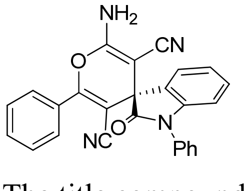

The title compound was isolated by column chromatography (CH_2Cl_2/EtOAc = 8/1) in 87% yield as white solid with an mp of 190 °C.

^{1}H NMR (300 MHz, [D^6]DMSO): δ 7.83-7.86(m, 4 H), 7.51-7.82(m, 7 H), 7.38-7.42(m, 3 H), 7.24-7.29(t, *J* = 7.5 Hz, 1 H), 6.81-6.83(d, *J* = 7.8 Hz, 1 H).

^{13}C NMR (75 MHz, [D^6]DMSO): δ 174.6, 160.0, 159.6, 142.6, 133.6, 132.3, 130.4, 130.3, 130.0, 129.4, 128.9, 128.7, 128.0, 126.4, 125.7, 124.3, 115.3, 109.5, 87.1, 79.1, 53.6, 50.1.

HRMS-ESI (*m/z*): calculated for $C_{26}H_{16}N_4O_2$+Na$^+$: 439.1165; found: 439.1153, 2.7ppm.

IR: 3390.2, 2198.0, 1725.4, 1607.1, 1498.7, 1466.1, 1367.7, 1313.5, 1152.5, 1026.5, 823.9, 757.1, 697.4 cm^{-1}.

HPLC: ee was determined by HPLC analysis (Chiralcel AD-H, *i*-PrOH/ Hexane = 30/70, 1.0 mL/min, 254 nm.) Retention time: t_{minor} = 21.22 min, t_{major} = 10.30 min, ee = 98%.

*(S)-2'-amino-1-benzyl-2-oxo-6'-phenylspiro[indoline-3,4'-pyran]-3',5'-dicarbonitrile (**Q4h**)*

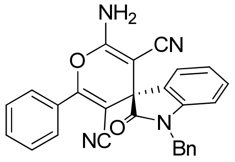

The title compound was isolated by column chromatography (CH_2Cl_2/EtOAc = 8/1) in 89% yield as white soli with an mp of 188 °C.

^{1}H NMR (300 MHz, [D^6]DMSO): δ 7.82-7.84(d, *J* = 6.9 Hz, 2 H), 7.76(s, 2 H), 7.55-7.65(m, 4 H), 7.26-7.39(m, 6 H), 7.15-7.20(t, *J* = 7.5 Hz, 1 H), 6.95-6.98(d, *J* = 7.8 Hz, 1 H), 5.02(s, 2 H.

^{13}C NMR (75 MHz, [D^6]DMSO): δ 175.2, 160.2, 159.8, 142.0, 135.4, 132.2, 130.5, 130.2, 129.5, 128.8, 128.5, 128.0, 127.5, 127.0, 125.3, 123.8, 117.0, 115.4, 109.9, 87.2, 53.7, 49.9, 43.2.

HRMS-ESI (*m/z*): calculated for $C_{27}H_{18}N_4O_2$+Na$^+$: 453.1322; found: 453.1332, 2.2ppm. **IR**: 3338.9, 3176.6, 2205.9, 1705.1, 1673.3, 1609.3, 1364.9, 1305.4, 1147.5, 744.4, 688.5 cm^{-1}.

HPLC: ee was determined by HPLC analysis (Chiralcel AD-H, *i*-PrOH/ Hexane = 30/70, 1.0 mL/min, 254 nm.) Retention time: t_{minor} = 18.51 min, t_{major} = 21.05 min, ee = 96%.

*(S)-1-acetyl-2'-amino-2-oxo-6'-phenylspiro[indoline-3,4'-pyran]-3',5'-dicarbonitrile (**Q4i**)*

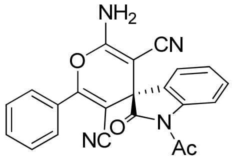

The title compound was isolated by column chromatography (CH_2Cl_2/EtOAc = 8/1) in 85% yield as white solid with an mp of 210 °C.

^{1}H NMR (300 MHz, [D^6]DMSO): δ 8.15-8.18(d, *J* = 8.1 Hz, 1 H), 7.93(s, 2 H), 7.84-7.86(d, *J* = 6.6 Hz, 2 H), 7.58-7.69(m, 4 H), 7.49-7.55(t, *J* = 7.5 Hz, 1 H), 7.38-7.43(t, *J* = 7.5 Hz, 1 H), 2.65(s, 2 H).

^{13}C NMR (75 MHz, [D^6]DMSO): δ 176.3, 170.3, 160.5, 159.8, 139.2, 132.4, 130.5, 129.3, 129.2, 128.8,128.1, 126.4, 125.6, 116.6, 115.9, 115.1, 86.9, 53.8, 50.9, 26.1.

HRMS-ESI (*m/z*): calculated for $C_{22}H_{14}N_4O_3$+Na$^+$: 405.0958; found: 405.0948, 2.5ppm.

IR: 3381.5, 2923.3, 2205.8, 1750.4, 1716.2, 1672.4, 1624.6, 1595.5, 1465.9, 1410.6, 1370.7, 1303.4, 1262.2, 1157.7, 757.9, 694.6, 532.1 cm^{-1}.

HPLC: ee was determined by HPLC analysis (Chiralcel AD-H, *i*-PrOH/ Hexane = 30/70, 1.0 mL/min, 254nm.) Retention time: t_{minor} = 8.23 min, t_{major} = 7.22 min, ee = >99%.

(S)-1-allyl-2'-amino-7-fluoro-2-oxo-6'-phenylspiro[indoline-3,4'-pyran]-3',5'-dicarbonitrile ***(Q4j)***

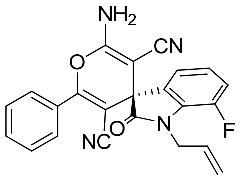

The title compound was isolated by column chromatography (CH_2Cl_2/EtOAc = 8/1) in 93% yield as white solid with an mp of 207 °C.

^{1}H NMR (300 MHz, [D^6]DMSO): δ 7.80-7.83(m, 4 H), 7.56-7.68(m, 3 H), 7.46-7.49(s, 1 H), 7.30-7.36(m, 1 H), 7.20-7.27(m, 1 H), 5.89-5.98(m, 1 H), 5.15-5.21(m, 2 H), 4.47(s, 2 H).

^{13}C NMR (75 MHz, [D^6]DMSO): δ 174.6, 160.3, 159.7, 133.3, 132.3, 131.9, 129.4, 128.8, 128.6, 128.0, 124.9, 124.9, 121.8, 118.3, 118.1, 116.8, 115.6, 115.2, 86.7, 53.3, 50.2, 43.8.

HRMS-ESI (*m/z*): calculated for $C_{23}H_{15}FN_4O_2+Na^+$: 421.1071; found: 421.1077, 1.4ppm.

IR: 3449.9, 3290.7, 3164.9, 2924.6, 2854.9, 2198.0, 1711.5, 1669.4, 1622.0, 1593.7, 1469.1, 1410.2, 1356.3, 1304.9, 1245.2, 1187.4, 1149.0, 989.9, 920.1, 783.9, 744.0, 698.5, 521.7 cm^{-1}.
HPLC: ee was determined by HPLC analysis (Chiralcel AD-H, *i*-PrOH/ Hexane = 30/70, 1.0 mL/min, 277 nm.) Retention time: t_{minor} = 11.37 min, t_{major} = 7.53 min, ee = 95%.

(S)-1-allyl-2'-amino-5-chloro-2-oxo-6'-phenylspiro[indoline-3,4'-pyran]-3',5'-dicarbonitrile ***(Q4k)***

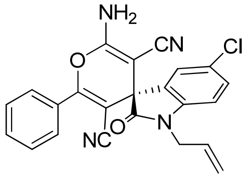

The title compound was isolated by column chromatography (CH_2Cl_2/EtOAc = 8/1) in 80% yield as white solid with an mp of 244 °C.

^{1}H NMR (300 MHz, [D^6]DMSO): δ 7.80-7.84(m, 5 H), 7.71(s, 2 H), 7.56-7.65(m, 3 H), 7.47-7.51(m, 1 H), 7.08-7.11(d, *J* = 8.4 Hz, 1 H), 5.76-5.89(m, 1 H), 5.15-5.24(m, 2 H), 4.41-4.42(d, *J* = 4.5 Hz, 2 H).

^{13}C NMR (75 MHz, [D^6]DMSO): δ 174.5, 160.4, 159.7, 140.9, 132.4, 132.2, 130.7, 130.1, 129.5, 128.8, 128.0, 127.8, 125.7, 116.9, 116.5, 115.4, 111.4, 86.4, 53.1, 50.1, 42.0.

HRMS-ESI (*m/z*): calculated for $C_{23}H_{15}ClN_4O_2+Na^+$:437.0776; found: 437.0766, 2.4ppm.

IR: 3338.6, 3181.7, 2209.1, 1709.2, 1672.5, 1633.8, 1601.3, 1483.2, 1413.6, 1349.6, 1300.5, 1252.9, 1193.0, 1156.0, 1075.9, 820.7, 760.5, 689.6, 586.3, 560.6 cm^{-1}.

HPLC: ee was determined by HPLC analysis (Chiralcel AD-H, *i*-PrOH/ Hexane = 30/70, 1.0 mL/min, 277 nm.) Retention time: t_{minor} = 12.10 min, t_{major} = 6.26 min, ee = >99%.

*(S)-1-allyl-2'-amino-7-chloro-2-oxo-6'-phenylspiro[indoline-3,4'-pyran]-3',5'-dicarbonitrile (**Q4l**)*

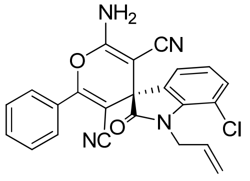

The title compound was isolated by column chromatography (CH_2Cl_2/EtOAc = 8/1) in 87% yield as white solid with an mp of 194 °C.

^{1}H NMR (300 MHz, [D^{6}]DMSO): δ 7.80-7.83(m, 4 H), 7.56-7.65(m, 4 H), 7.43-7.46(m, 1 H), 7.21-7.26(t, *J* = 7.8 Hz, 1 H), 5.92-6.03(m, 1 H), 5.11-5.18(m, 2 H), 4.69-4.69(d, *J* = 1.8 Hz, 2 H).

^{13}C NMR (75 MHz, [D^{6}]DMSO): δ 175.4, 160.4, 159.7, 137.8, 133.5, 132.7, 132.4, 132.3, 129.3, 128.8, 128.0, 125.1, 124.8, 116.8, 115.3, 115.2, 114.6, 86.6, 53.4, 49.7, 43.5.

HRMS-ESI (*m/z*): calculated for $C_{23}H_{15}ClN_4O_2+Na^+$: 437.0776; found: 437.0779, 0.7ppm.

IR: 3392.1, 3309.4, 3189.7, 2924.8, 2207.7, 1713.2, 1669.6, 1593.4, 1454.5, 1409.9, 1356.1, 1314.5, 1154.5, 744.2, 691.3 cm^{-1}.

HPLC: ee was determined by HPLC analysis (Chiralcel AD-H, *i*-PrOH/ Hexane = 30/70, 1.0 mL/min, 254 nm.) Retention time: t_{minor} = 9.06 min, t_{major} = 7.29 min, ee = 97%.

*(S)-1-allyl-2'-amino-5-chloro-7-methyl-2-oxo-6'-phenylspiro[indoline-3,4'-pyran]-3',5'-dicarbonitrile (**Q4m**)*

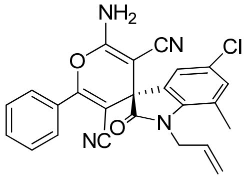

The title compound was isolated by column chromatography (CH_2Cl_2/EtOAc = 8/1) in 99% yield as white solid with an mp of 203 °C.

^{1}H NMR (300 MHz, [D^{6}]DMSO): δ 7.77-7.98(m, 4 H), 7.56-7.71(m, 4 H), 7.30-7.36(m, 1 H), 5.96-6.07(m, 1 H), 5.10-5.20(m, 2 H), 4.56(s, 2 H), 2.47-2.51(m, 3 H.

^{13}C NMR (75 MHz, [D^{6}]DMSO): δ 175.4, 160.4, 159.7, 139.0, 133.3, 133.2, 132.2, 129.5, 128.1, 127.5, 123.5, 122.3, 117.0, 115.5, 114.9, 86.7, 53.6, 49.5, 43.6, 17.3.

HRMS-ESI (*m/z*): calculated for $C_{24}H_{17}ClN_4O_2+Na^+$: 451.0932; found: 451.0925, 1.6ppm.

IR: 3349.4, 3184.9, 2924.4, 2854.8, 2208.9, 1706.4, 1674.3, 1599.6, 1459.6, 1411.5, 1341.7, 1302.7, 1237.0, 1183.4, 1152.6, 688.8, 599.1, 559.0 cm^{-1}.

HPLC: ee was determined by HPLC analysis (Chiralcel AD-H, *i*-PrOH/ Hexane = 30/70, 1.0 mL/min, 254 nm.) Retention time: t_{minor} = 9.11 min, t_{major} = 6.26 min, ee = 99%.

*(S)-1-allyl-2'-amino-5-bromo-2-oxo-6'-phenylspiro[indoline-3,4'-pyran]-3',5'-dicarbonitrile (**Q4n**)*

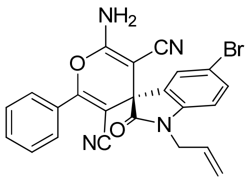

The title compound was isolated by column chromatography (CH_2Cl_2/EtOAc = 8/1) in 90% yield as white solid with an mp of 245 °C.

^{1}H NMR (300 MHz, [D^6]DMSO): δ 7.93(s, 1 H), 7.80-7.92(m, 4 H), 7.56-7.65(m, 4 H), 7.03-7.05(d, J = 8.4 Hz, 1 H), 5.76-5.88(m, 1 H), 5.15-5.23(m, 2 H), 4.40-4.4.14(d, J = 3.6 Hz, 1 H).

^{13}C NMR (75 MHz, [D^6]DMSO): δ 174.4, 160.4, 159.7, 141.3, 133.0, 132.7, 132.2, 130.7, 129.5, 128.8, 128.3, 128.1, 116.9, 116.5, 115.5, 115.4, 111.9, 86.4, 53.1, 50.0, 42.0.

HRMS-ESI (*m/z*): calculated for $C_{23}H_{15}BrN_4O_2$+Na^+: 481.0271; found: 481.0262, 1.9ppm.

IR: 3344.1, 3184.9, 2209.1, 1704.5, 1671.6, 1632.6, 1602.9, 1479.5, 1413.7, 1349.0, 1298.3, 1155.7, 687.3, 582.4 cm^{-1}.

HPLC: ee was determined by HPLC analysis (Chiralcel AD-H, *i*-PrOH/ Hexane = 30/70, 1.0 mL/min, 254 nm.) Retention time: t_{minor} = 12.80 min, t_{major} = 6.47 min, ee = >99%.

(S)-1-allyl-2'-amino-5-methyl-2-oxo-6'-phenylspiro[indoline-3,4'-pyran]-3',5'-dicarbonitrile ***(Q4o)***

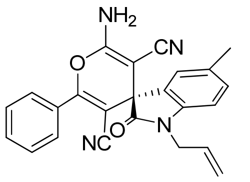

The title compound was isolated by column chromatography (CH_2Cl_2/EtOAc = 8/1) in 92% yield as white solid with an mp of 185 °C.

^{1}H NMR (300 MHz, [D^6]DMSO): δ 7.80-7.83(m, 2 H), 7.71(s, 2 H), 7.56-7.64(m, 3 H), 7.37(s, 1 H), 7.20-7.22(d, J = 7.8 Hz, 1 H), 6.92-6.95(d, J = 8.1 Hz, 1 H), 5.79-5.88(m, 1 H), 5.13-5.24(m, 2 H), 4.37-4.38(d, J = 3.9 Hz, 2 H), 2.33(s, 3 H).

^{13}C NMR (75 MHz, [D^6]DMSO): δ 174.6, 159.9, 159.6, 139.6, 132.8, 132.2, 131.0, 130.5, 130.4, 129.5, 128.8, 128.0, 125.6, 117.0, 116.3, 115.4, 109.6, 87.3, 53.8, 49.9, 41.9, 20.6.

HRMS-ESI (*m/z*): calculated for $C_{24}H_{18}N_4O_2$+Na^+: 417.1322; found: 417.1313, 2.2ppm.

IR:3311.7, 3175.1, 2208.2, 1703.0, 1670.8, 1599.4, 1495.0, 1410.2, 1357.8, 1304.7, 1150.7, 814.4, 693.2 cm^{-1}.

HPLC: ee was determined by HPLC analysis (Chiralcel AD-H, *i*-PrOH/ Hexane = 30/70, 1.0 mL/min, 254 nm.) Retention time: t_{minor} = 11.46 min, t_{major} = 6.42 min, ee = >99%.

(S)-1-allyl-2'-amino-5-methoxy-2-oxo-6'-phenylspiro[indoline-3,4'-pyran]-3',5'-dicarbonitrile ***(Q4p)***

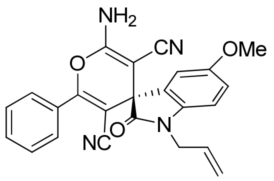

The title compound was isolated by column chromatography (CH_2Cl_2/EtOAc = 8/1) in 99% yield as white solid with an mp of 204 °C.

^{1}H NMR (300 MHz, [D^6]DMSO): δ 7.80-7.83(m, 2 H), 7.71(s, 2 H), 7.55-7.64(m, 3 H), 7.24(s, 1 H), 6.96-6.97(d, *J* = 1.2 Hz, 2 H), 5.76-5.88(m, 1 H), 5.14-5.24(m, 2 H), 4.36-4.37(d, *J* = 2.7 Hz, 2 H), 3.77(s, 3 H).

^{13}C NMR (75 MHz, [D^6]DMSO): δ 174.5, 160.0, 159.7, 156.3, 135.2, 132.1, 131.7, 131.1, 129.6, 128.8, 128.0, 117.0, 116.3, 115.4, 114.8, 112.0, 110.4, 87.2, 55.6, 53.8, 50.3, 41.9.

HRMS-ESI (*m/z*): calculated for $C_{24}H_{18}N_4O_3$+Na^+: 433.1271; found: 433.1275, 0.9ppm.

IR: 3414.4, 3300.7, 3181.6, 2924.3, 2855.1, 2195.1, 1704.7, 1664.6, 1597.0, 1493.0, 1456.4, 1298.8, 1148.5, 1010.0, 769.6, 694.1, 527.8, 479.4 cm^{-1}.

HPLC: ee was determined by HPLC analysis (Chiralcel AD-H, *i*-PrOH/ Hexane = 30/70, 1.0 mL/min, 320 nm.) Retention time: t_{minor} = 17.98 min, t_{major} = 9.39 min, ee = >99%.

*(S)-2'-amino-6'-(3-chlorophenyl)-2-oxospiro[indoline-3,4'-pyran]-3',5'-dicarbonitrile (**Q4q**)*

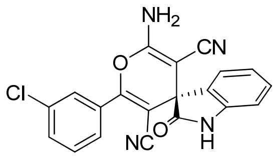

The title compound was isolated by column chromatography (CH_2Cl_2/EtOAc = 4/1) in 75% yield as white solid with an mp of 193 °C.

^{1}H NMR (300 MHz, [D^6]DMSO): δ 10.88(s, 1 H), 7.89(s, 1 H), 7.78-7.81(d, *J* = 7.8 Hz, 1 H), 7.59-7.74(m, 3 H), 7.46-7.49(d, *J* = 7.5 Hz, 1 H), 7.31-7.36(t, *J* = 7.2 Hz, 1 H), 7.09-7.14(t, *J* = 7.5 Hz, 1 H), 6.92-6.95(d, *J* = 7.8 Hz, 1 H).

^{13}C NMR (75 MHz, [D^6]DMSO): δ 176.3, 159.5, 158.2, 141.5, 133.5, 131.9, 131.4, 131.1, 130.8, 130.1, 127.7, 126.7, 126.7, 125.5, 122.9, 116.9, 115.1, 110.2, 88.4, 53.9, 50.2.

HRMS-ESI (*m/z*): calculated for $C_{20}H_{11}ClN_4O_2$+Na^+: 397.0463; found: 397.0458, 1.3ppm.

IR: 3279.6, 3169.6, 2923.7, 2204.3, 1721.3, 1671.9, 1624.0, 1473.1, 1411.8, 1317.3, 1257.3, 1158.7, 747.1, 674.8 cm^{-1}.

HPLC: ee was determined by HPLC analysis (Chiralcel AD-H, *i*-PrOH/ Hexane = 30/70, 1.0 mL/min, 254 nm.) Retention time: t_{minor} = 8.58 min, t_{major} = 6.51 min, ee = >99%.

*(S)-2'-amino-6'-(4-chlorophenyl)-2-oxospiro[indoline-3,4'-pyran]-3',5'-dicarbonitrile (**Q4r**)*

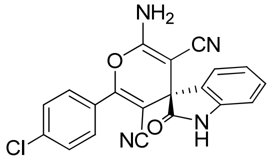

The title compound was isolated by column chromatography (CH_2Cl_2/EtOAc = 4/1) in 78% yield as white solid with an mp of 180 °C.

^{1}H NMR (300 MHz, [D^6]DMSO): δ 10.87(s, 1 H), 7.82-7.85(d, J = 8.7 Hz, 2 H), 7.67-7.70(m, 4 H), 7.45-7.47(d, J = 7.5 Hz, 1 H), 7.33(s, 1 H), 7.11-7.13(d, J = 7.5 Hz, 1 H), 6.92-6.95(d, J = 7.5 Hz, 1 H).

^{13}C NMR (75 MHz, [D^6]DMSO): δ 176.3, 159.5, 158.7, 141.5, 136.9, 131.1, 130.1, 129.8, 129.0, 128.3, 125.4, 122.9, 117.0, 115.2, 110.2, 87.9, 53.9, 50.2.

HRMS-ESI (*m/z*): calculated for $C_{20}H_{11}ClN_4O_2$+Na^+: 397.0463; found: 397.0461, 0.5ppm.

IR: 3302.8, 3192.8, 2924.4, 2201.7, 1726.7, 1669.6, 1591.6, 1470.9, 1402.9, 1317.5, 1154.9, 1093.9, 741.0, 672.9 cm^{-1}.

HPLC: ee was determined by HPLC analysis (Chiralcel AD-H, *i*-PrOH/ Hexane = 30/70, 1.0 mL/min, 254 nm.) Retention time: t_{minor} = 16.05 min, t_{major} = 8.03 min, ee = >99%.

(S)-2'-amino-2-oxo-6'-m-tolylspiro[indoline-3,4'-pyran]-3',5'-dicarbonitrile ***(Q4s)***

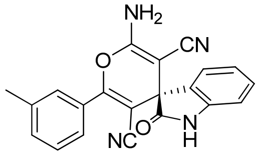

The title compound was isolated by column chromatography (CH_2Cl_2/EtOAc = 4/1) in 88% yield as white solid with an mp of 198 °C.

^{1}H NMR (300 MHz, [D^6]DMSO): δ 10.86(s, 1 H), 7.57-7.65(m, 4 H), 7.43-7.49(m, 3 H), 7.30-7.36(m, 1 H), 7.08-7.13(m, 1 H), 6.92-6.95(d, J = 7.8 Hz, 2 H), 2.38(s, 3 H).

^{13}C NMR (75 MHz, [D^6]DMSO): δ 176.4, 159.9, 159.6, 141.5, 138.3, 132.7, 131.3, 130.1, 129.5, 128.7, 128.2, 125.3, 125.1, 122.9, 117.0, 115.4, 110.2, 87.4, 53.9, 50.2, 20.8.

HRMS-ESI (*m/z*): calculated for $C_{21}H_{14}N_4O_2$+Na^+: 377.1009; found: 377.1000, 2.4ppm.

IR: 3303.1, 3169.5, 2923.5, 2204.6, 1721.0, 1667.9, 1623.3, 1470.6, 1411.1, 1315.1, 1144.3, 741.2, 674.3 cm^{-1}.

HPLC: ee was determined by HPLC analysis (Chiralcel AD-H, *i*-PrOH/ Hexane = 30/70, 1.0 mL/min, 254 nm.) Retention time: t_{minor} = 7.79 min, t_{major} = 6.85 min, ee = >99%.

(S)-2'-amino-2-oxo-6'-p-tolylspiro[indoline-3,4'-pyran]-3',5'-dicarbonitrile ***(Q4t)***

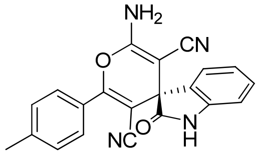

The title compound was isolated by column chromatography (CH_2Cl_2/EtOAc = 4/1) in 90% yield as white solid with an mp of 173 °C.

^{1}H NMR (300 MHz, [D^6]DMSO): δ 10.85(s, 1 H), 7.65-7.72(m, 4 H), 7.38-7.44(m, 3 H), 7.30-7.33(m, 1 H), 7.08-7.13(t, J = 7.5 Hz, 1 H), 6.92-6.94(d, J = 7.5 Hz, 2 H), 2.39(s, 3 H).

^{13}C NMR (75 MHz, [D^6]DMSO): δ 176.5, 159.7, 159.6, 142.4, 141.5, 131.3, 130.0, 129.3, 127.8, 126.7, 125.3, 122.9, 117.1, 115.6, 110.2, 86.7, 53.9, 50.2, 21.0.

HRMS-ESI (*m/z*): calculated for $C_{21}H_{14}N_4O_2+Na^+$: 377.1009; found: 377.1001, 2.1ppm.

IR: 3342.9, 3180.5, 2205.3, 1720.3, 1667.2, 1619.5, 1470.1, 1407.8, 1310.5, 1151.3, 677.1 cm^{-1}.

HPLC: ee was determined by HPLC analysis (Chiralcel AD-H, *i*-PrOH/ Hexane = 30/70, 1.0 mL/min, 254 nm.) Retention time: t_{minor} = 9.62min, t_{major} = 10.65 min, ee = 90%.

2. HPLC Chromatograms of racemic and chiral products

(*S*)-2'-amino-2-oxo-6'-phenylspiro[indoline-3,4'-pyran]-3',5'-dicarbonitrile (**Q4a**)

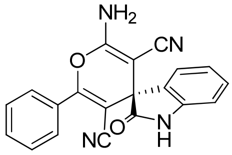

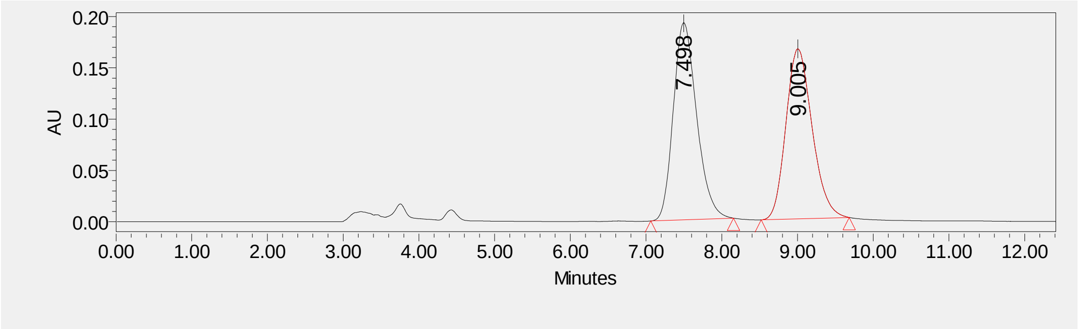

Entry	Retention Time	Area	Area (%)	Height	Int Type	Peak Type
1	7.498	4113817	50.78	192104	bb	Unknown
2	9.005	3988145	49.22	165690	bb	Unknown

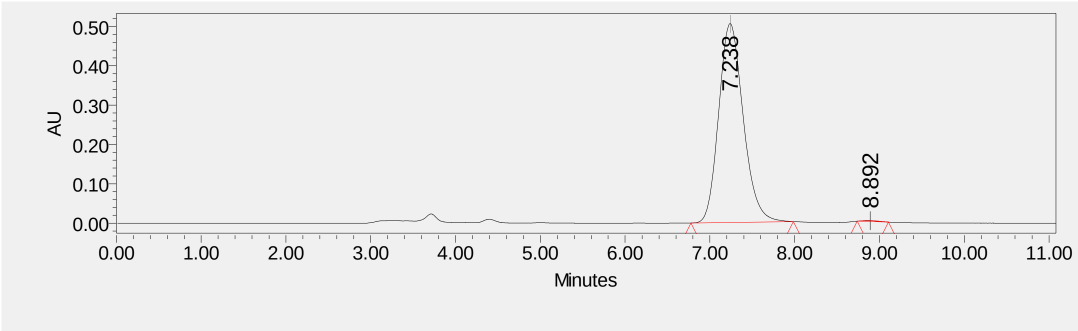

Entry	Retention Time	Area	Area (%)	Height	Int Type	Peak Type
1	7.238	10154857	99.70	505899	bb	Unknown
2	8.892	30387	0.30	2279	bb	Unknown

(*S*)-2'-amino-5-fluoro-2-oxo-6'-phenylspiro[indoline-3,4'-pyran]-3',5'-dicarbonitrile (**Q4b**)

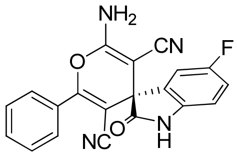

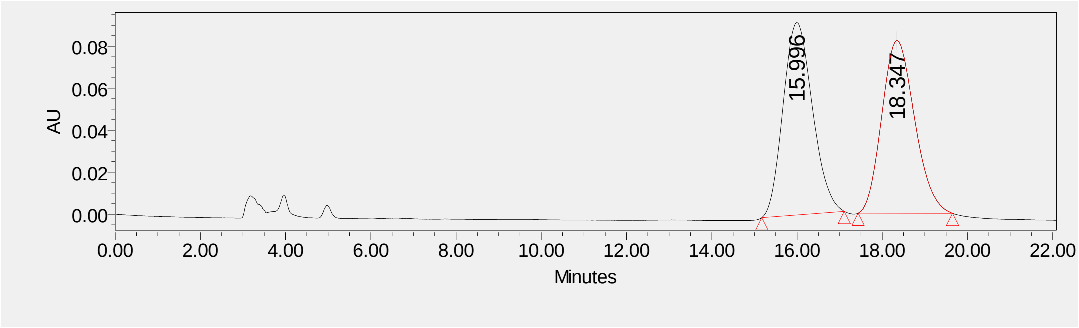

Entry	Retention Time	Area	Area (%)	Height	Int Type	Peak Type
1	15.996	4400793	50.03	91632	bb	Unknown
2	18.347	4396005	49.97	82231	bb	Unknown

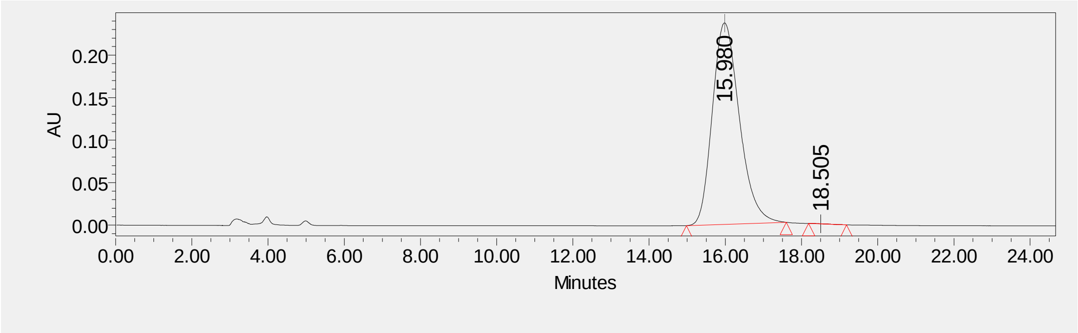

Entry	Retention Time	Area	Area (%)	Height	Int Type	Peak Type
1	15.980	11693491	99.96	236932	bb	Unknown
2	18.505	4287	0.04	169	bb	Unknown

(*S*)-2'-amino-7-fluoro-2-oxo-6'-phenylspiro[indoline-3,4'-pyran]-3',5'-dicarbonitrile (**Q4c**)

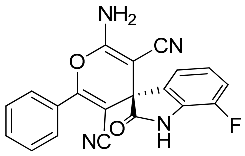

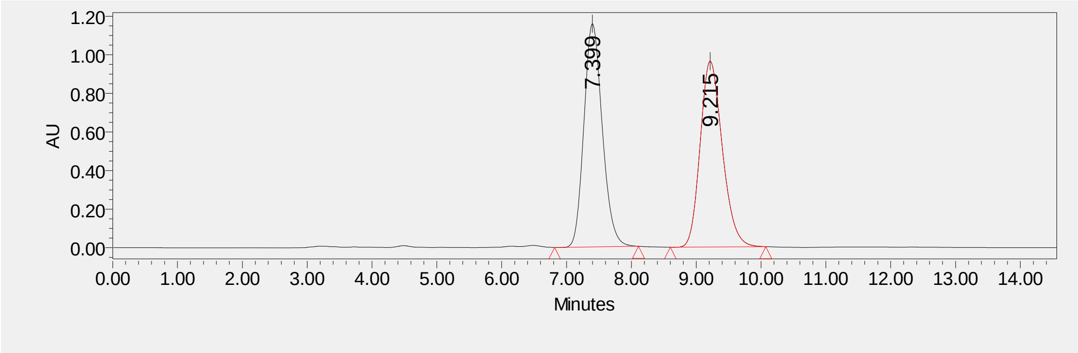

Entry	Retention Time	Area	Area (%)	Height	Int Type	Peak Type
1	7.399	22810800	49.95	1156418	bb	Unknown
2	9.215	22858331	50.05	963370	bb	Unknown

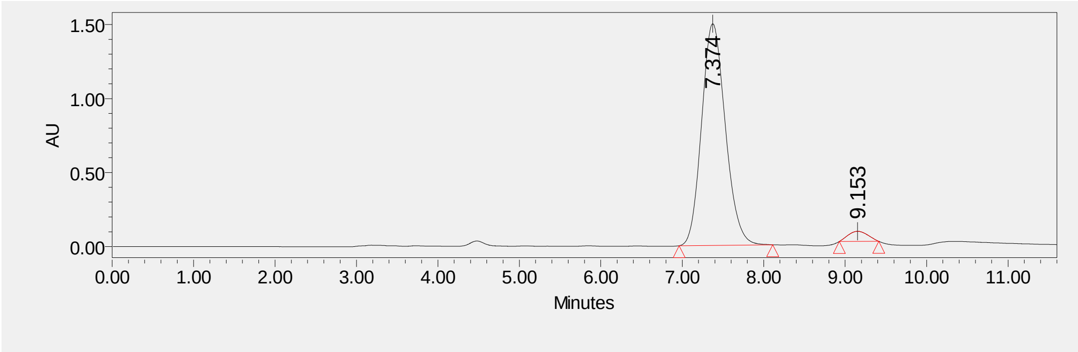

Entry	Retention Time	Area	Area (%)	Height	Int Type	Peak Type
1	7.374	30287876	96.34	1497732	bb	Unknown
2	9.153	1151154	3.66	67973	bb	Unknown

(*S*)-2'-amino-5-methoxy-2-oxo-6'-phenylspiro[indoline-3,4'-pyran]-3',5'-dicarbonitrile (**Q4d**)

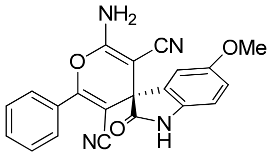

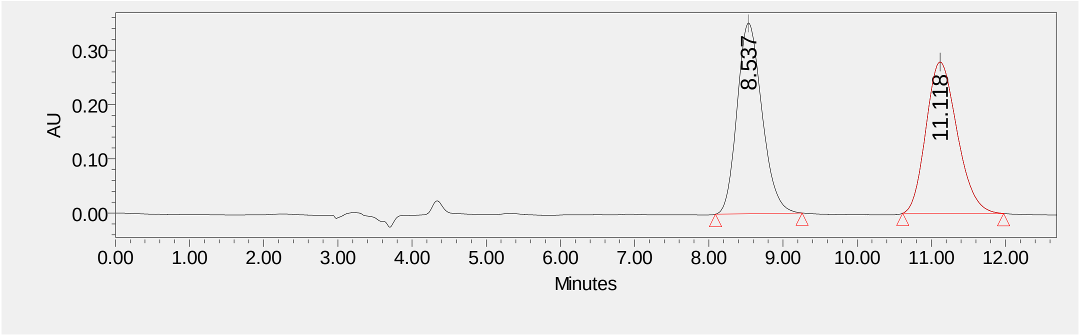

Entry	Retention Time	Area	Area (%)	Height	Int Type	Peak Type
1	8.537	8196493	50.21	351215	bb	Unknown
2	11.118	8127534	49.79	278759	bb	Unknown

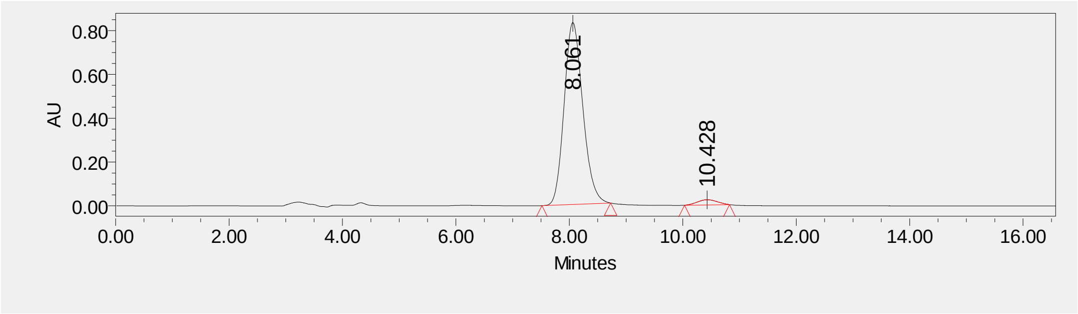

Entry	Retention Time	Area	Area (%)	Height	Int Type	Peak Type
1	8.061	18993096	97.09	830456	bb	Unknown
2	10.428	569783	2.91	23444	bb	Unknown

(*S*)-2'-amino-1-methyl-2-oxo-6'-phenylspiro[indoline-3,4'-pyran]-3',5'-dicarbonitrile (**Q4e**)

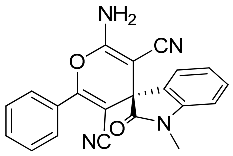

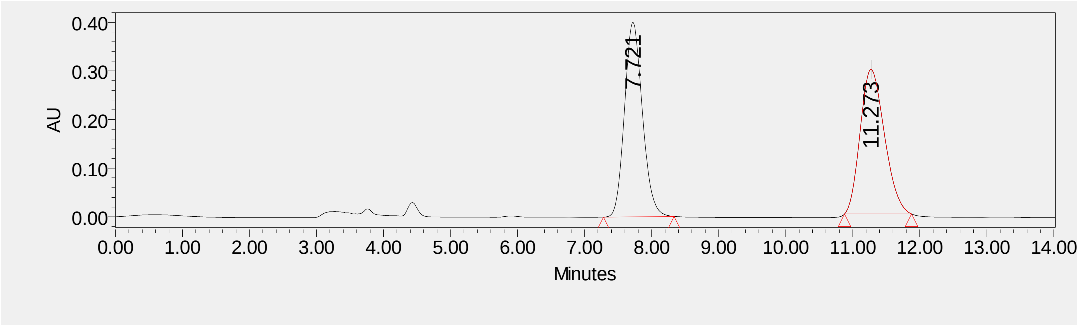

Entry	Retention Time	Area	Area (%)	Height	Int Type	Peak Type
1	7.721	7434942	49.87	399758	bb	Unknown
2	11.273	7474552	50.13	297092	bb	Unknown

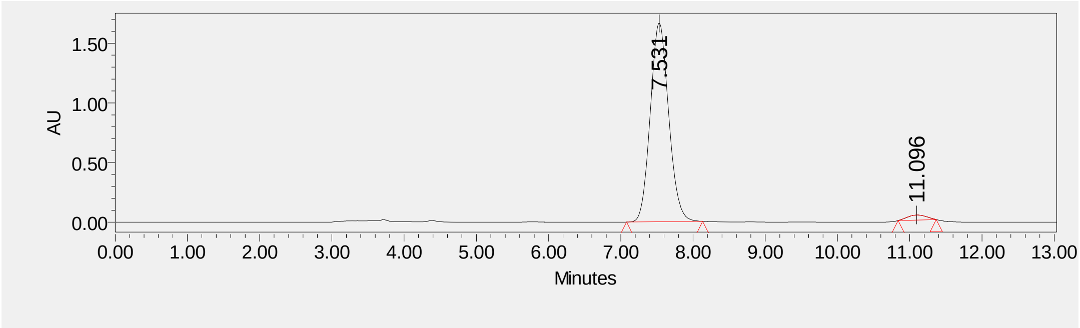

Entry	Retention Time	Area	Area (%)	Height	Int Type	Peak Type
1	7.531	29511293	97.42	1664904	bb	Unknown
2	11.096	781505	2.58	42574	bb	Unknown

(*S*)-1-allyl-2'-amino-2-oxo-6'-phenylspiro[indoline-3,4'-pyran]-3',5'-dicarbonitrile (**Q4f**)

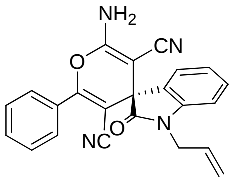

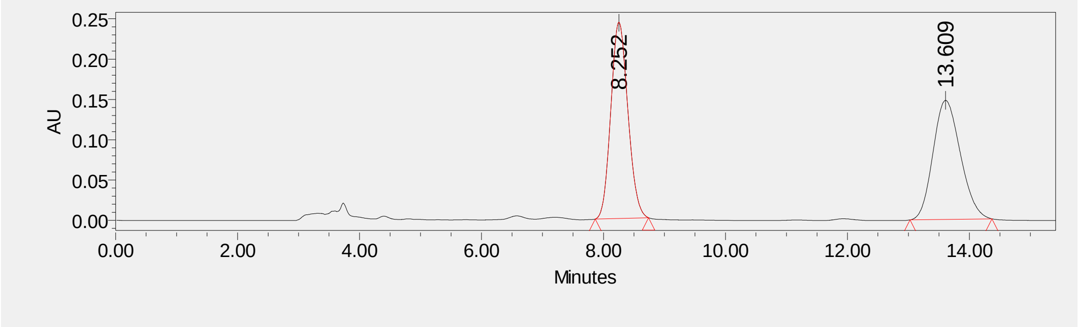

Entry	Retention Time	Area	Area (%)	Height	Int Type	Peak Type
1	8.252	4725852	50.64	243411	bb	Unknown
2	13.609	4606413	49.36	147655	bb	Unknown

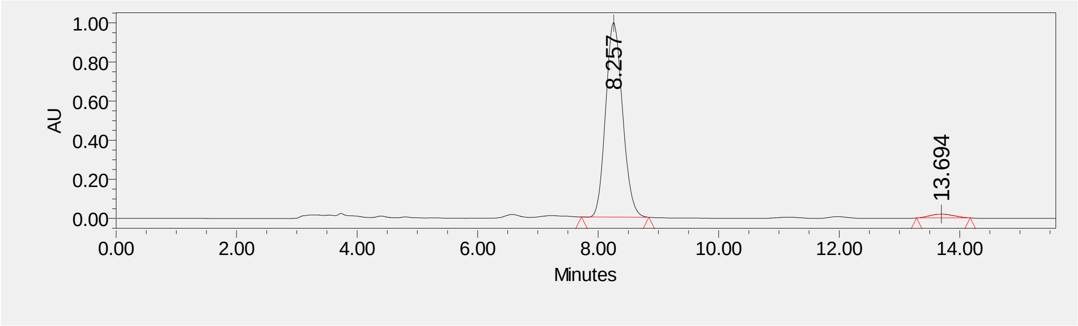

Entry	Retention Time	Area	Area (%)	Height	Int Type	Peak Type
1	8.257	19377892	97.30	994839	bb	Unknown
2	13.694	537211	2.70	19422	bb	Unknown

(*S*)-2'-amino-2-oxo-1,6'-diphenylspiro[indoline-3,4'-pyran]-3',5'-dicarbonitrile (**Q4g**)

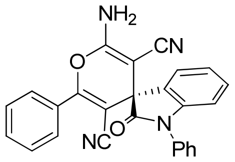

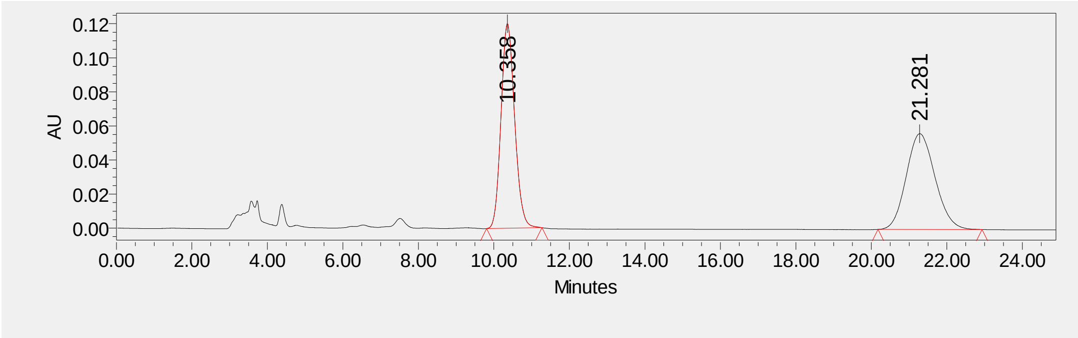

Entry	Retention Time	Area	Area (%)	Height	Int Type	Peak Type
1	10.358	2996667	50.09	120108	bb	Unknown
2	21.281	2986100	49.91	56230	bb	Unknown

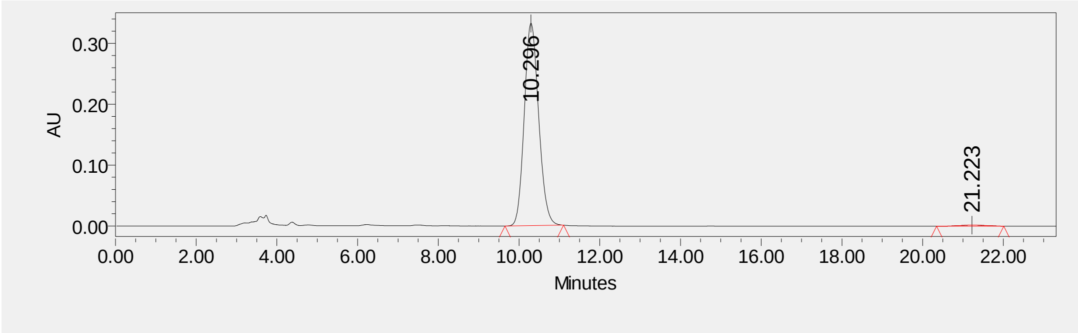

Entry	Retention Time	Area	Area (%)	Height	Int Type	Peak Type
1	10.296	8214774	99.01	332751	bb	Unknown
2	21.223	81858	0.99	1722	bb	Unknown

(*S*)-2'-amino-1-benzyl-2-oxo-6'-phenylspiro[indoline-3,4'-pyran]-3',5'-dicarbonitrile (**Q4h**)

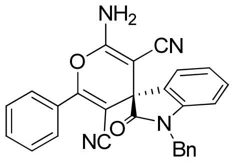

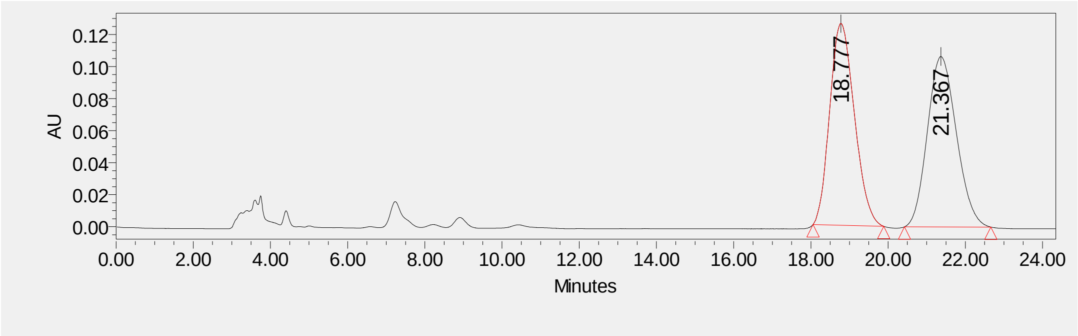

Entry	Retention Time	Area	Area (%)	Height	Int Type	Peak Type
1	18.777	5599946	50.23	125983	bb	Unknown
2	21.367	5549693	49.77	106373	bb	Unknown

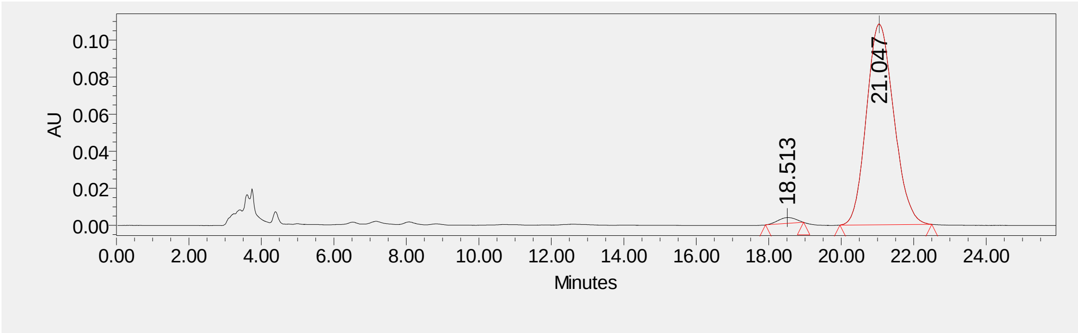

Entry	Retention Time	Area	Area (%)	Height	Int Type	Peak Type
1	18.513	106921	1.85	3170	bb	Unknown
2	21.047	5678297	98.15	108365	bb	Unknown

(*S*)-1-acetyl-2'-amino-2-oxo-6'-phenylspiro[indoline-3,4'-pyran]-3',5'-dicarbonitrile (**Q4i**)

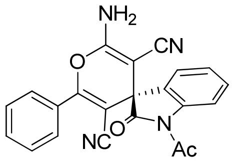

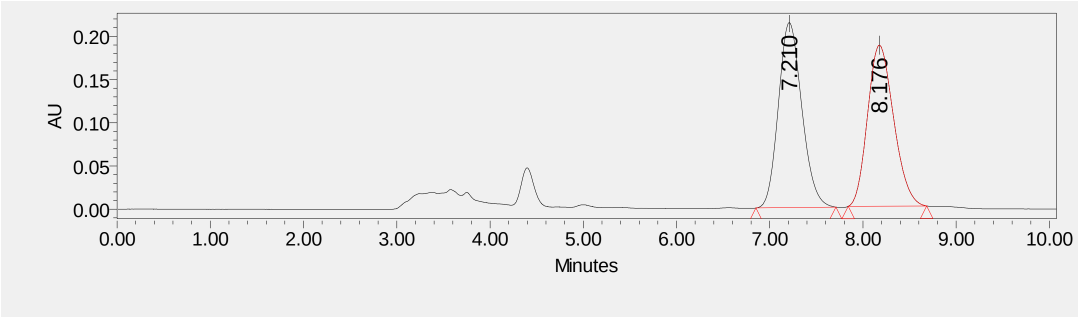

Entry	Retention Time	Area	Area (%)	Height	Int Type	Peak Type
1	7.210	3734426	50.82	213985	bb	Unknown
2	8.176	3613869	49.18	186227	bb	Unknown

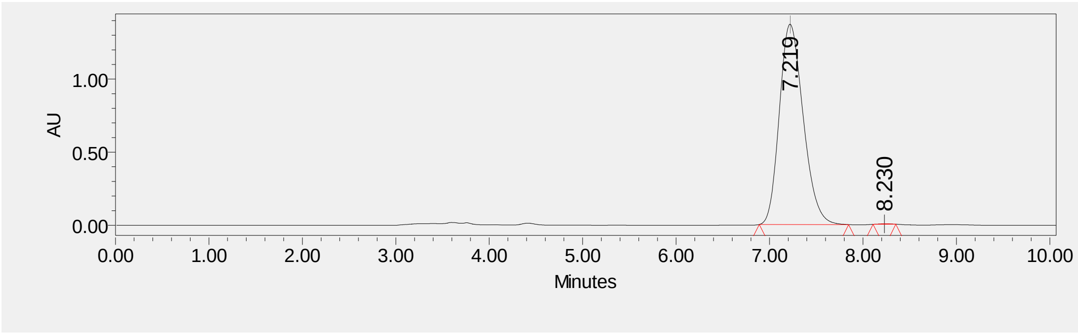

Entry	Retention Time	Area	Area (%)	Height	Int Type	Peak Type
1	7.219	24282977	99.89	1370921	bb	Unknown
2	8.230	27412	0.11	2991	bb	Unknown

(*S*)-1-allyl-2'-amino-7-fluoro-2-oxo-6'-phenylspiro[indoline-3,4'-pyran]-3',5'-dicarbonitrile (**Q4j**)

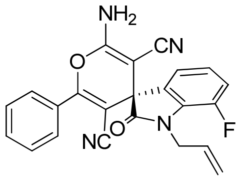

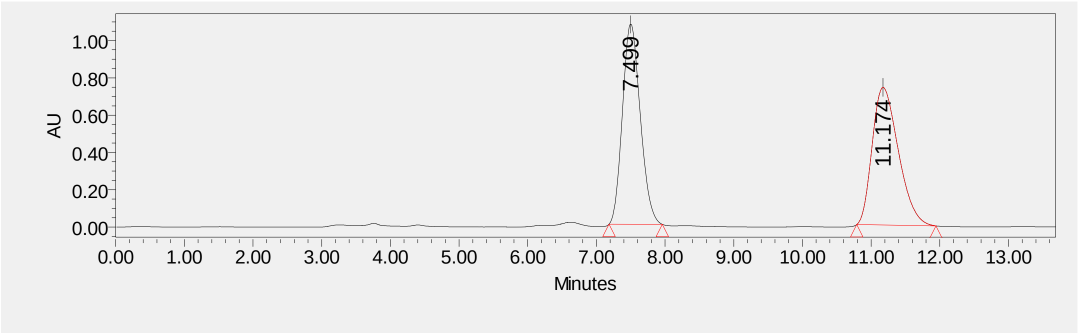

Entry	Retention Time	Area	Area (%)	Height	Int Type	Peak Type
1	7.499	19380517	49.84	1074327	bb	Unknown
2	11.174	19506439	50.16	737355	bb	Unknown

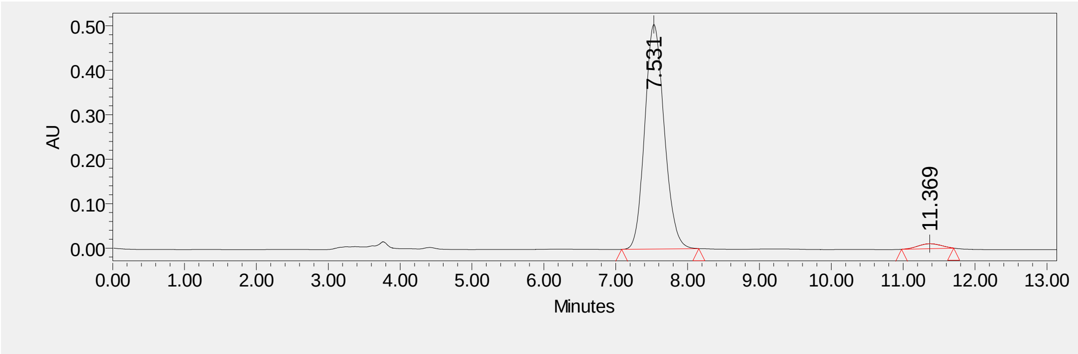

Entry	Retention Time	Area	Area (%)	Height	Int Type	Peak Type
1	7.531	9527582	97.48	505431	bb	Unknown
2	11.369	246001	2.52	11086	bb	Unknown

(*S*)-1-allyl-2'-amino-5-chloro-2-oxo-6'-phenylspiro[indoline-3,4'-pyran]-3',5'-dicarbonitrile (**Q4k**)

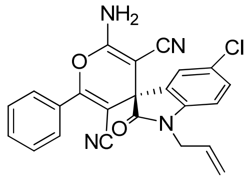

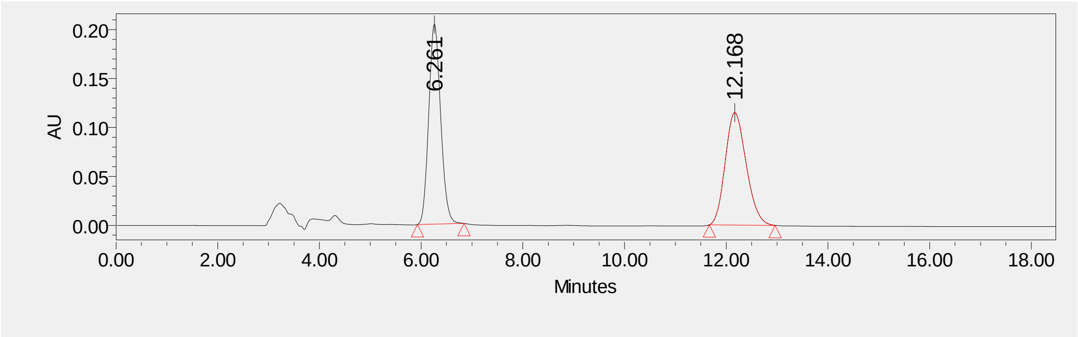

Entry	Retention Time	Area	Area (%)	Height	Int Type	Peak Type
1	6.261	3334836	50.38	204339	bb	Unknown
2	12.168	3284913	49.62	114773	bb	Unknown

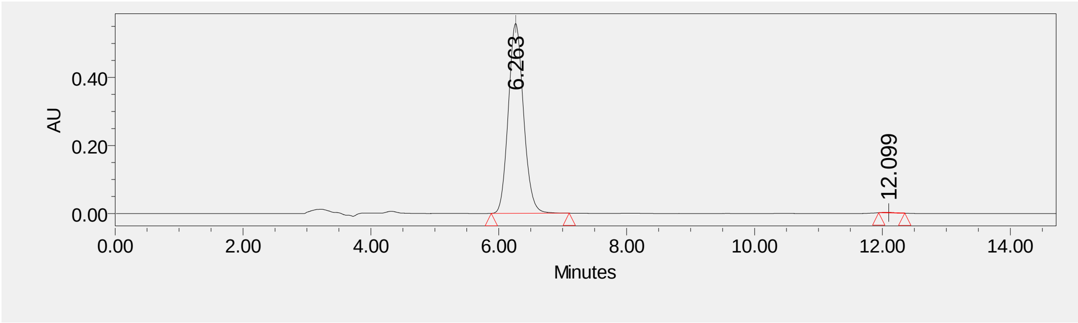

Entry	Retention Time	Area	Area (%)	Height	Int Type	Peak Type
1	6.263	9195063	99.83	558305	bb	Unknown
2	12.099	15913	0.17	1092	bb	Unknown

(*S*)-1-allyl-2'-amino-7-chloro-2-oxo-6'-phenylspiro[indoline-3,4'-pyran]-3',5'-dicarbonitrile (**Q4l**)

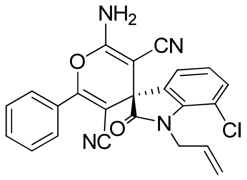

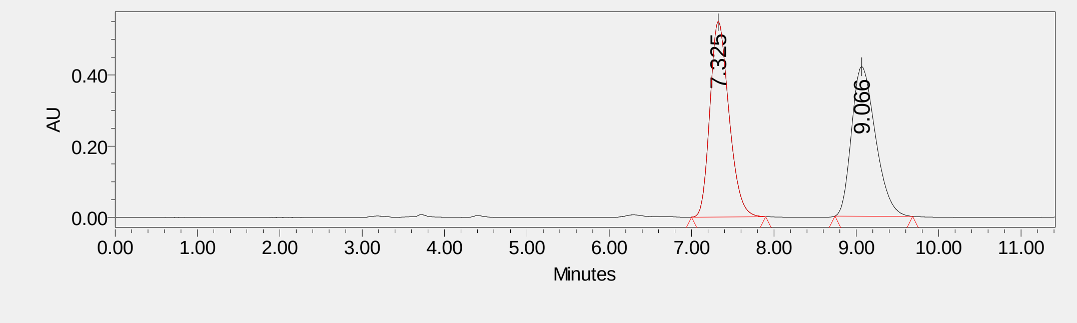

Entry	Retention Time	Area	Area (%)	Height	Int Type	Peak Type
1	7.325	8816025	50.99	548589	bb	Unknown
2	9.066	8472885	49.01	419684	bb	Unknown

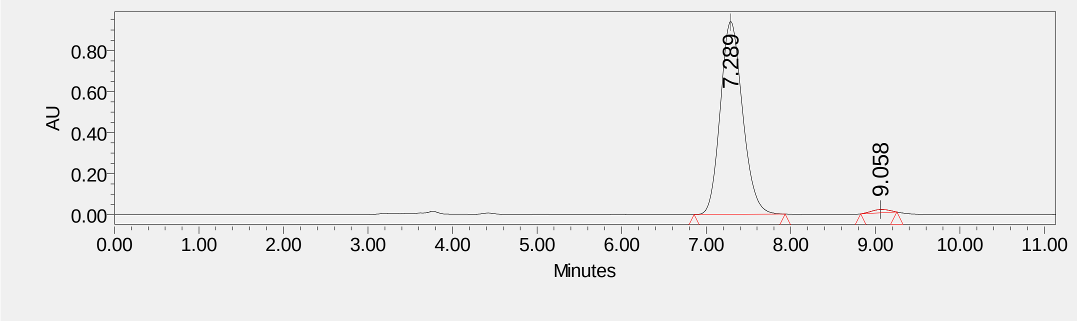

Entry	Retention Time	Area	Area (%)	Height	Int Type	Peak Type
1	7.289	17191849	98.64	939937	bb	Unknown
2	9.058	236284	1.36	16243	bb	Unknown

(*S*)-1-allyl-2'-amino-5-chloro-7-methyl-2-oxo-6'-phenylspiro[indoline-3,4'-pyran]-3',5'-dicarbonitrile (**Q4m**)

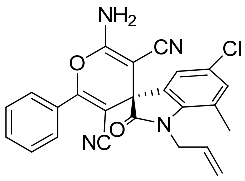

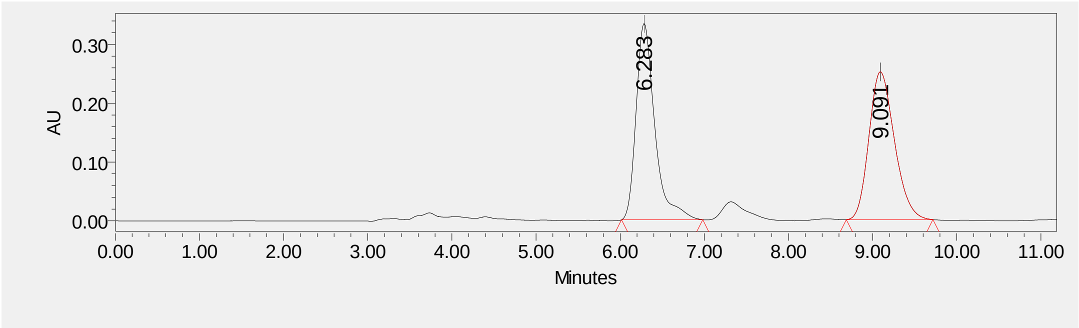

Entry	Retention Time	Area	Area (%)	Height	Int Type	Peak Type
1	6.283	5167061	49.43	333746	bb	Unknown
2	9.091	5286817	50.57	251280	bb	Unknown

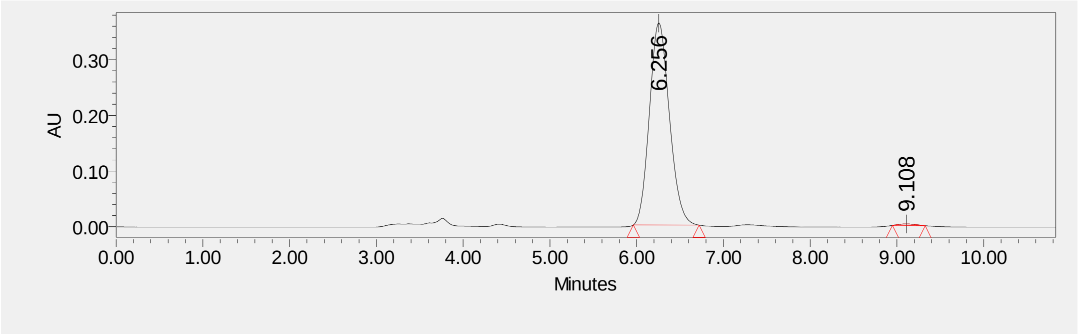

Entry	Retention Time	Area	Area (%)	Height	Int Type	Peak Type
1	6.256	5808701	99.35	362796	bb	Unknown
2	9.108	38213	0.65	2799	bb	Unknown

(*S*)-1-allyl-2'-amino-5-bromo-2-oxo-6'-phenylspiro[indoline-3,4'-pyran]-3',5'-dicarbonitrile (**Q4n**)

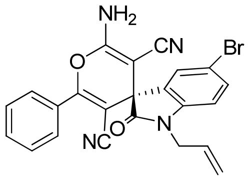

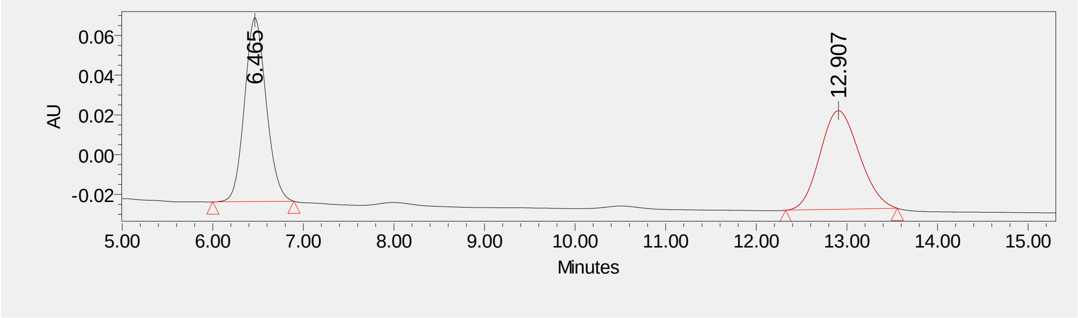

Entry	Retention Time	Area	Area (%)	Height	Int Type	Peak Type
1	6.465	1540523	50.71	92449	bb	Unknown
2	12.907	1497145	49.29	49673	bb	Unknown

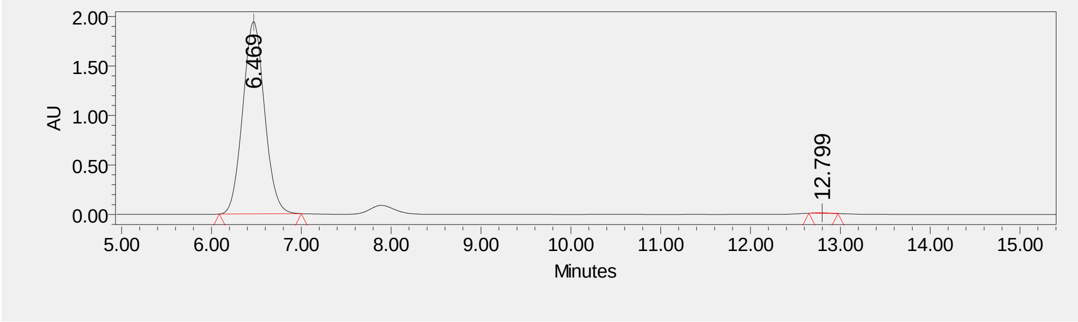

Entry	Retention Time	Area	Area (%)	Height	Int Type	Peak Type
1	6.469	32660271	99.85	1942448	bb	Unknown
2	12.799	48008	0.15	3874	bb	Unknown

(*S*)-1-allyl-2'-amino-5-methyl-2-oxo-6'-phenylspiro[indoline-3,4'-pyran]-3',5'-dicarbonitrile (**Q4o**)

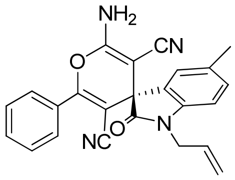

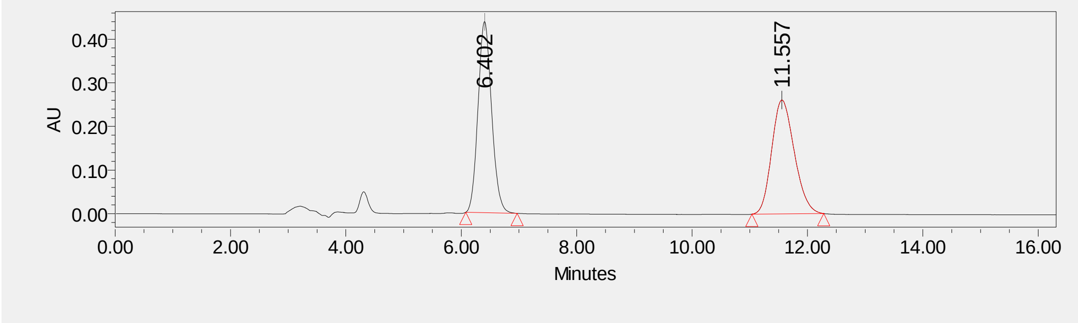

Entry	Retention Time	Area	Area (%)	Height	Int Type	Peak Type
1	6.402	7168957	50.23	438382	bb	Unknown
2	11.557	7103001	49.77	261188	bb	Unknown

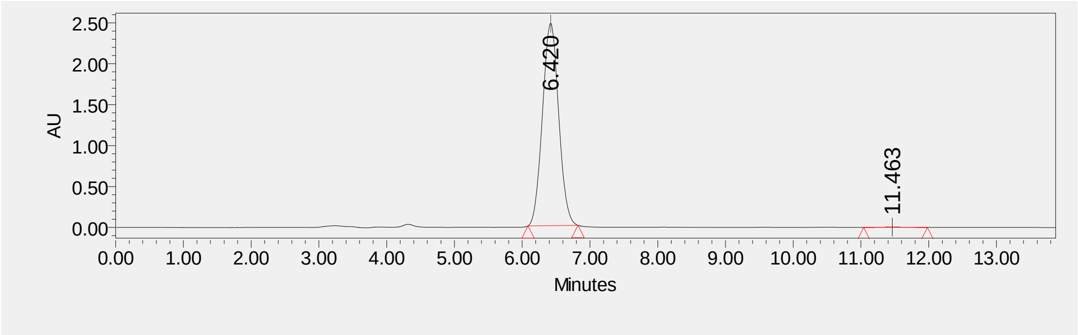

Entry	Retention Time	Area	Area (%)	Height	Int Type	Peak Type
1	6.420	41075254	99.70	2470262	bb	Unknown
2	11.463	121624	0.30	4974	bb	Unknown

(*S*)-1-allyl-2'-amino-5-methoxy-2-oxo-6'-phenylspiro[indoline-3,4'-pyran]-3',5'-dicarbonitrile (**Q4p**)

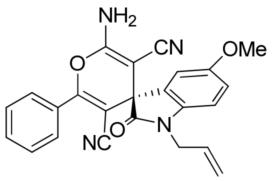

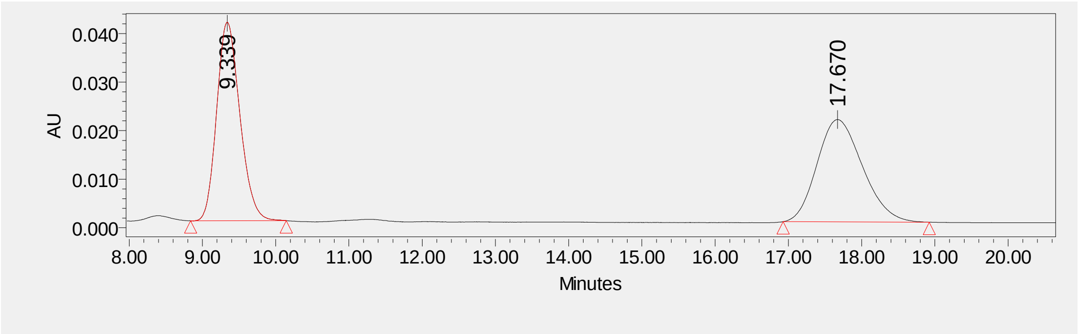

Entry	Retention Time	Area	Area (%)	Height	Int Type	Peak Type
1	9.339	898735	49.73	40910	bb	Unknown
2	17.670	908321	50.27	21070	bb	Unknown

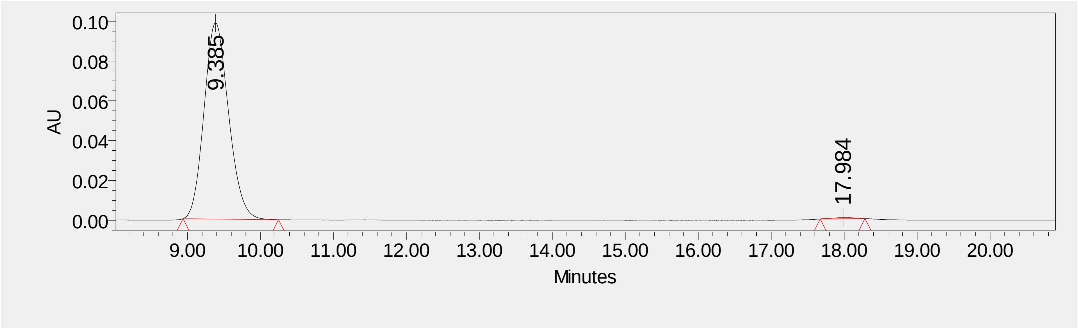

Entry	Retention Time	Area	Area (%)	Height	Int Type	Peak Type
1	9.385	2300821	99.47	98537	bb	Unknown
2	17.984	12173	0.53	533	bb	Unknown

(*S*)-2'-amino-6'-(3-chlorophenyl)-2-oxospiro[indoline-3,4'-pyran]-3',5'-dicarbonitrile (**Q4q**)

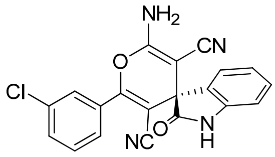

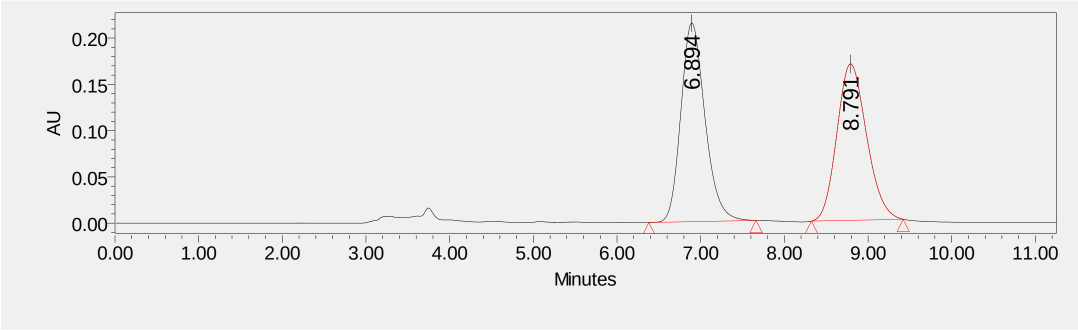

Entry	Retention Time	Area	Area (%)	Height	Int Type	Peak Type
1	6.894	4298030	50.78	214895	bb	Unknown
2	8.791	4165319	49.22	169093	bb	Unknown

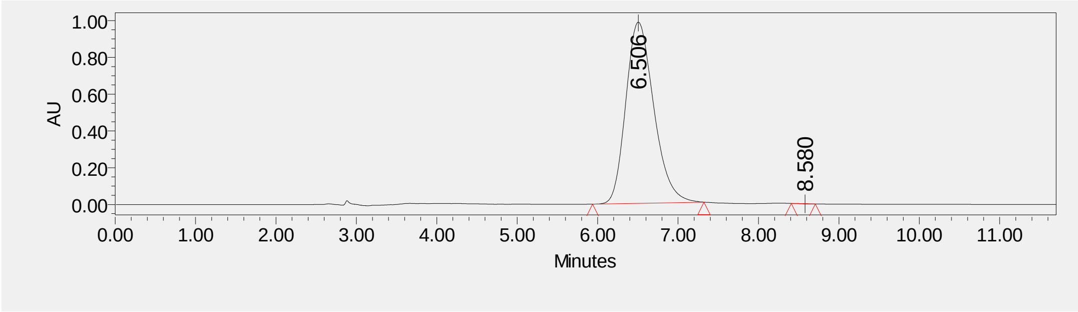

Entry	Retention Time	Area	Area (%)	Height	Int Type	Peak Type
1	6.506	23844261	99.99	985742	bb	Unknown
2	8.580	3126	0.01	-280	bb	Unknown

(*S*)-2'-amino-6'-(4-chlorophenyl)-2-oxospiro[indoline-3,4'-pyran]-3',5'-dicarbonitrile (**Q4r**)

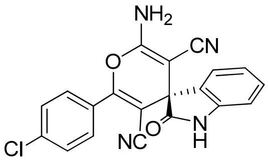

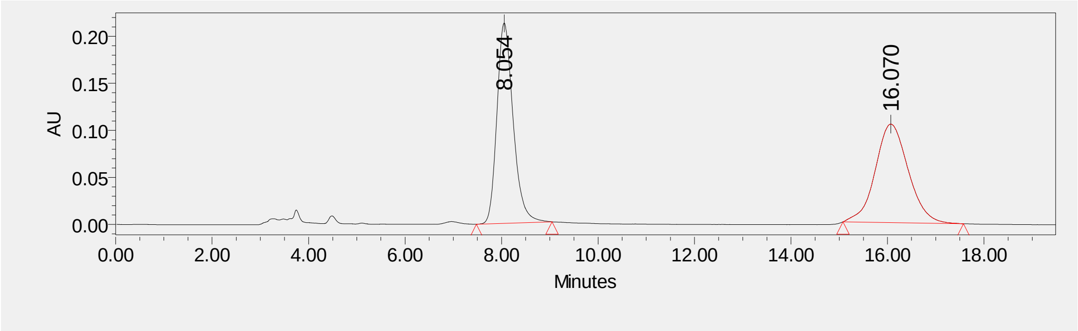

Entry	Retention Time	Area	Area (%)	Height	Int Type	Peak Type
1	8.054	4972769	50.01	212970	bb	Unknown
2	16.070	4971552	49.99	104655	bb	Unknown

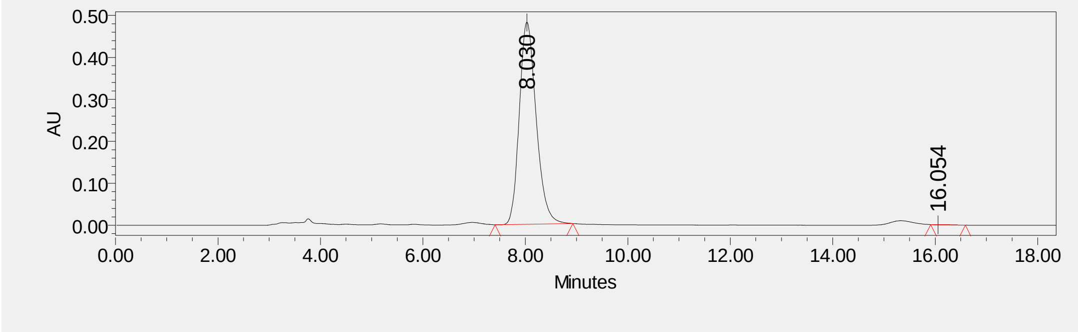

Entry	Retention Time	Area	Area (%)	Height	Int Type	Peak Type
1	8.030	10995890	99.97	481630	bb	Unknown
2	16.054	3585	0.03	-158	bb	Unknown

(*S*)-2'-amino-2-oxo-6'-m-tolylspiro[indoline-3,4'-pyran]-3',5'-dicarbonitrile (**Q4s**)

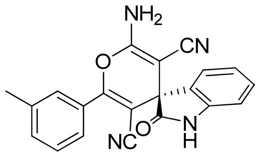

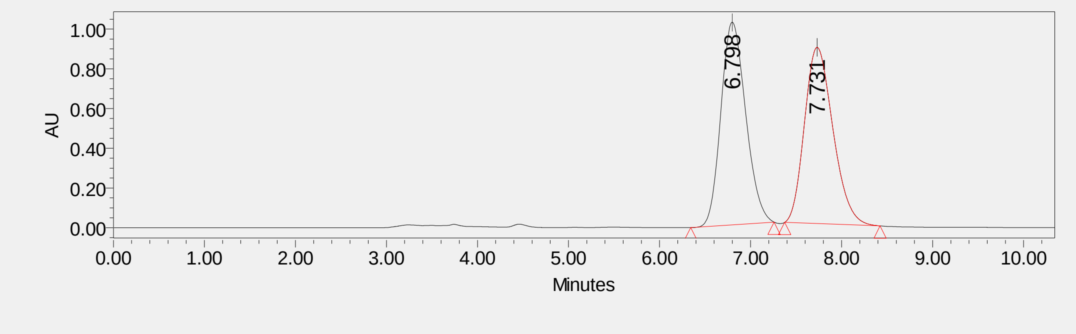

Entry	Retention Time	Area	Area (%)	Height	Int Type	Peak Type
1	6.798	19317190	50.06	1020839	bb	Unknown
2	7.731	19272017	49.94	887226	bb	Unknown

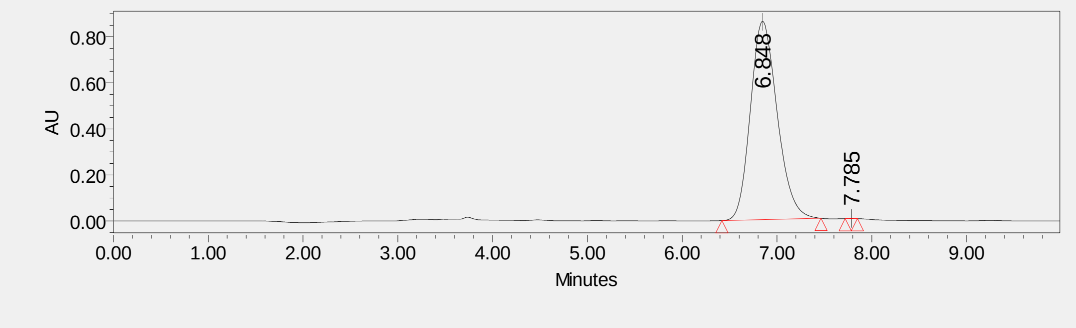

Entry	Retention Time	Area	Area (%)	Height	Int Type	Peak Type
1	6.848	16944656	99.98	860842	bb	Unknown
2	7.785	2666	0.02	533	bb	Unknown

(*S*)-2'-amino-2-oxo-6'-p-tolylspiro[indoline-3,4'-pyran]-3',5'-dicarbonitrile (**Q4t**)

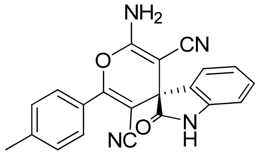

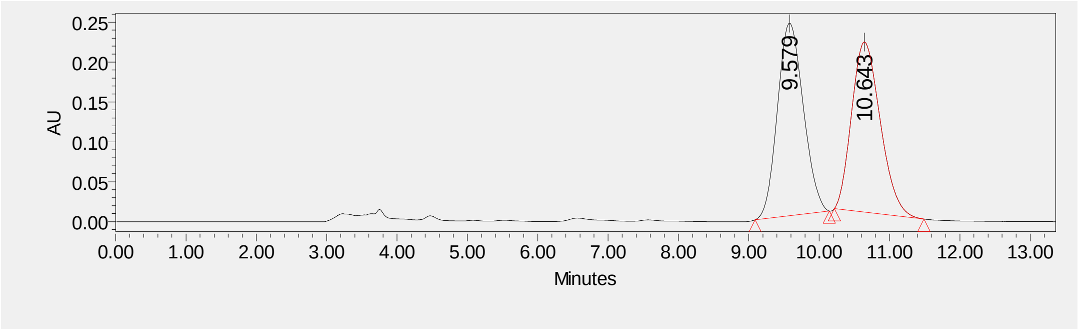

Entry	Retention Time	Area	Area (%)	Height	Int Type	Peak Type
1	9.579	6166421	50.56	241321	bb	Unknown
2	10.643	6029328	49.44	213006	bb	Unknown

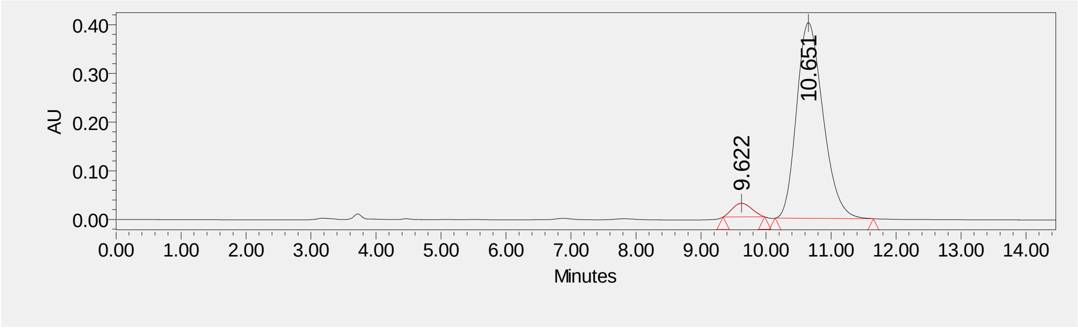

Entry	Retention Time	Area	Area (%)	Height	Int Type	Peak Type
1	9.622	579309	4.85	28052	bb	Unknown
2	10.651	11356003	95.15	402227	bb	Unknown

3. NMR spectra of products

(*S*)-2'-amino-2-oxo-6'-phenylspiro[indoline-3,4'-pyran]-3',5'-dicarbonitrile (**Q4a**):

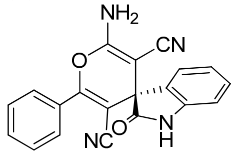

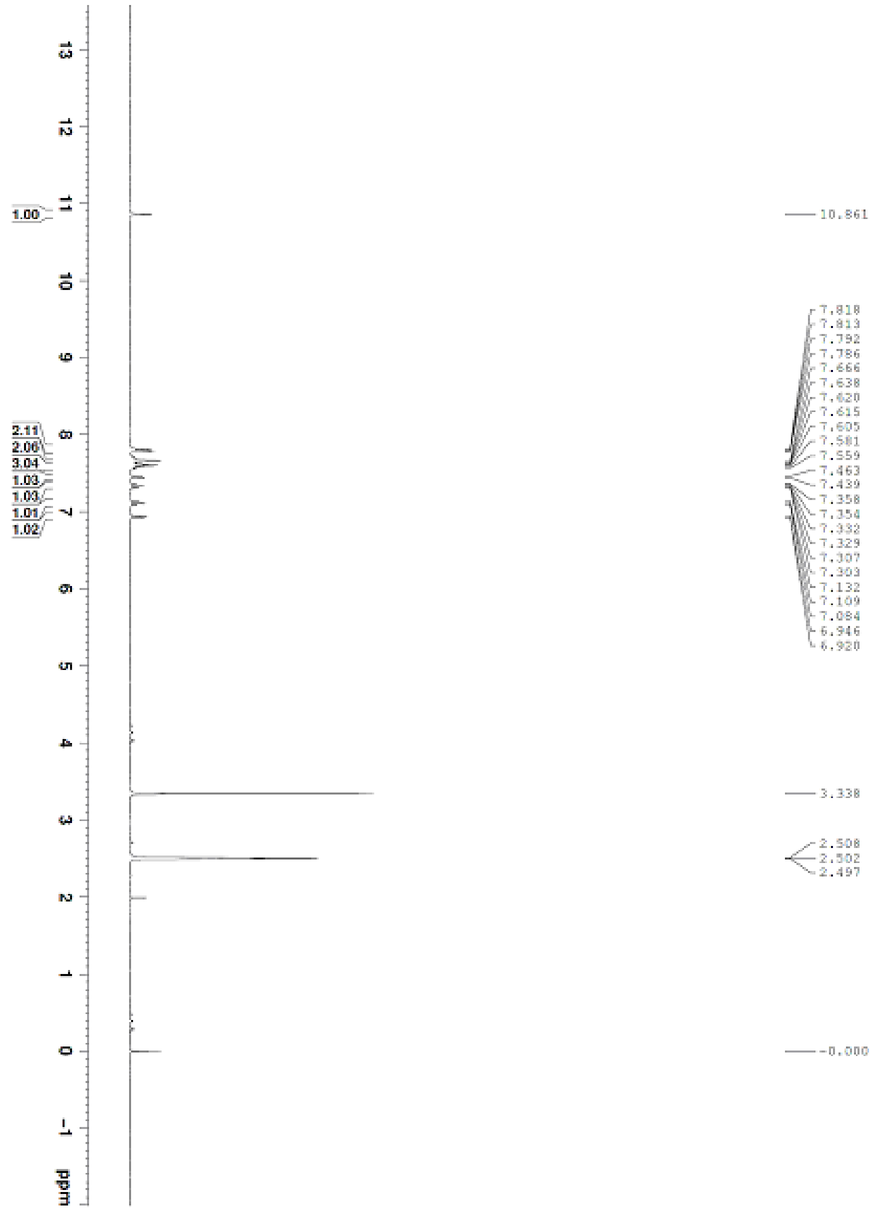

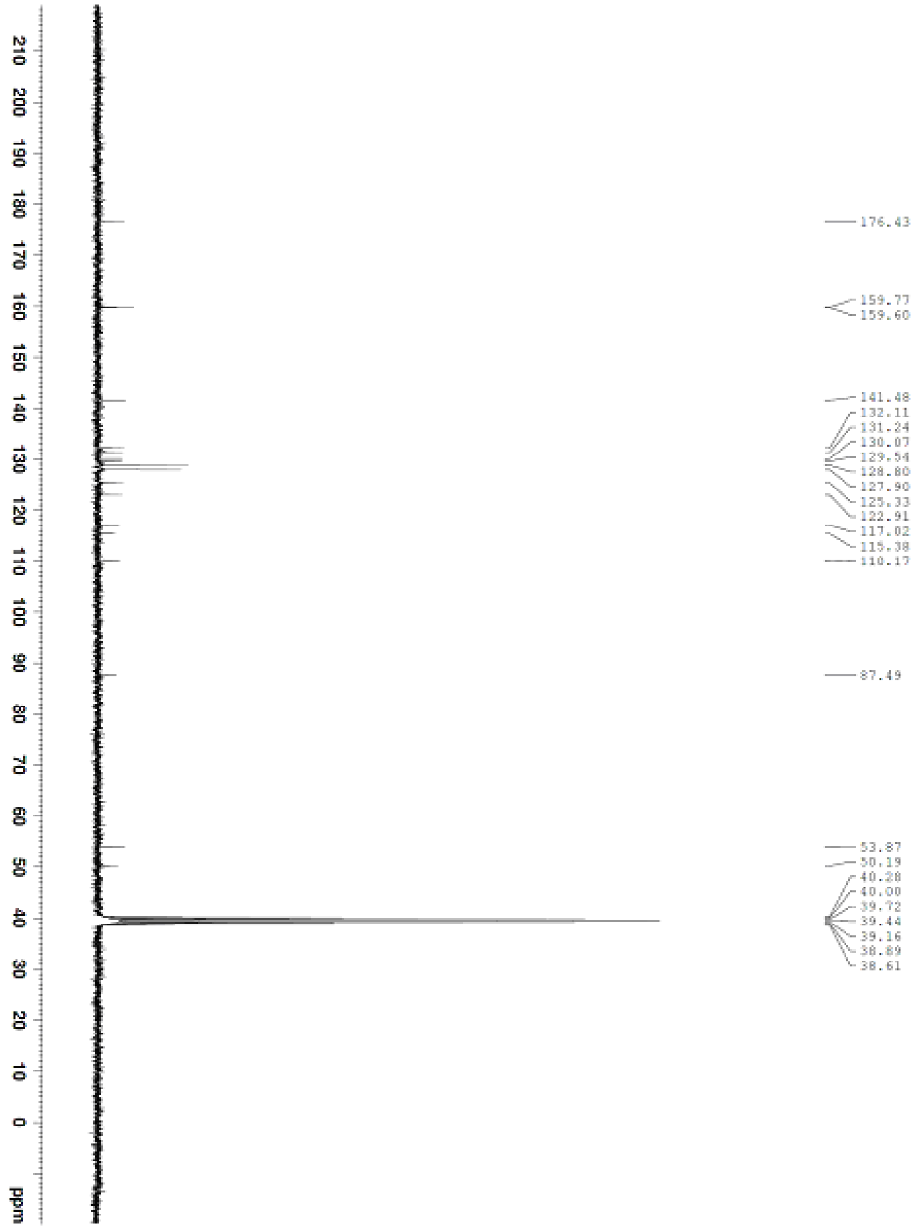

176.43
159.77
159.60
141.48
132.11
131.24
130.07
129.54
128.80
127.90
125.33
122.91
117.02
115.38
110.17
87.49
53.87
50.19
40.28
40.00
39.72
39.44
39.16
38.89
38.61
ppm

(*S*)-2'-amino-5-fluoro-2-oxo-6'-phenylspiro[indoline-3,4'-pyran]-3',5'-dicarbonitrile (**Q4b**)

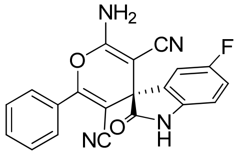

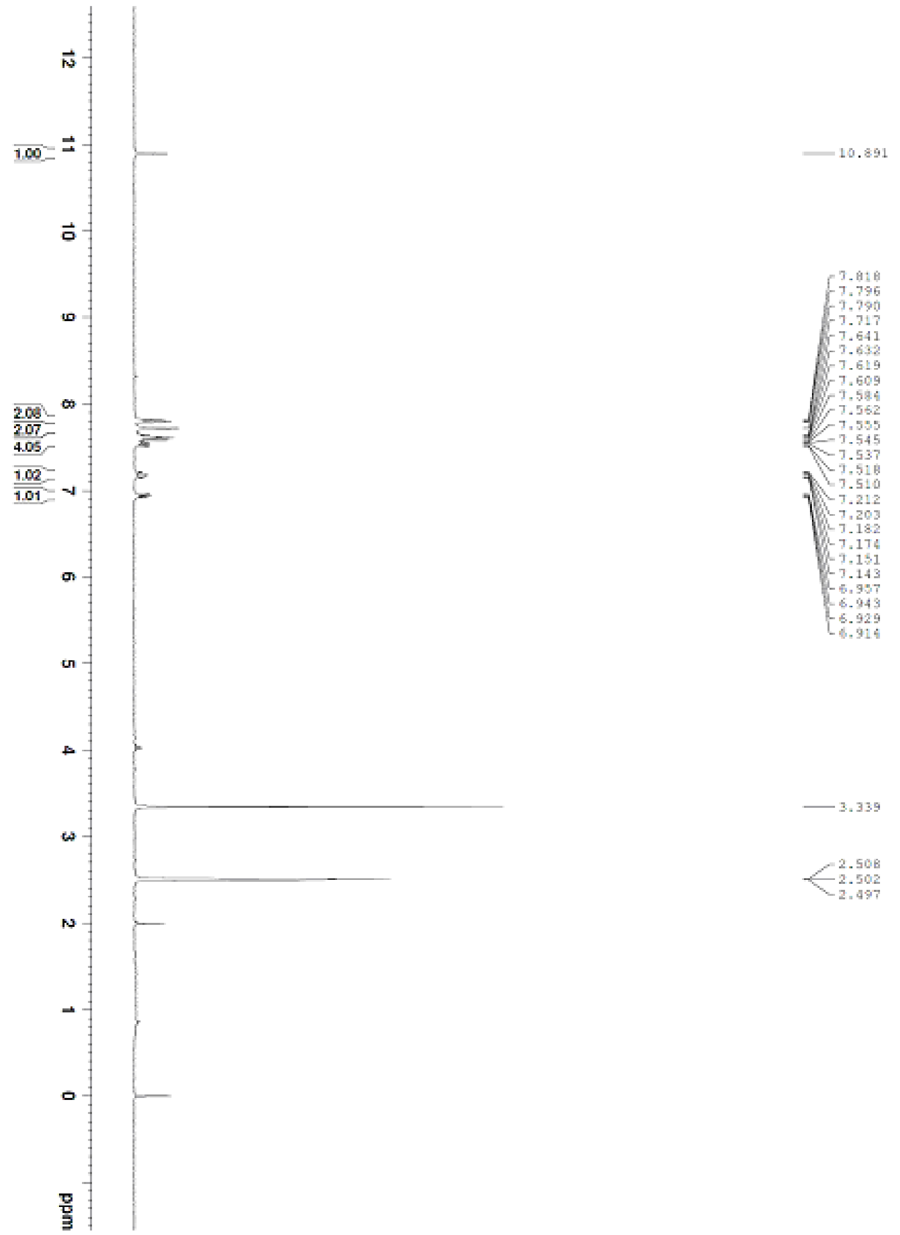

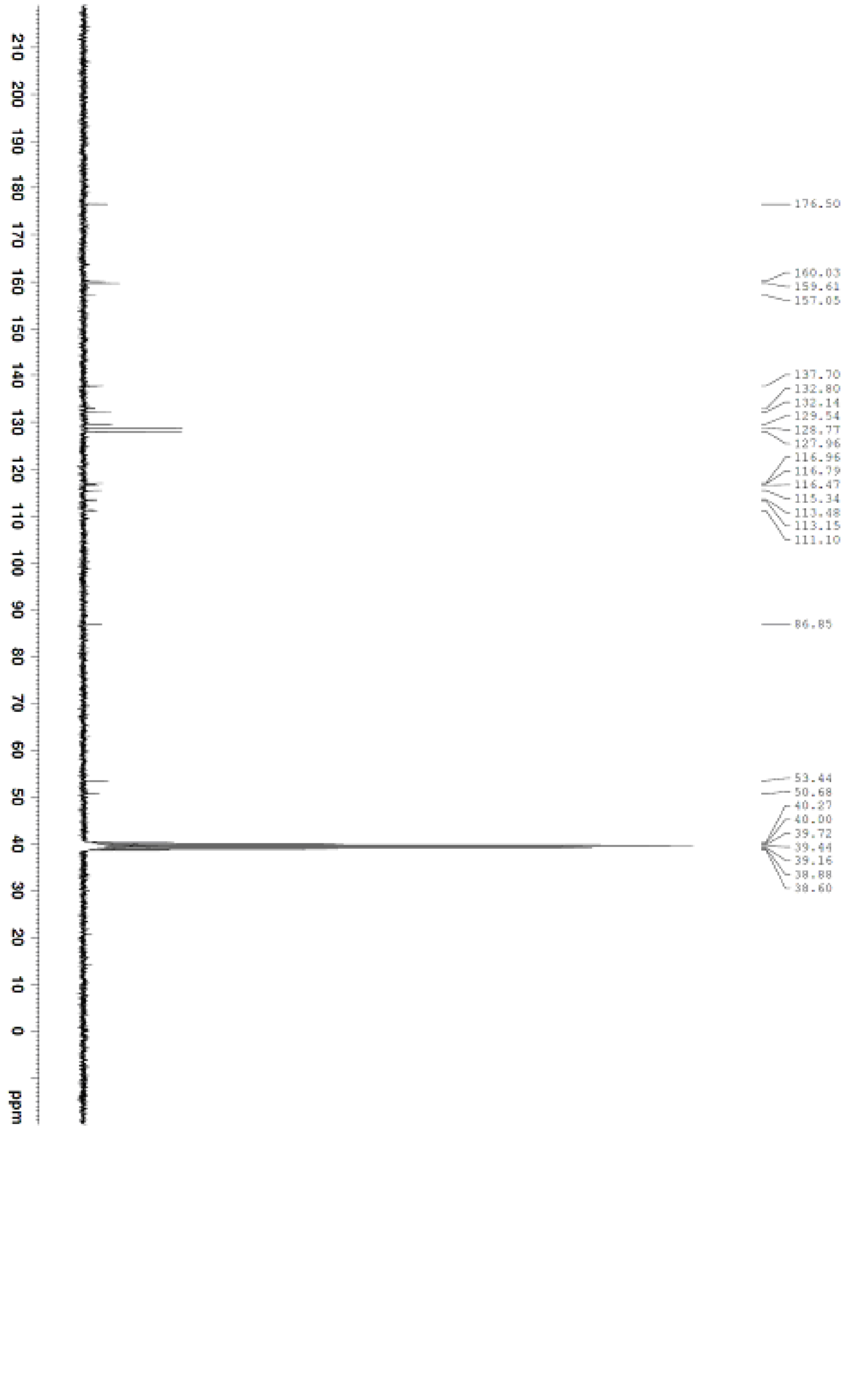
176.50
160.03
159.61
157.05
137.70
132.80
132.14
129.54
128.77
127.96
116.96
116.79
116.47
115.34
113.48
113.15
111.10
86.85
53.44
50.68
40.27
40.00
39.72
39.44
39.16
38.88
38.60
ppm

(*S*)-2'-amino-7-fluoro-2-oxo-6'-phenylspiro[indoline-3,4'-pyran]-3',5'-dicarbonitrile (**Q4c**)

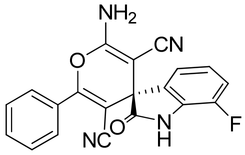

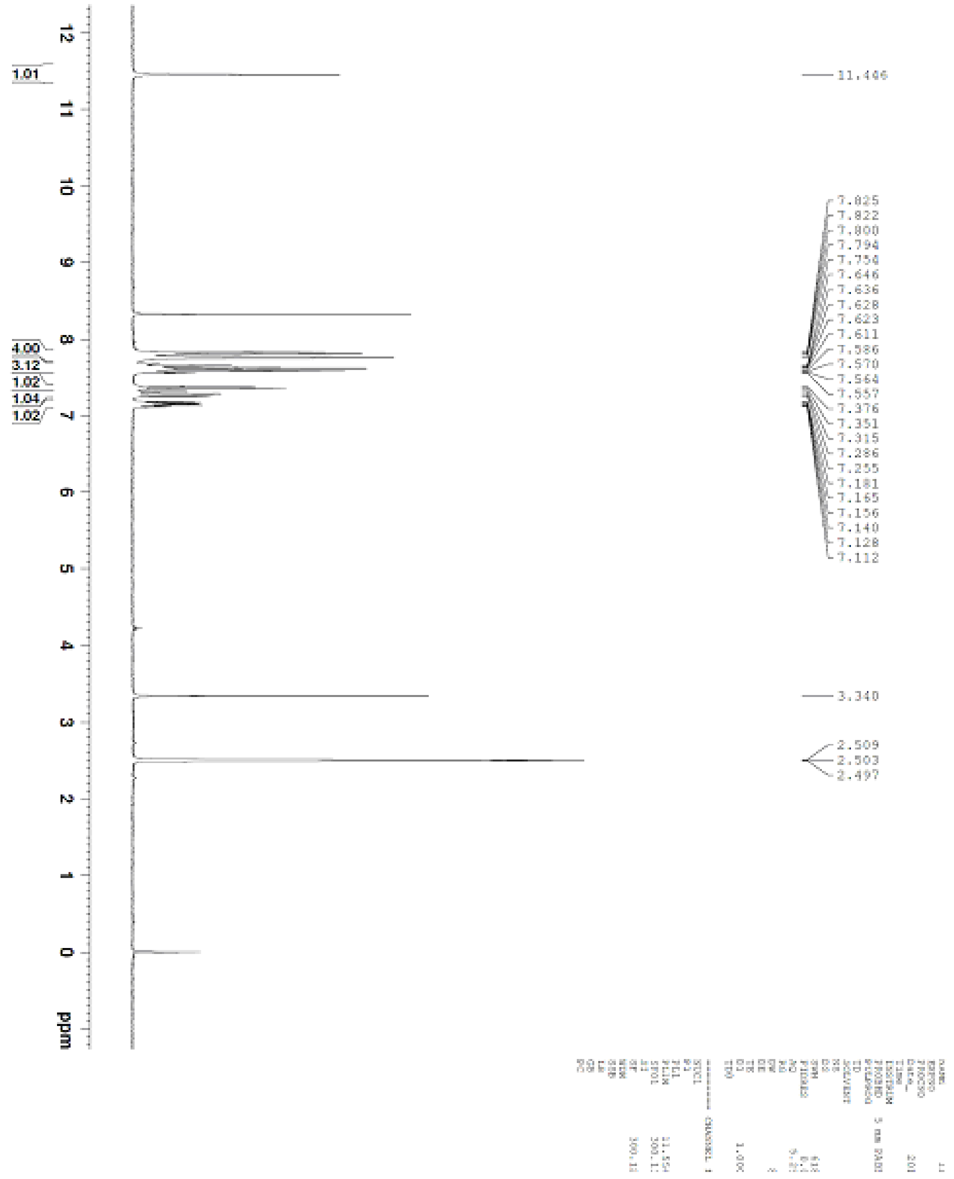

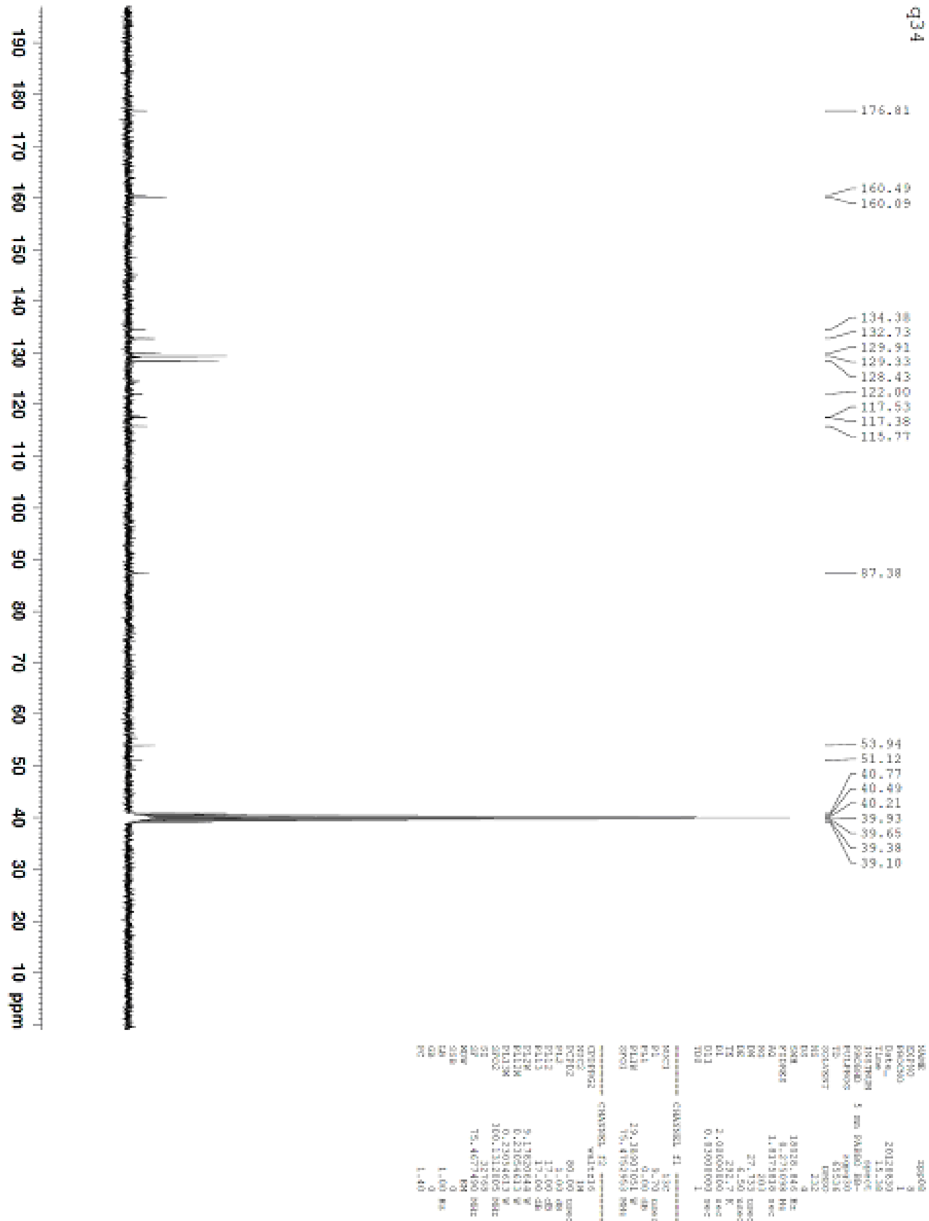
q34
176.81
160.49
160.09
134.38
132.73
129.91
129.33
128.43
122.00
117.53
117.38
115.77
87.38
53.94
51.12
40.77
40.49
40.21
39.93
39.65
39.38
39.10
ppm

(*S*)-2'-amino-5-methoxy-2-oxo-6'-phenylspiro[indoline-3,4'-pyran]-3',5'-dicarbonitrile (**Q4d**)

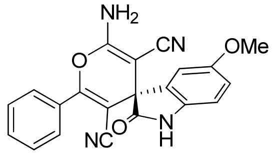

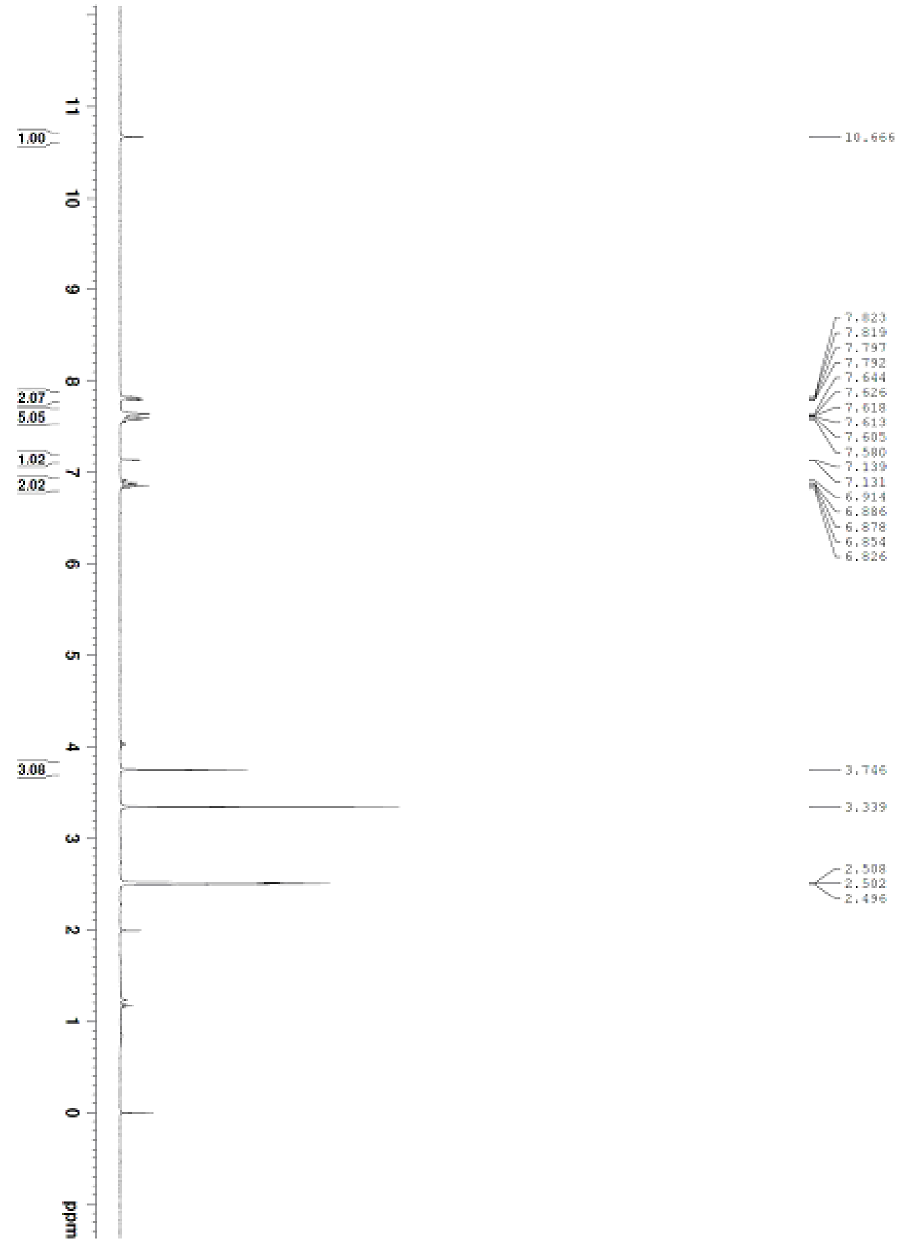

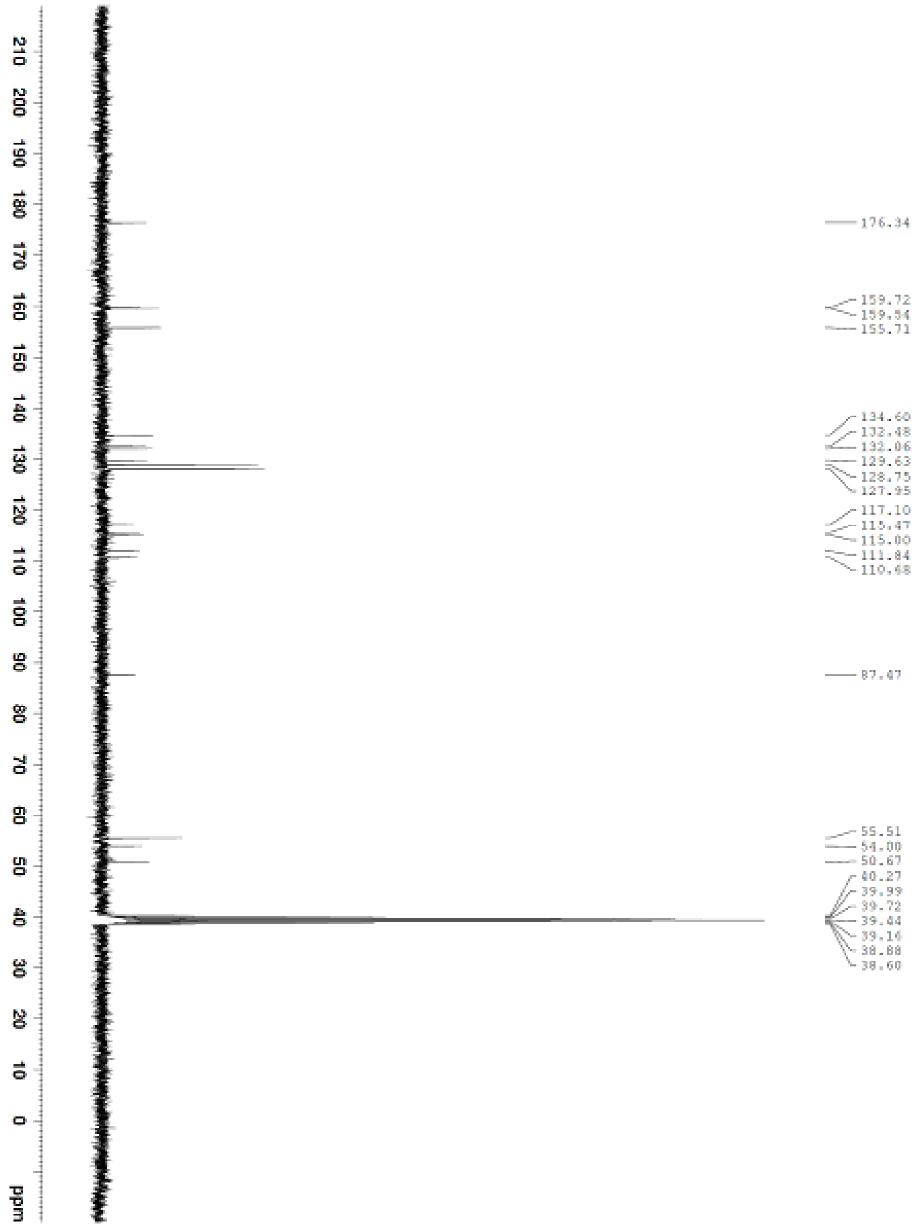
176.34
159.72
159.54
155.71
134.60
132.48
132.06
129.63
128.75
127.95
117.10
115.47
115.00
111.84
110.68
87.87
55.51
54.00
50.67
40.27
39.99
39.72
39.44
39.16
38.88
38.60
ppm

(*S*)-2'-amino-1-methyl-2-oxo-6'-phenylspiro[indoline-3,4'-pyran]-3',5'-dicarbonitrile (**Q4e**)

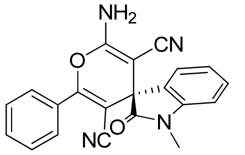

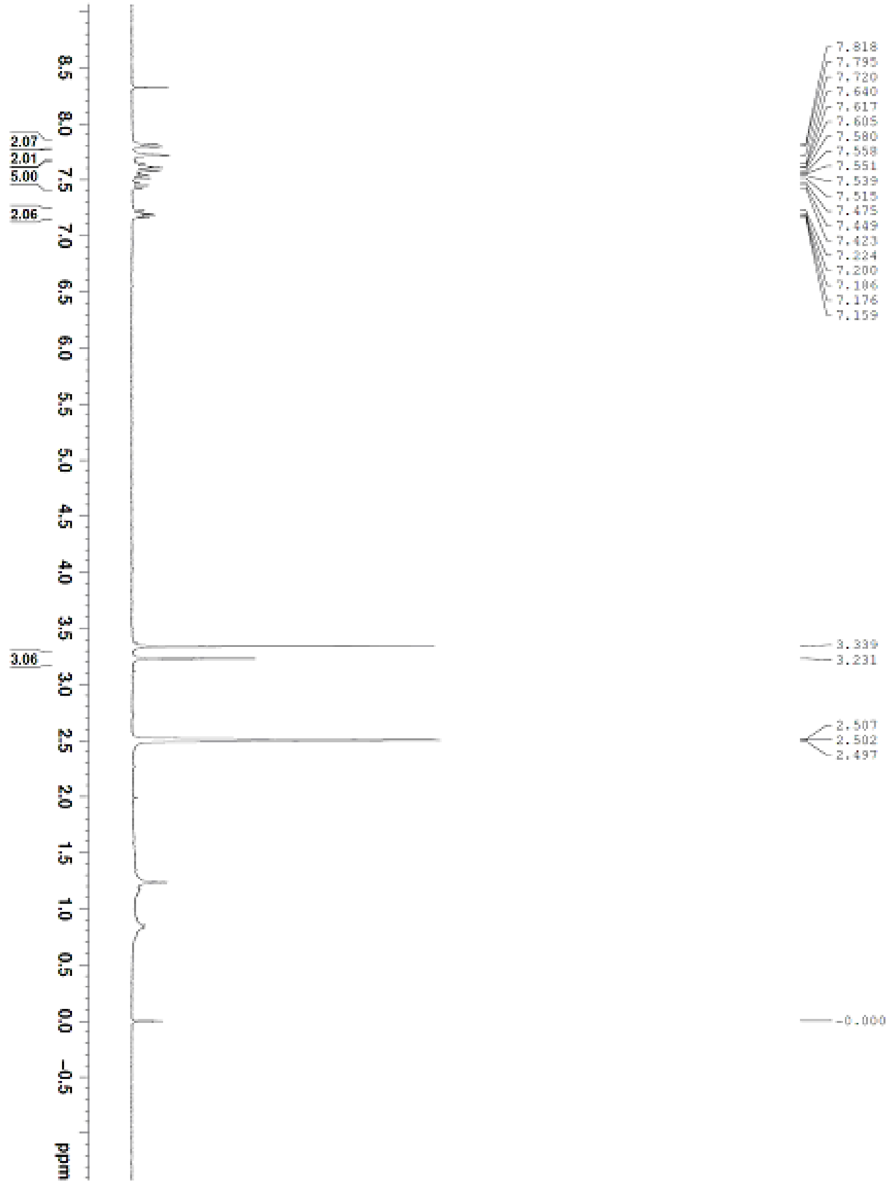

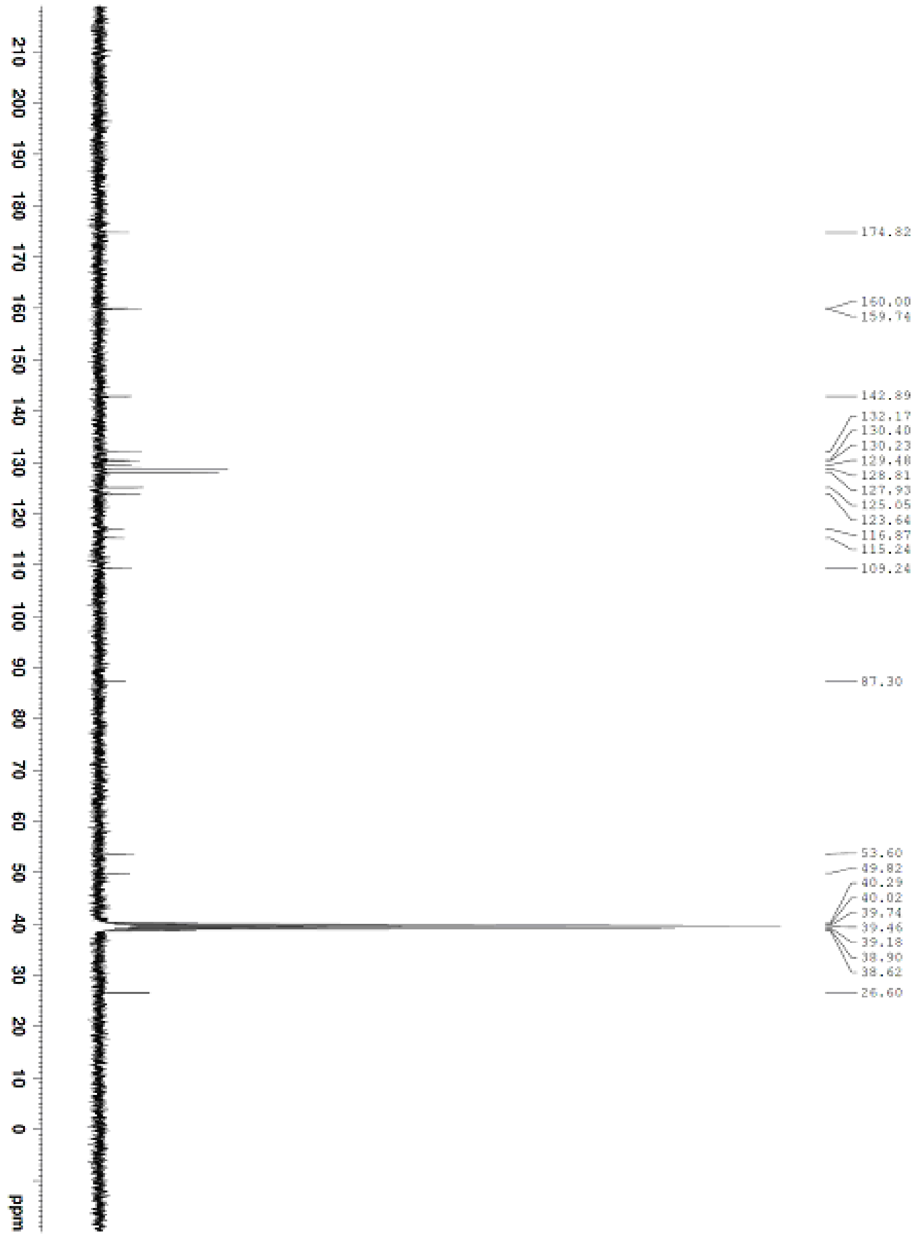
174.82
160.00
159.74
142.89
132.17
130.40
130.23
129.48
128.81
127.93
125.05
123.64
116.87
115.24
109.24
87.30
53.60
49.82
40.29
40.02
39.74
39.46
39.18
38.90
38.62
26.60
ppm

(*S*)-1-allyl-2'-amino-2-oxo-6'-phenylspiro[indoline-3,4'-pyran]-3',5'-dicarbonitrile (**Q4f**)

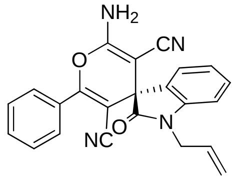

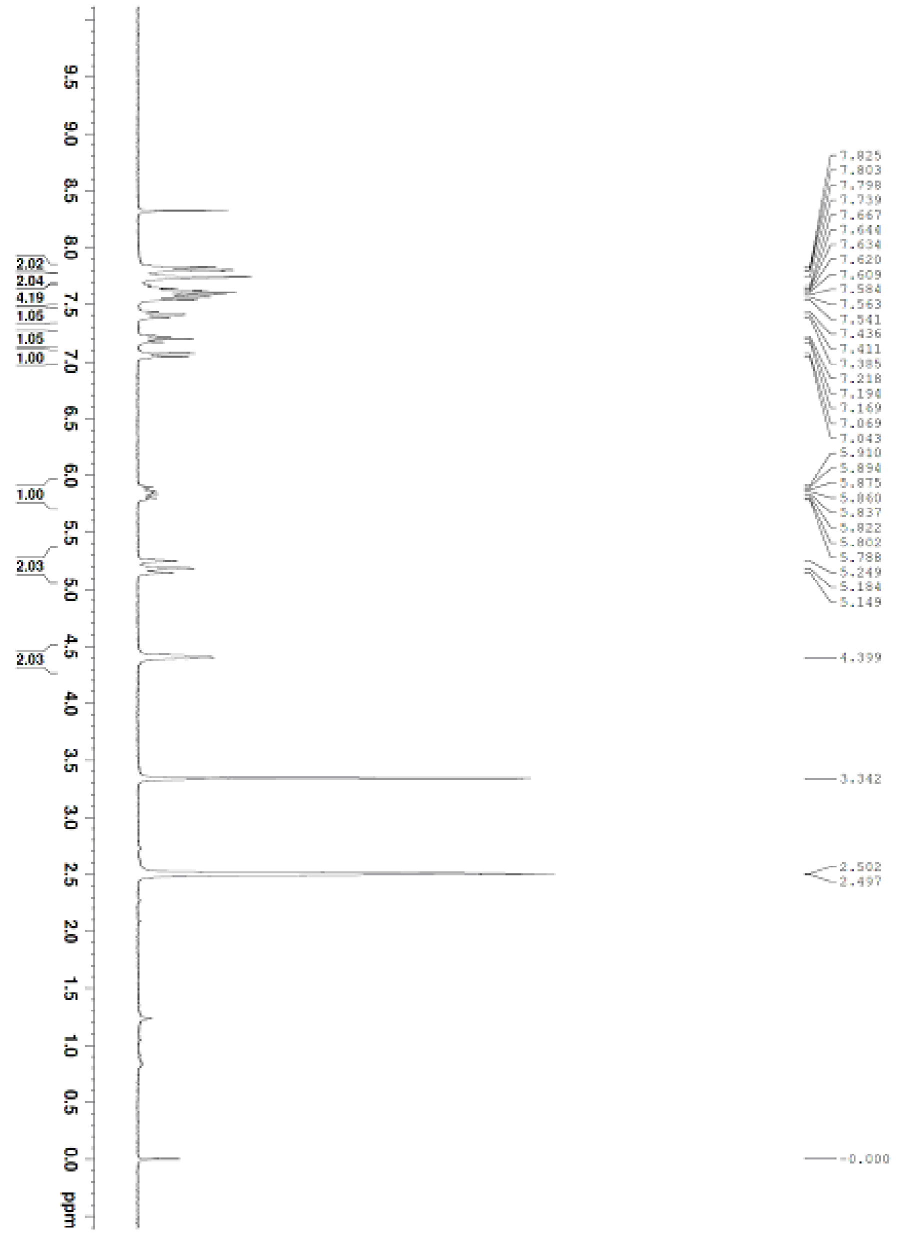

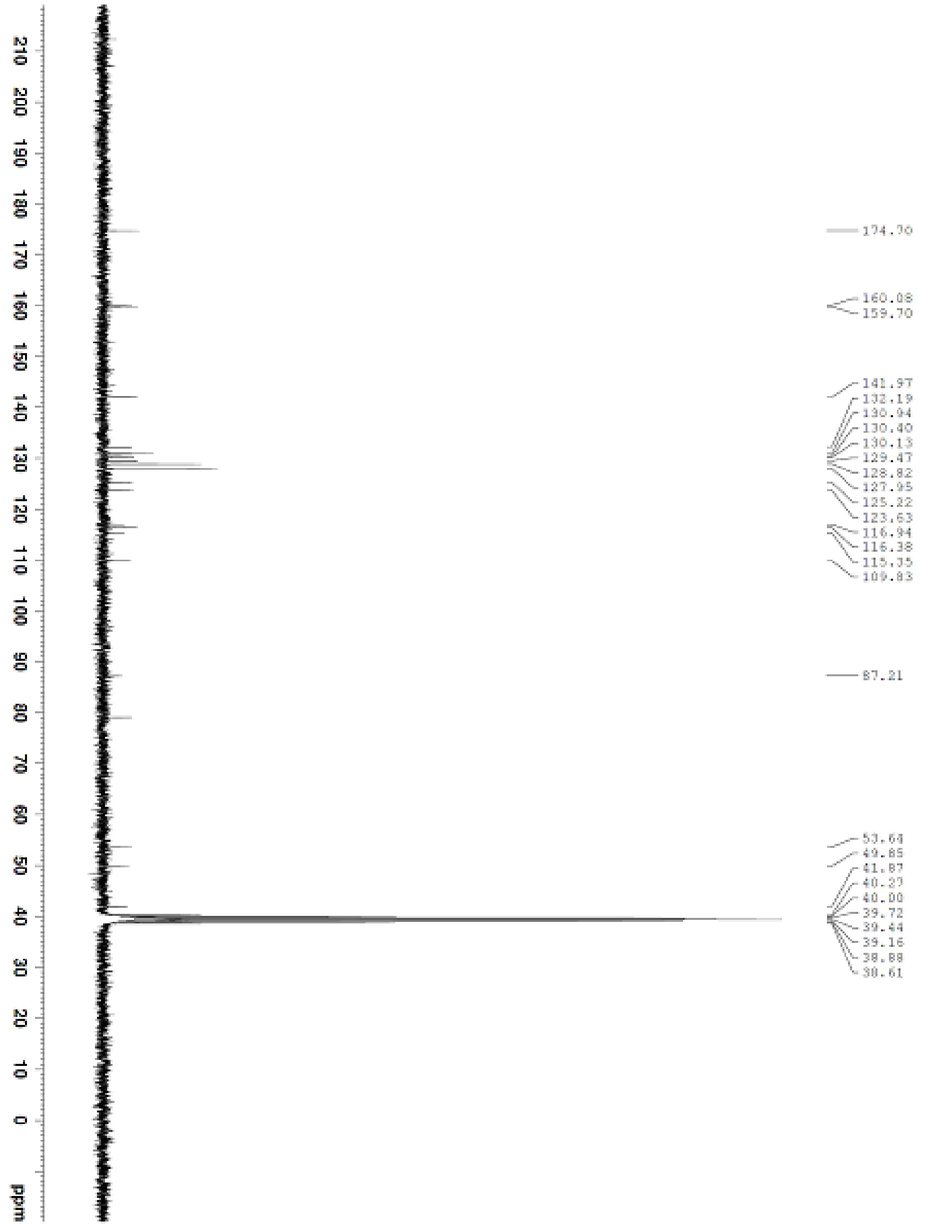
174.70
160.08
159.70
141.97
132.19
130.94
130.40
130.13
129.47
128.82
127.95
125.22
123.63
116.94
116.38
115.35
109.83
87.21
53.64
49.85
41.87
40.27
40.00
39.72
39.44
39.16
38.88
38.61
ppm

(*S*)-2'-amino-2-oxo-1,6'-diphenylspiro[indoline-3,4'-pyran]-3',5'-dicarbonitrile (**Q4g**)

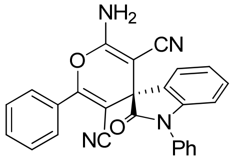

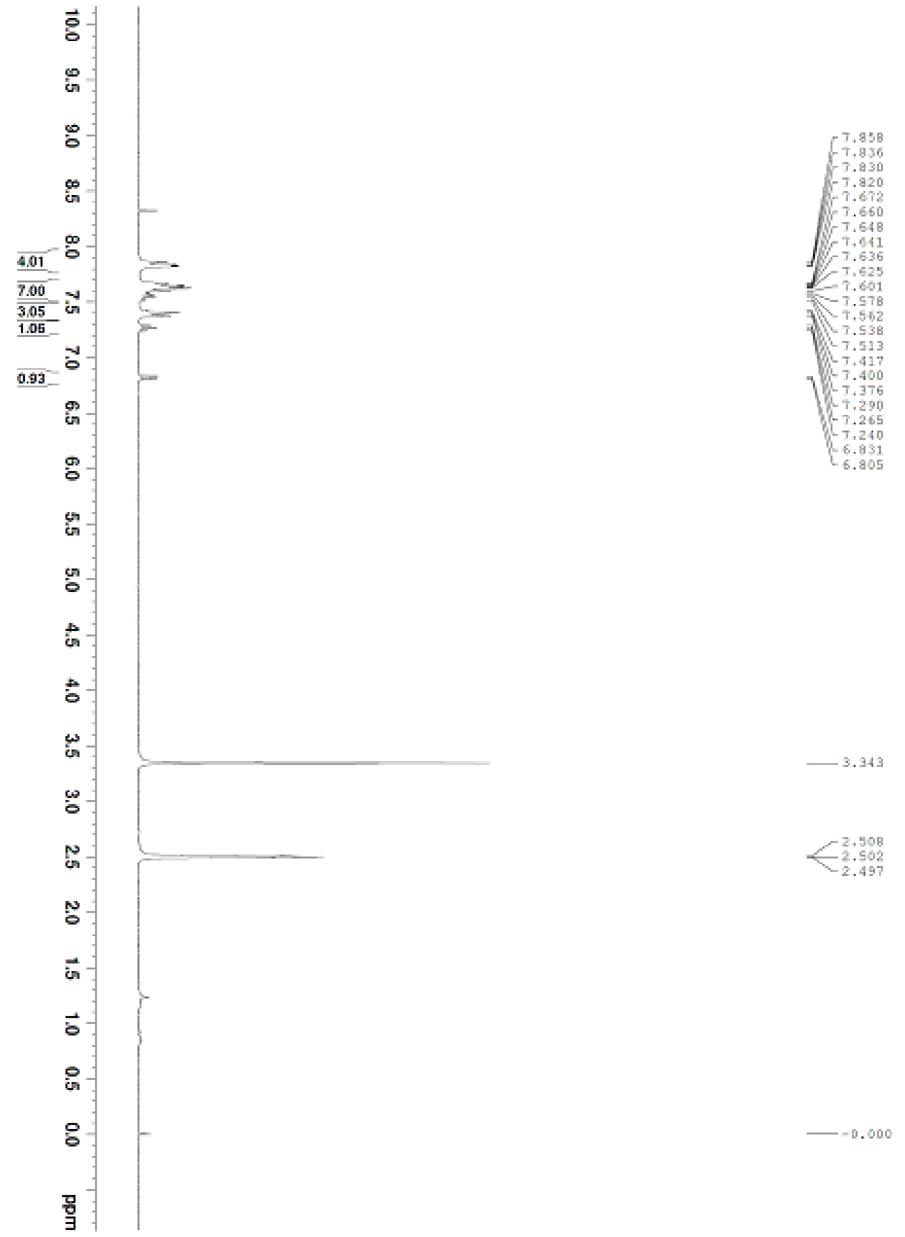

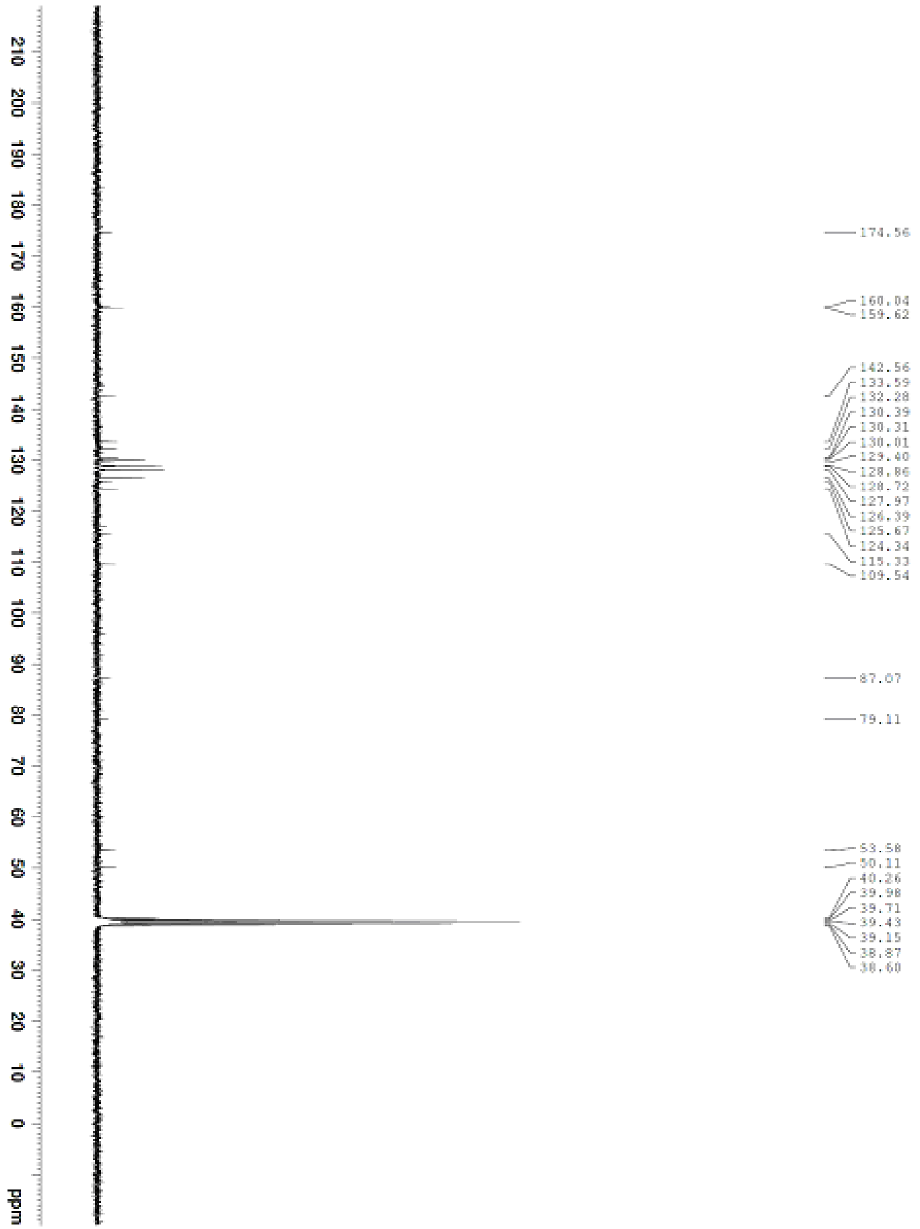
174.56
160.04
159.62
142.56
133.59
132.28
130.39
130.31
130.01
129.40
128.86
128.72
127.97
126.39
125.67
124.34
115.33
109.54
87.07
79.11
53.58
50.11
40.26
39.98
39.71
39.43
39.15
38.87
38.60
ppm

(*S*)-2'-amino-1-benzyl-2-oxo-6'-phenylspiro[indoline-3,4'-pyran]-3',5'-dicarbonitrile (**Q4h**)

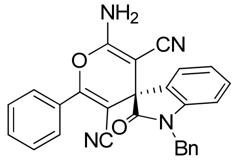

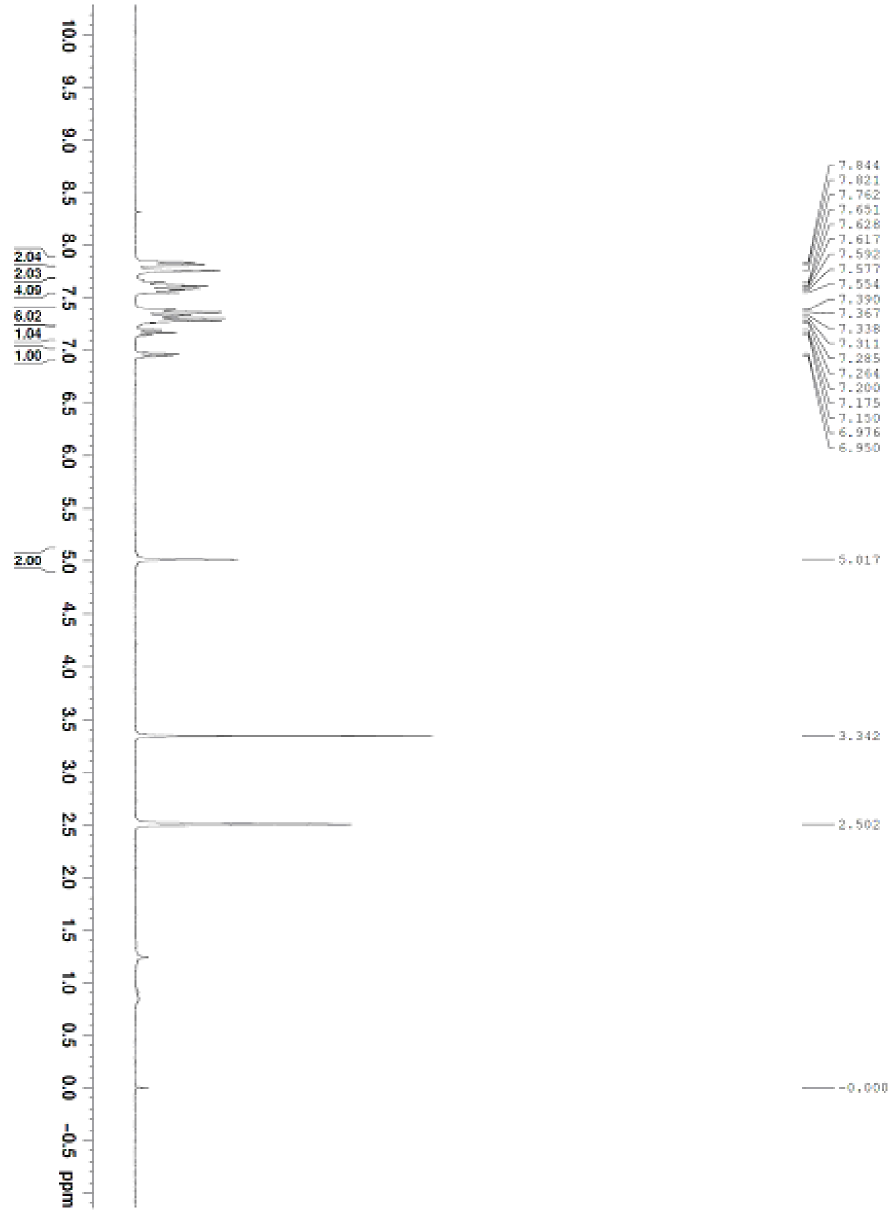

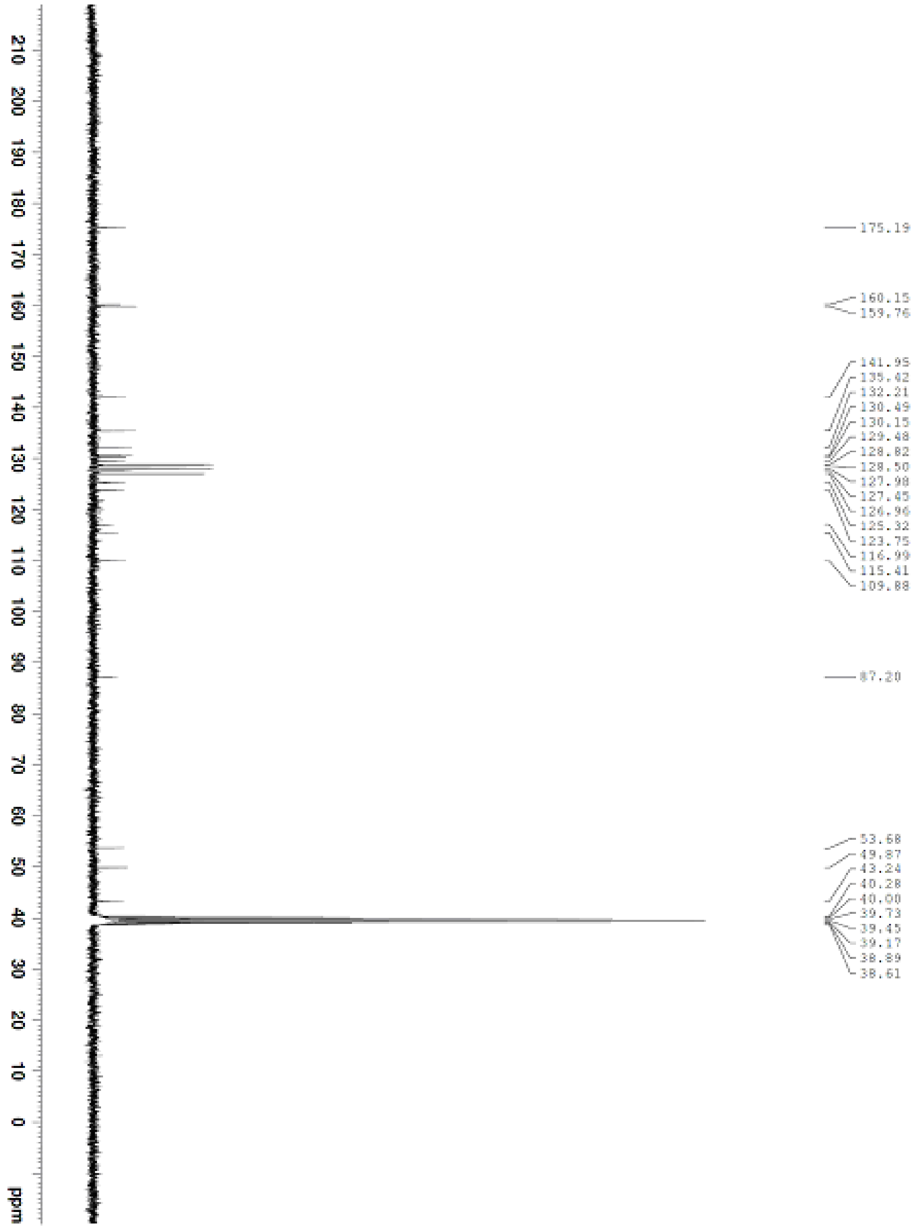
175.19
160.15
159.76
141.95
135.42
132.21
130.49
130.15
129.48
128.82
128.50
127.98
127.45
126.96
125.32
123.75
116.99
115.41
109.88
87.20
53.68
49.87
43.24
40.28
40.00
39.73
39.45
39.17
38.89
38.61
210
200
190
180
170
160
150
140
130
120
110
100
90
80
70
60
50
40
30
20
10
0
ppm

(*S*)-1-acetyl-2'-amino-2-oxo-6'-phenylspiro[indoline-3,4'-pyran]-3',5'-dicarbonitrile (**Q4i**)

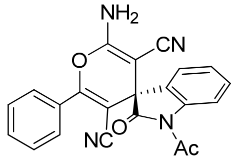

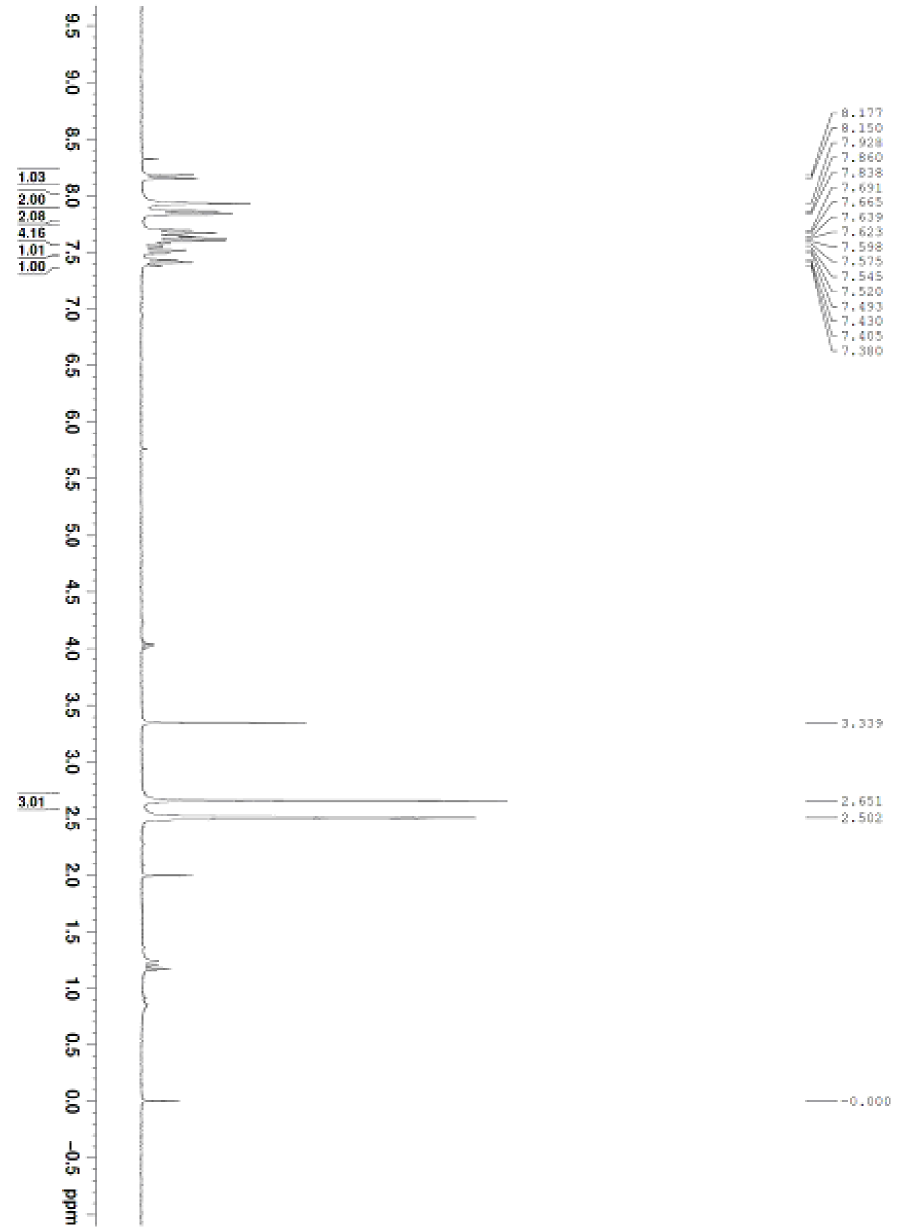

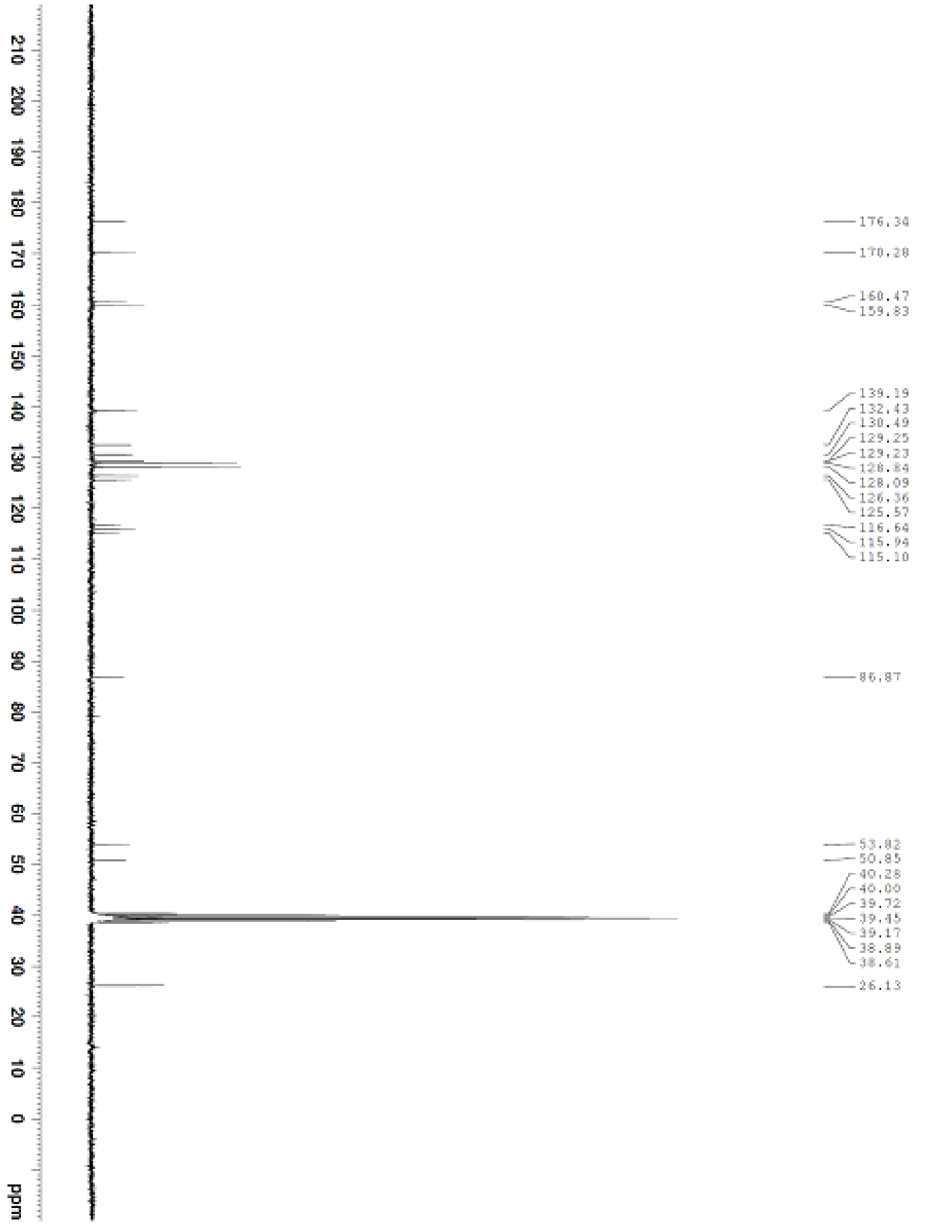
176.34
170.28
160.47
159.83
139.19
132.43
130.49
129.25
129.23
128.84
128.09
126.36
125.57
116.64
115.94
115.10
86.87
53.82
50.85
40.28
40.00
39.72
39.45
39.17
38.89
38.61
26.13
ppm

(*S*)-1-allyl-2'-amino-5-chloro-2-oxo-6'-phenylspiro[indoline-3,4'-pyran]-3',5'-dicarbonitrile (**Q4k**)

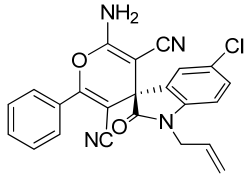

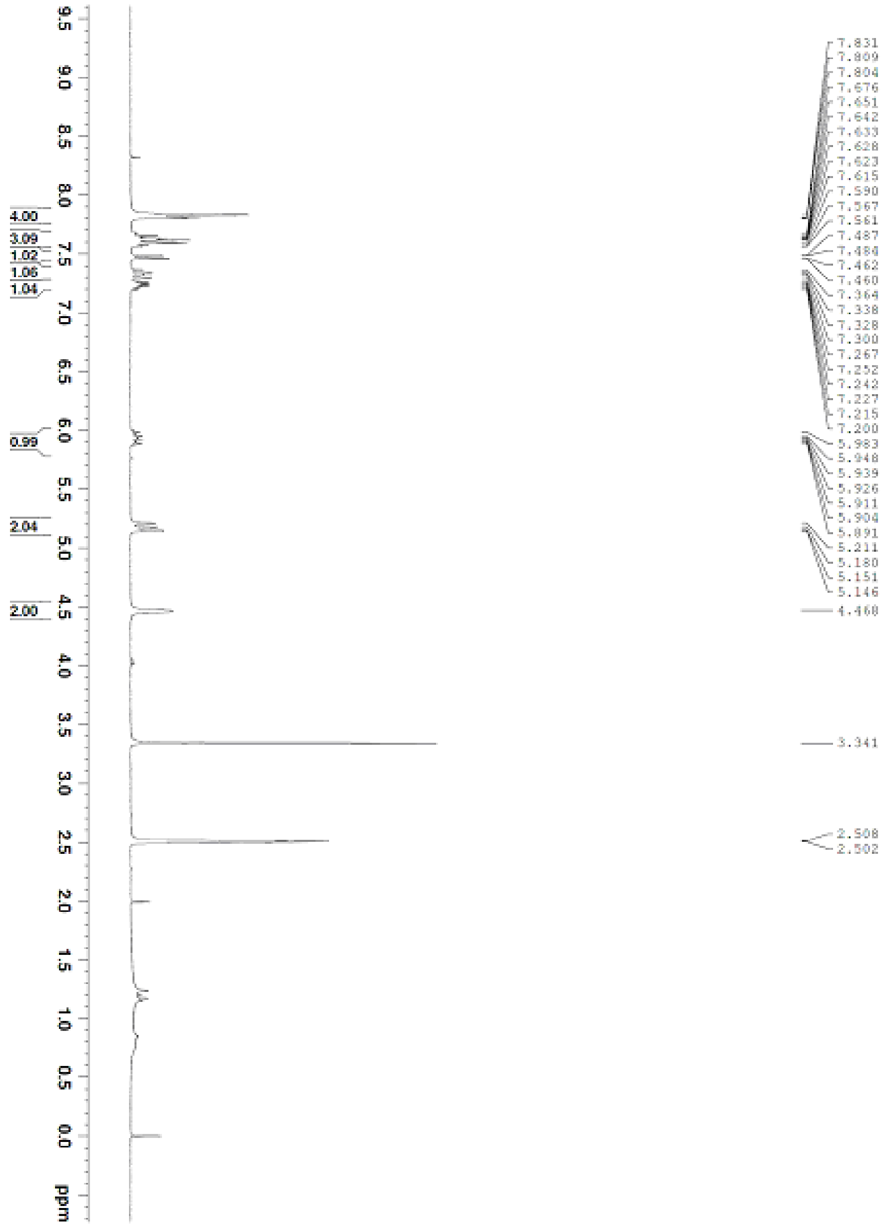

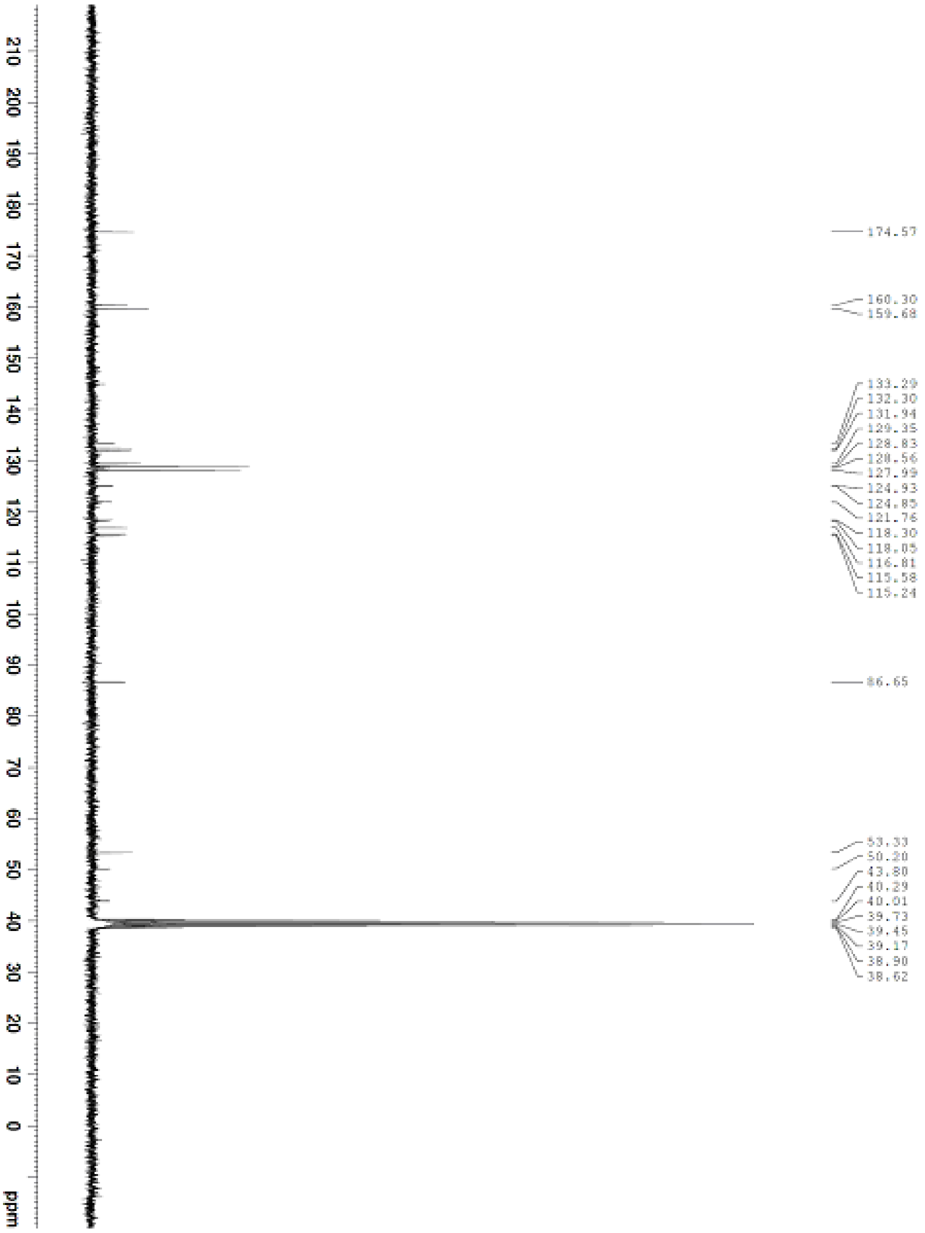
174.57
160.30
159.68
133.29
132.30
131.94
129.35
128.83
128.56
127.99
124.93
124.85
121.76
118.30
118.05
116.81
115.58
115.24
86.65
53.33
50.20
43.80
40.29
40.01
39.73
39.45
39.17
38.90
38.62
ppm

(*S*)-1-allyl-2'-amino-7-chloro-2-oxo-6'-phenylspiro[indoline-3,4'-pyran]-3',5'-dicarbonitrile (**Q4l**)

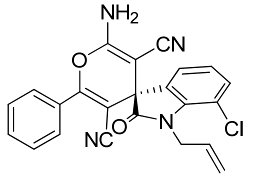

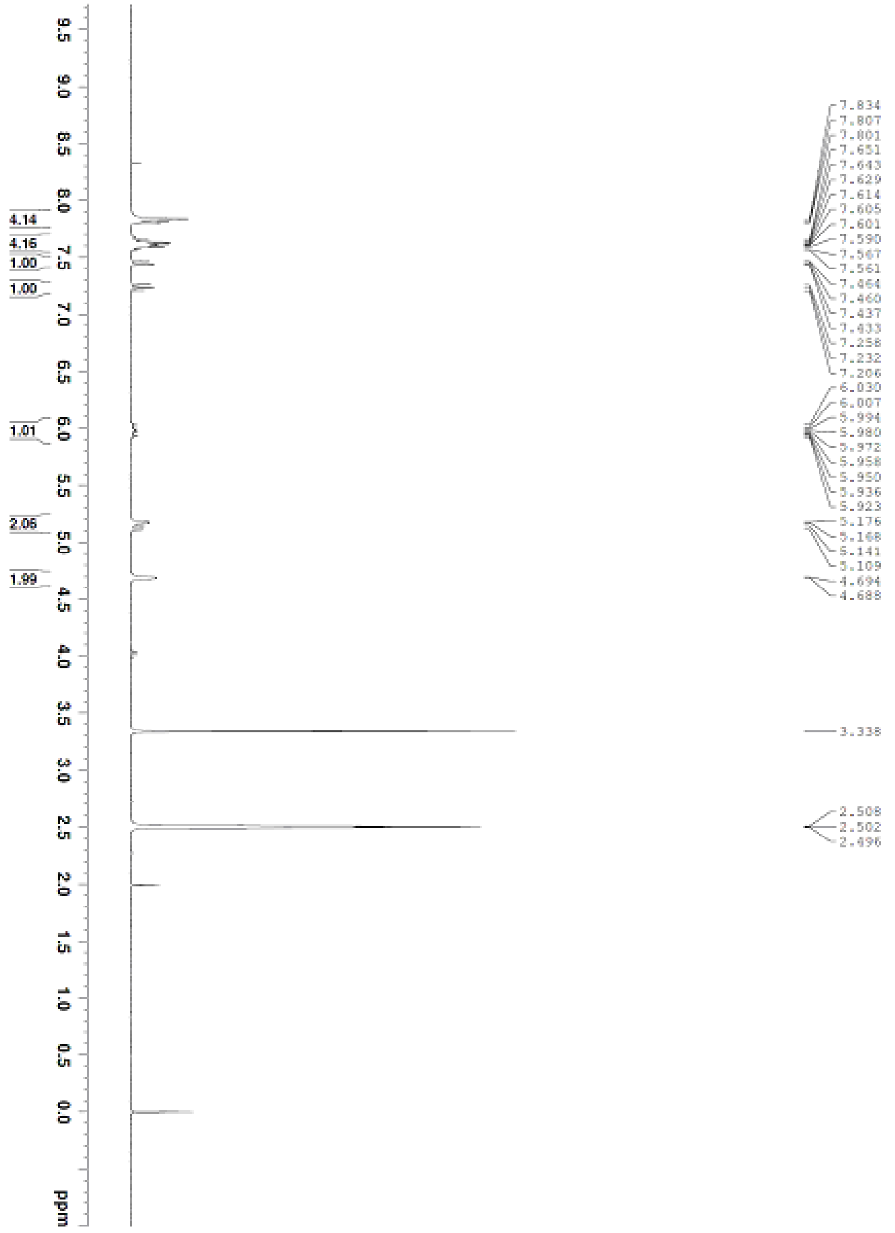

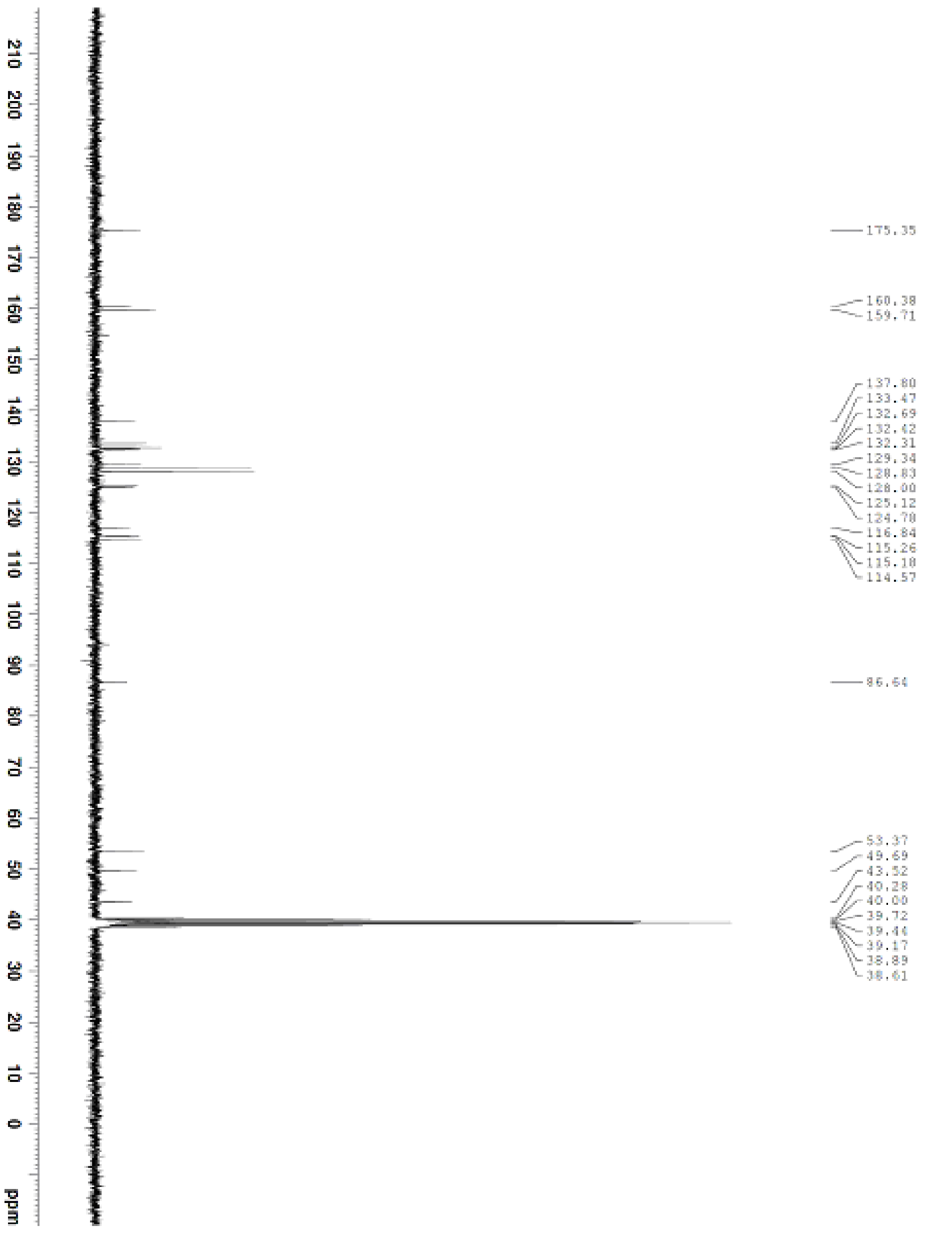
175.35
160.38
159.71
137.80
133.47
132.69
132.42
132.31
129.34
128.83
128.00
125.12
124.78
116.84
115.26
115.18
114.57
86.64
53.37
49.69
43.52
40.28
40.00
39.72
39.44
39.17
38.89
38.61
ppm

(*S*)-1-allyl-2'-amino-5-chloro-7-methyl-2-oxo-6'-phenylspiro[indoline-3,4'-pyran]-3',5'-dicarbonitrile (**Q4m**)

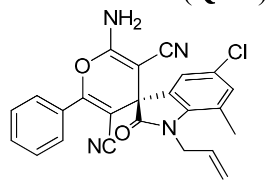

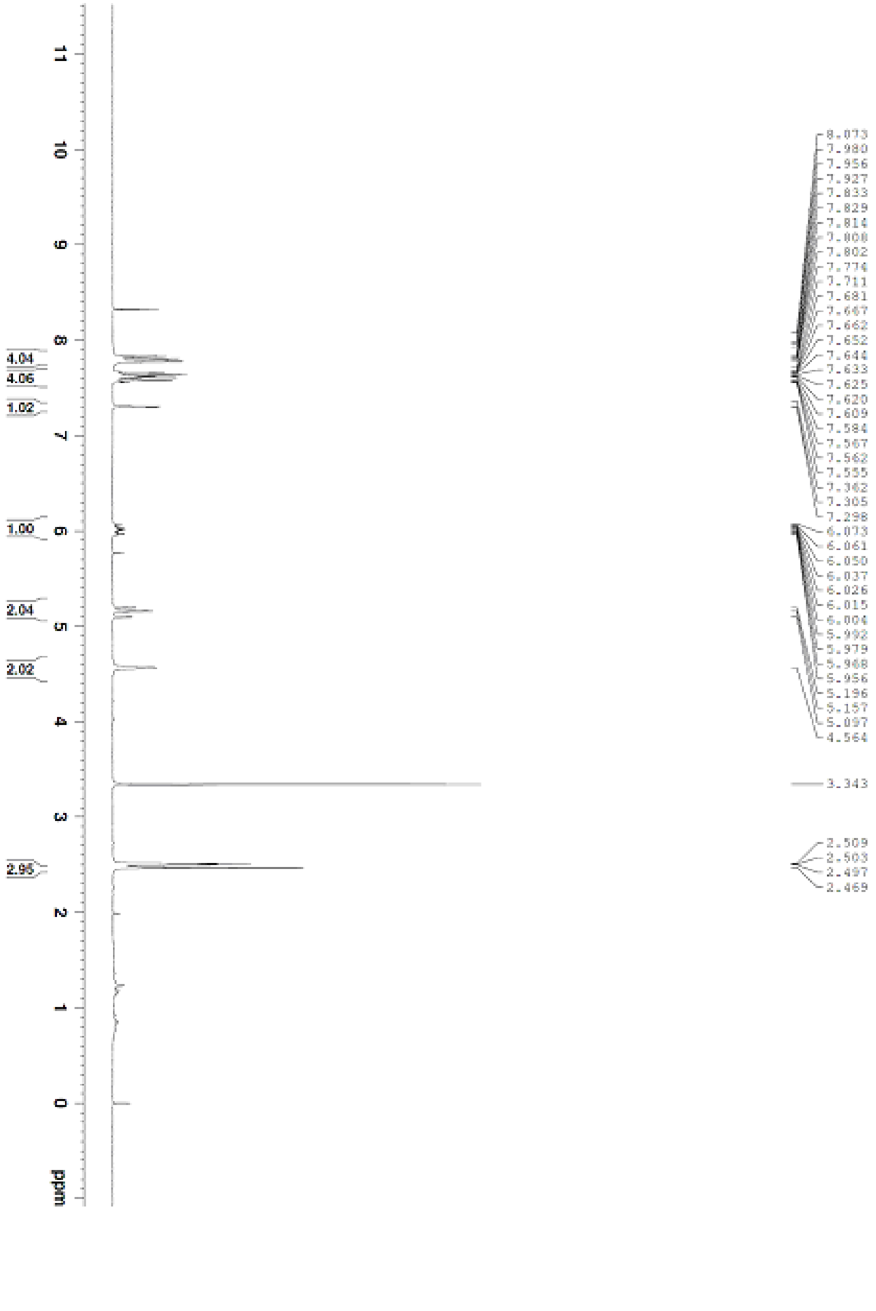

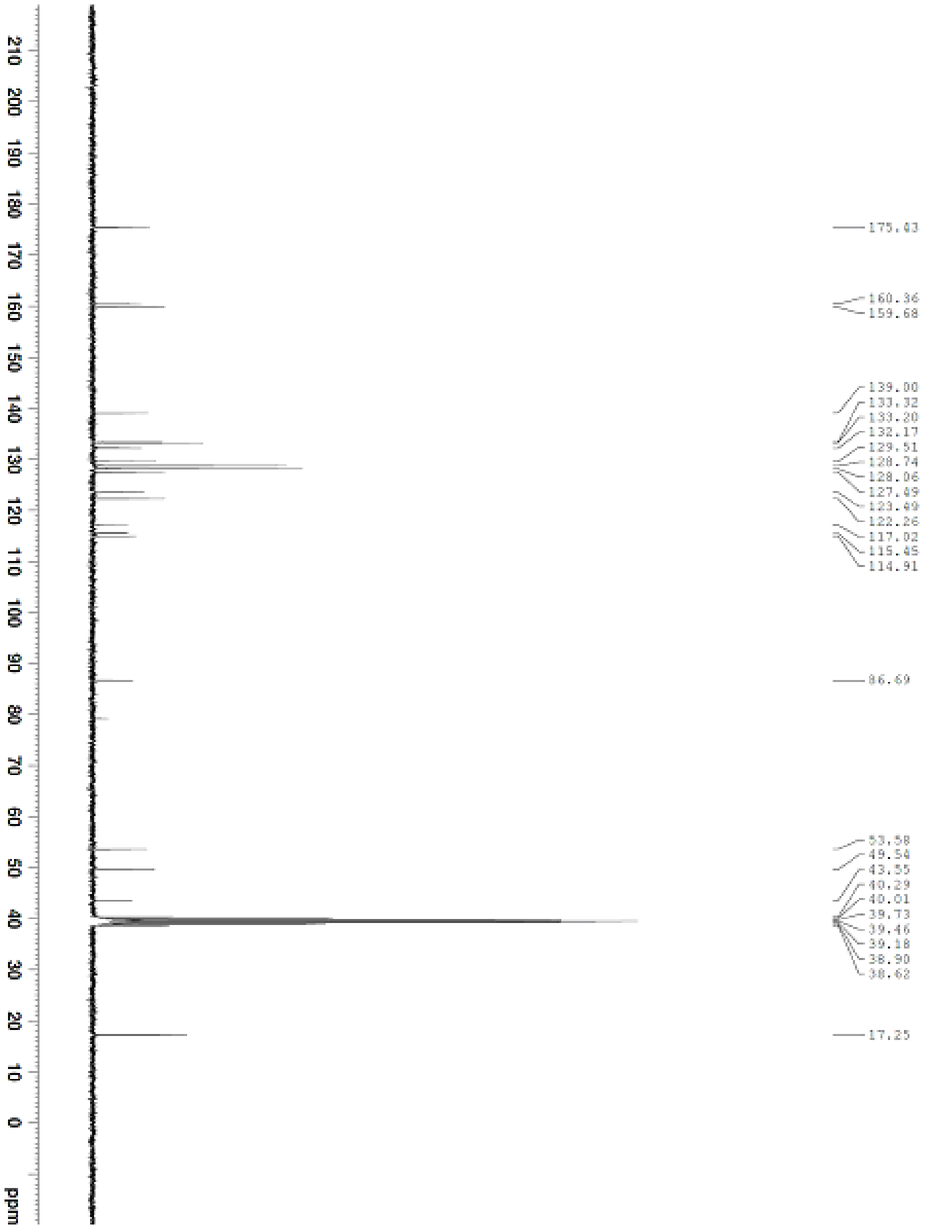
175.43
160.36
159.68
139.00
133.32
133.20
132.17
129.51
128.74
128.06
127.49
123.49
122.26
117.02
115.45
114.91
86.69
53.58
49.54
43.55
40.29
40.01
39.73
39.46
39.18
38.90
38.62
17.25
ppm

(*S*)-1-allyl-2'-amino-5-bromo-2-oxo-6'-phenylspiro[indoline-3,4'-pyran]-3',5'-dicarbonitrile (**Q4n**)

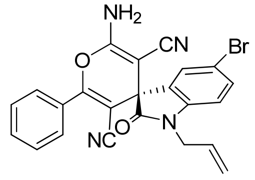

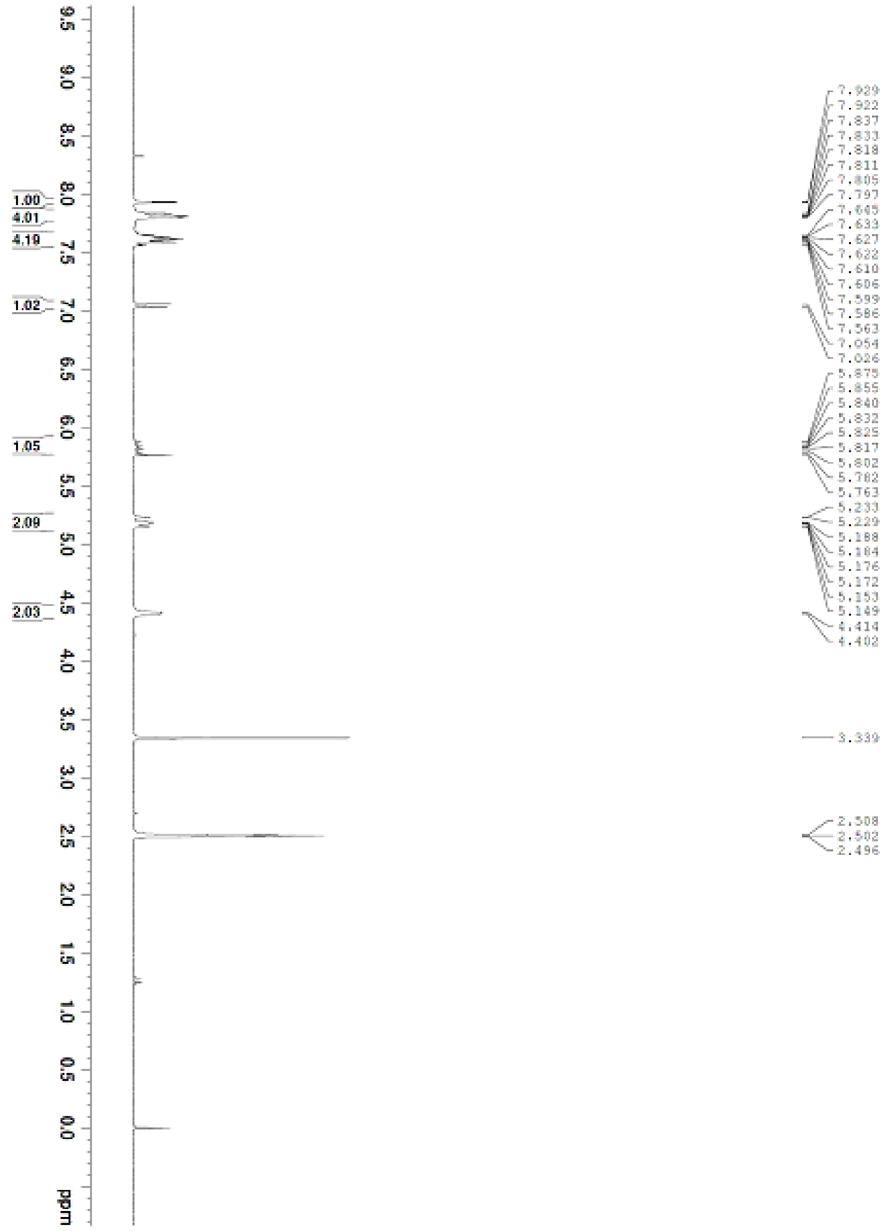

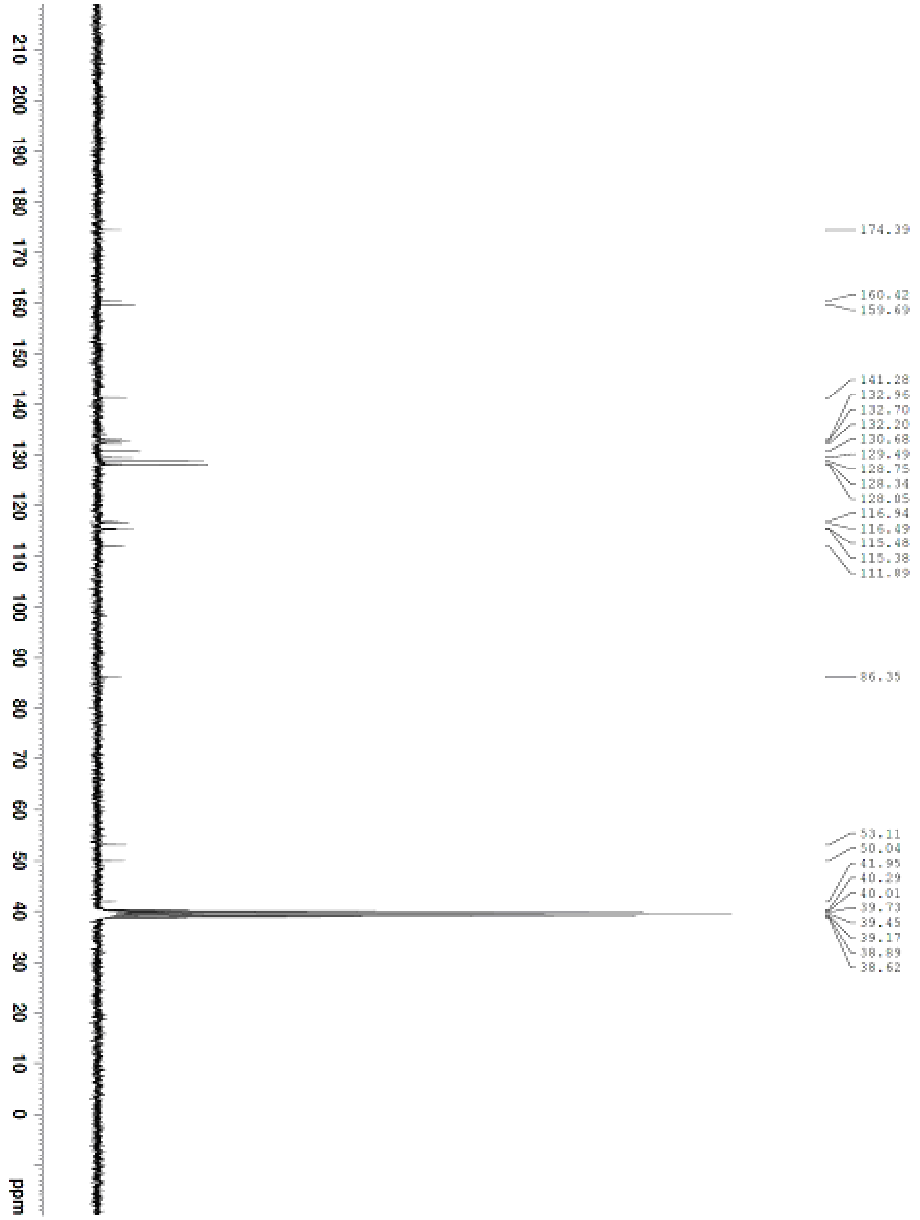
174.39
160.42
159.69
141.28
132.96
132.70
132.20
130.68
129.49
128.75
128.34
128.05
116.94
116.49
115.48
115.38
111.89
86.35
53.11
50.04
41.95
40.29
40.01
39.73
39.45
39.17
38.89
38.62
210 200 190 180 170 160 150 140 130 120 110 100 90 80 70 60 50 40 30 20 10 0 ppm

(*S*)-1-allyl-2'-amino-5-methyl-2-oxo-6'-phenylspiro[indoline-3,4'-pyran]-3',5'-dicarbonitrile (**Q4o**)

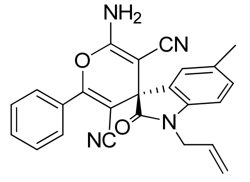

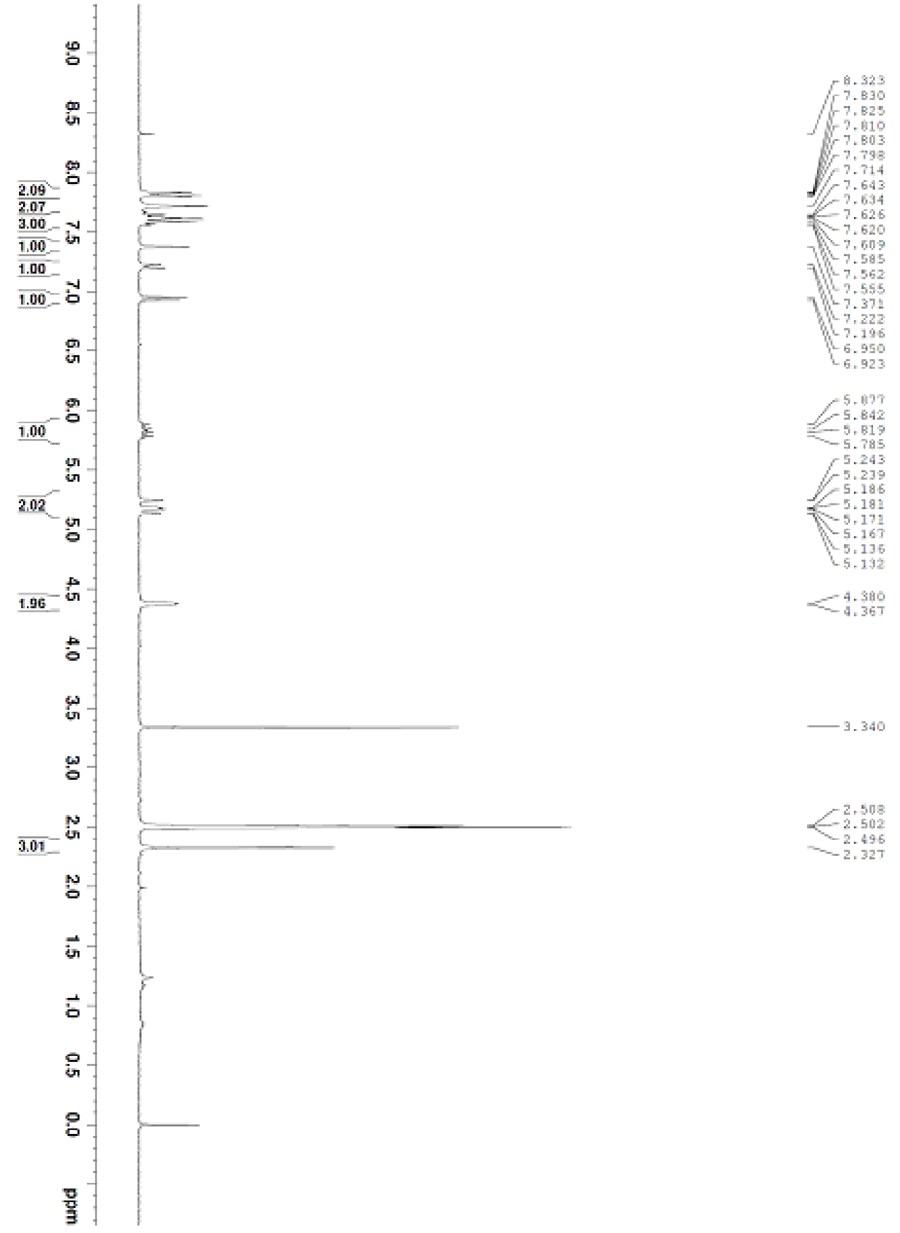

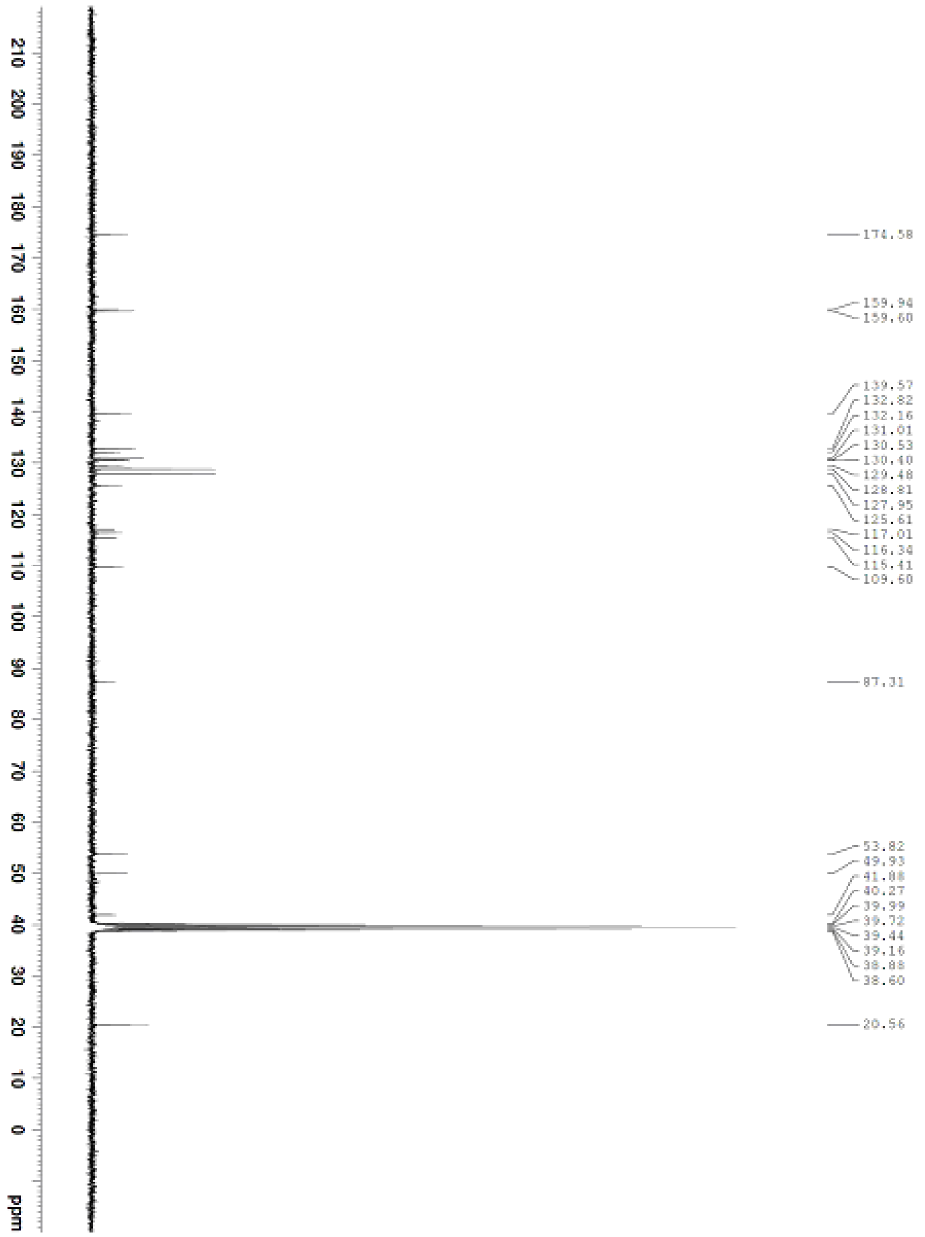
174.58
159.94
159.60
139.57
132.82
132.16
131.01
130.53
130.40
129.48
128.81
127.95
125.61
117.01
116.34
115.41
109.60
87.31
53.82
49.93
41.88
40.27
39.99
39.72
39.44
39.16
38.88
38.60
20.56
ppm

(*S*)-1-allyl-2'-amino-5-methoxy-2-oxo-6'-phenylspiro[indoline-3,4'-pyran]-3',5'-dicarbonitrile (**Q4p**)

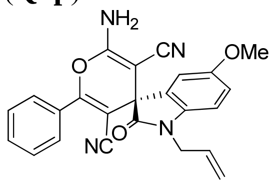

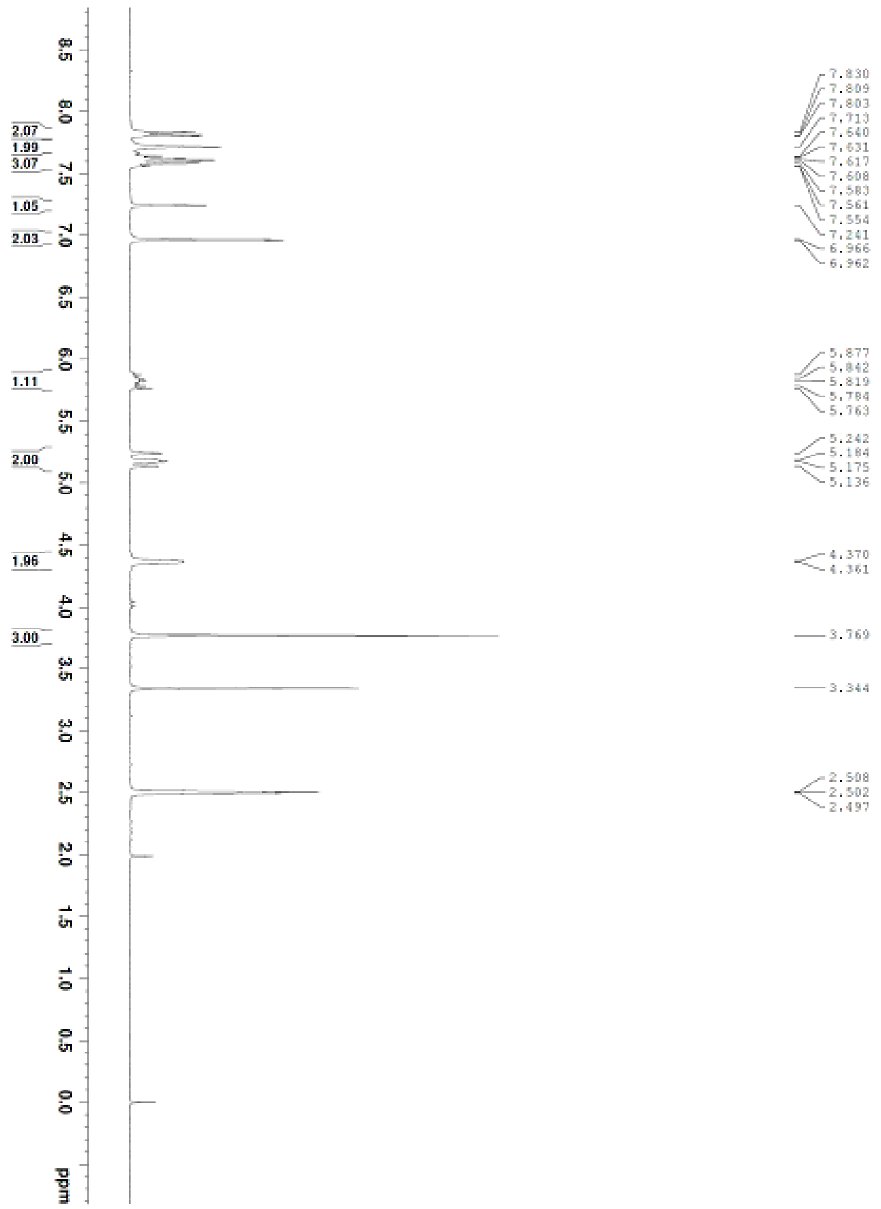

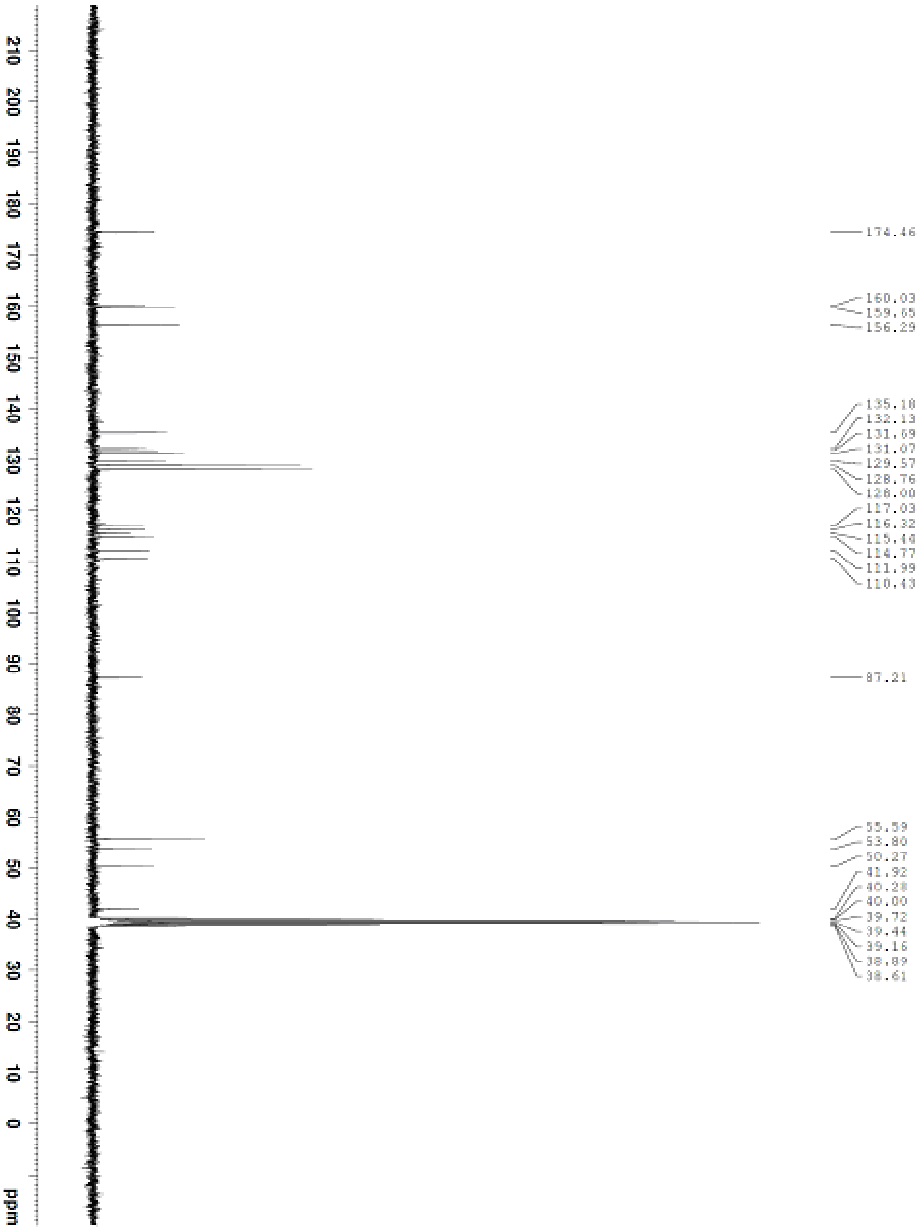
174.46
160.03
159.65
156.29
135.18
132.13
131.69
131.07
129.57
128.76
128.00
117.03
116.32
115.44
114.77
111.99
110.43
87.21
55.59
53.80
50.27
41.92
40.28
40.00
39.72
39.44
39.16
38.89
38.61
210
200
190
180
170
160
150
140
130
120
110
100
90
80
70
60
50
40
30
20
10
0
ppm

(*S*)-2'-amino-6'-(3-chlorophenyl)-2-oxospiro[indoline-3,4'-pyran]-3',5'-dicarbonitrile (**Q4q**)

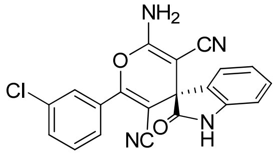

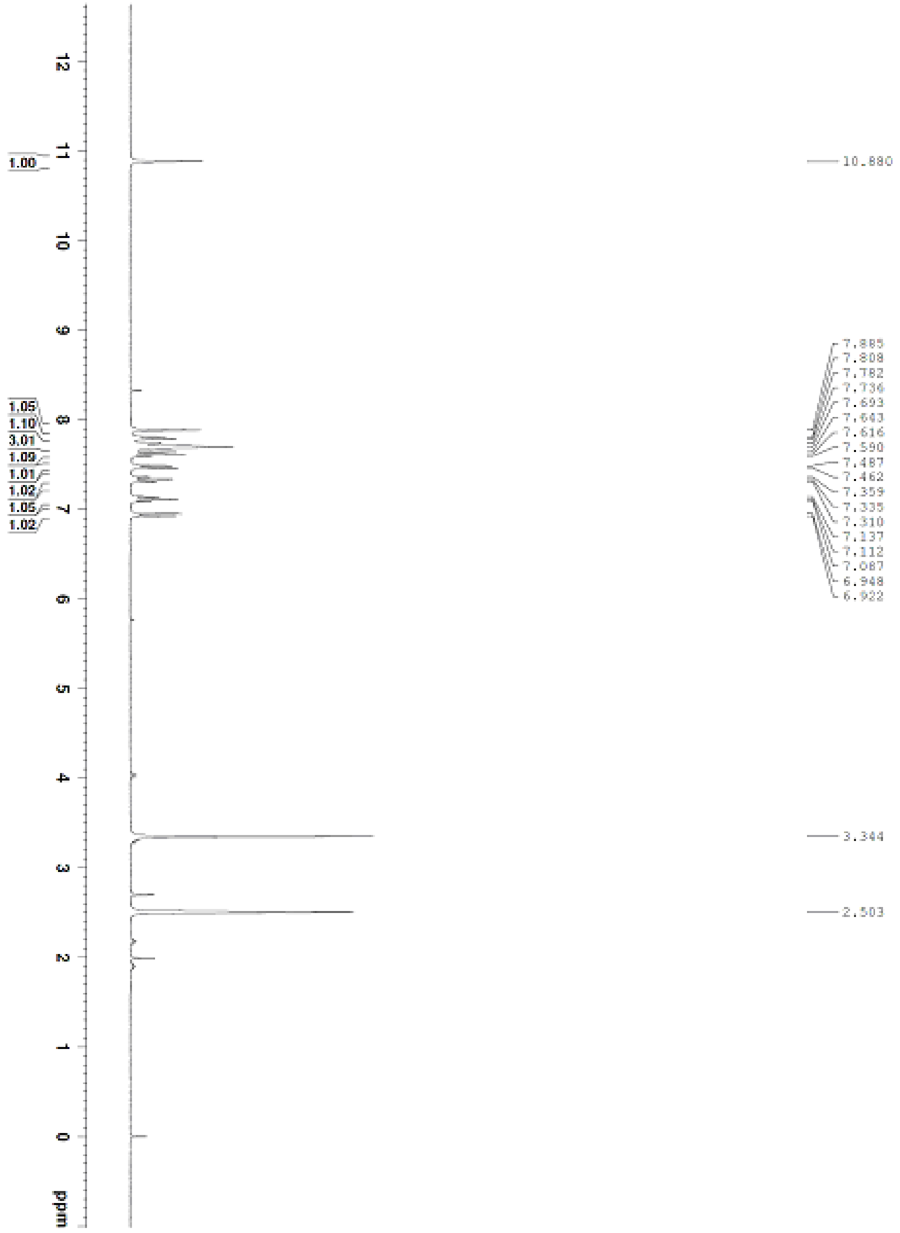

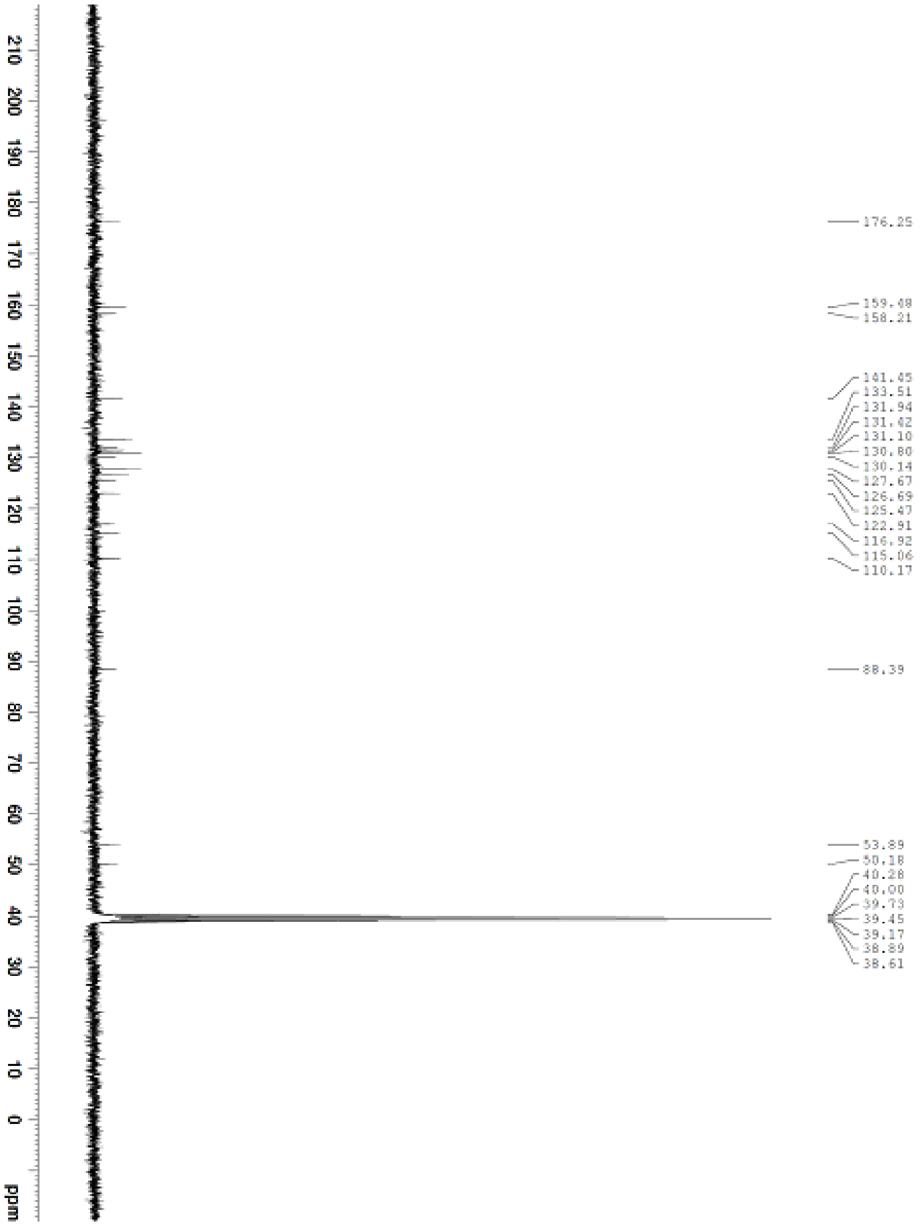
176.25
159.48
158.21
141.45
133.51
131.94
131.42
131.10
130.80
130.14
127.67
126.69
125.47
122.91
116.92
115.06
110.17
88.39
53.89
50.18
40.28
40.00
39.73
39.45
39.17
38.89
38.61
210
200
190
180
170
160
150
140
130
120
110
100
90
80
70
60
50
40
30
20
10
0
ppm

(*S*)-2'-amino-6'-(4-chlorophenyl)-2-oxospiro[indoline-3,4'-pyran]-3',5'-dicarbonitrile (**Q4r**)

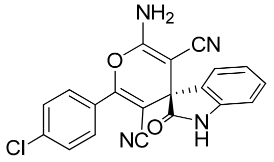

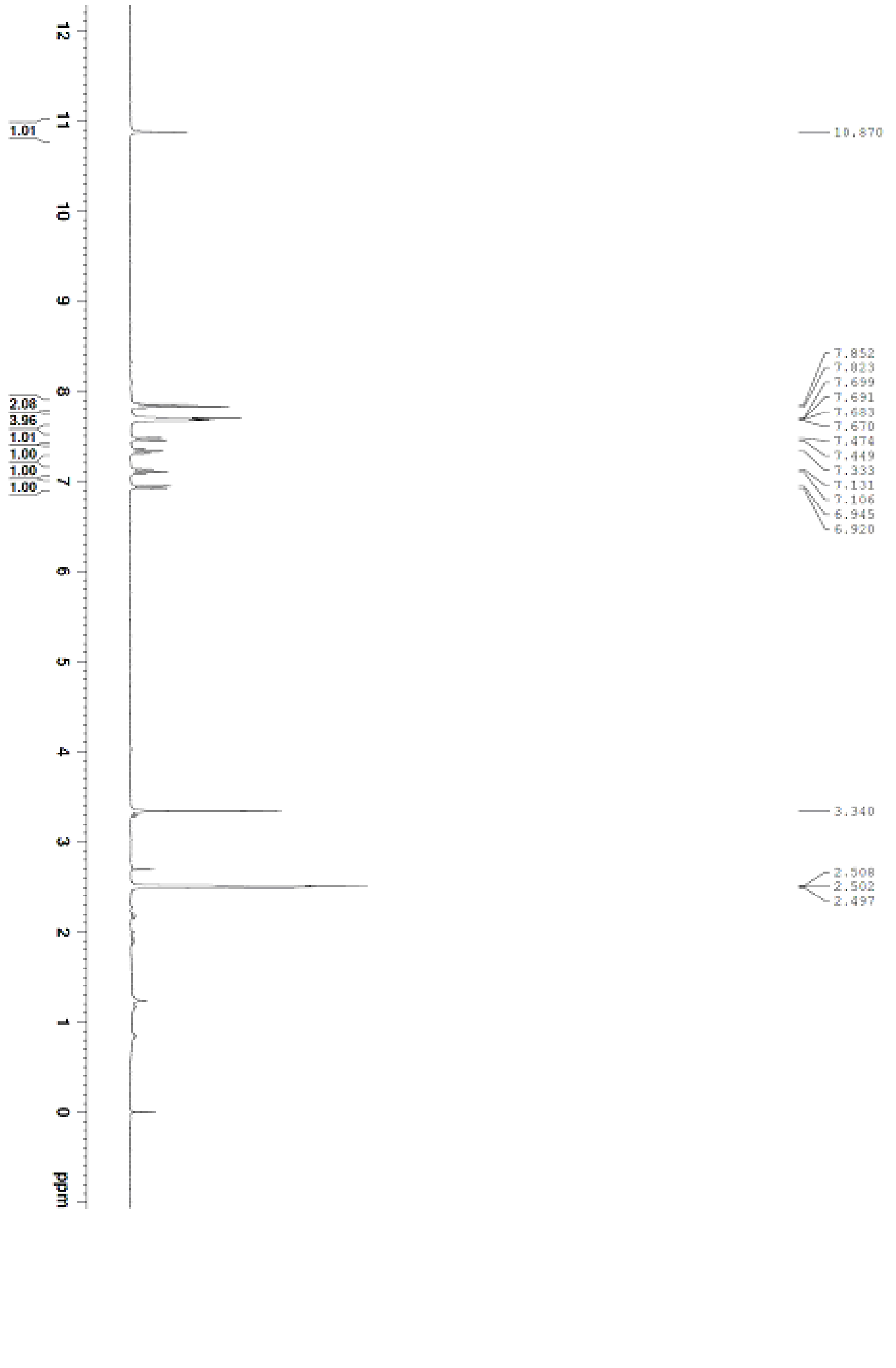

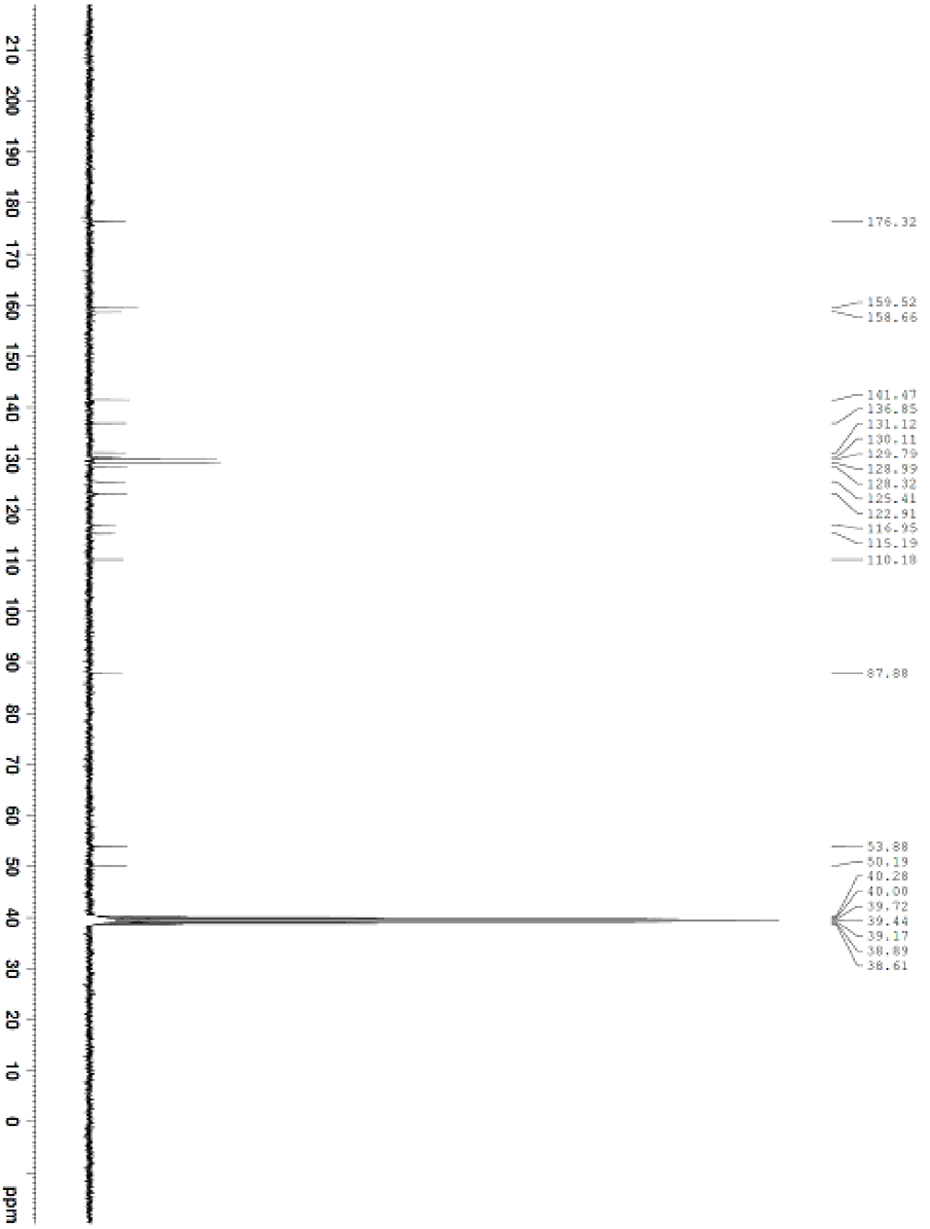
176.32
159.52
158.66
141.47
136.85
131.12
130.11
129.79
128.99
128.32
125.41
122.91
116.95
115.19
110.18
87.88
53.88
50.19
40.28
40.00
39.72
39.44
39.17
38.89
38.61
ppm

(*S*)-2'-amino-2-oxo-6'-m-tolylspiro[indoline-3,4'-pyran]-3',5'-dicarbonitrile (**Q4s**)

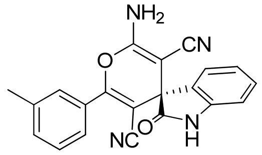

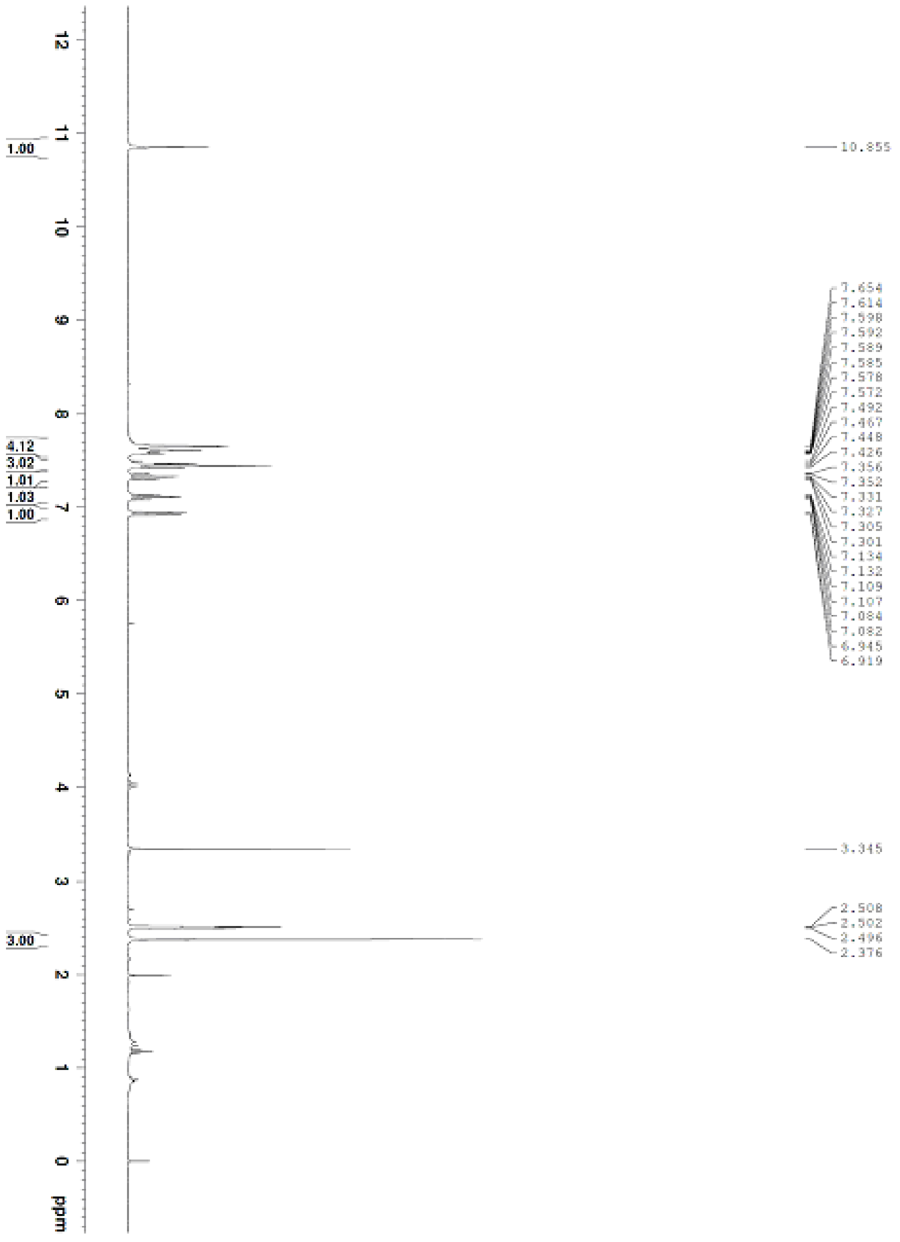

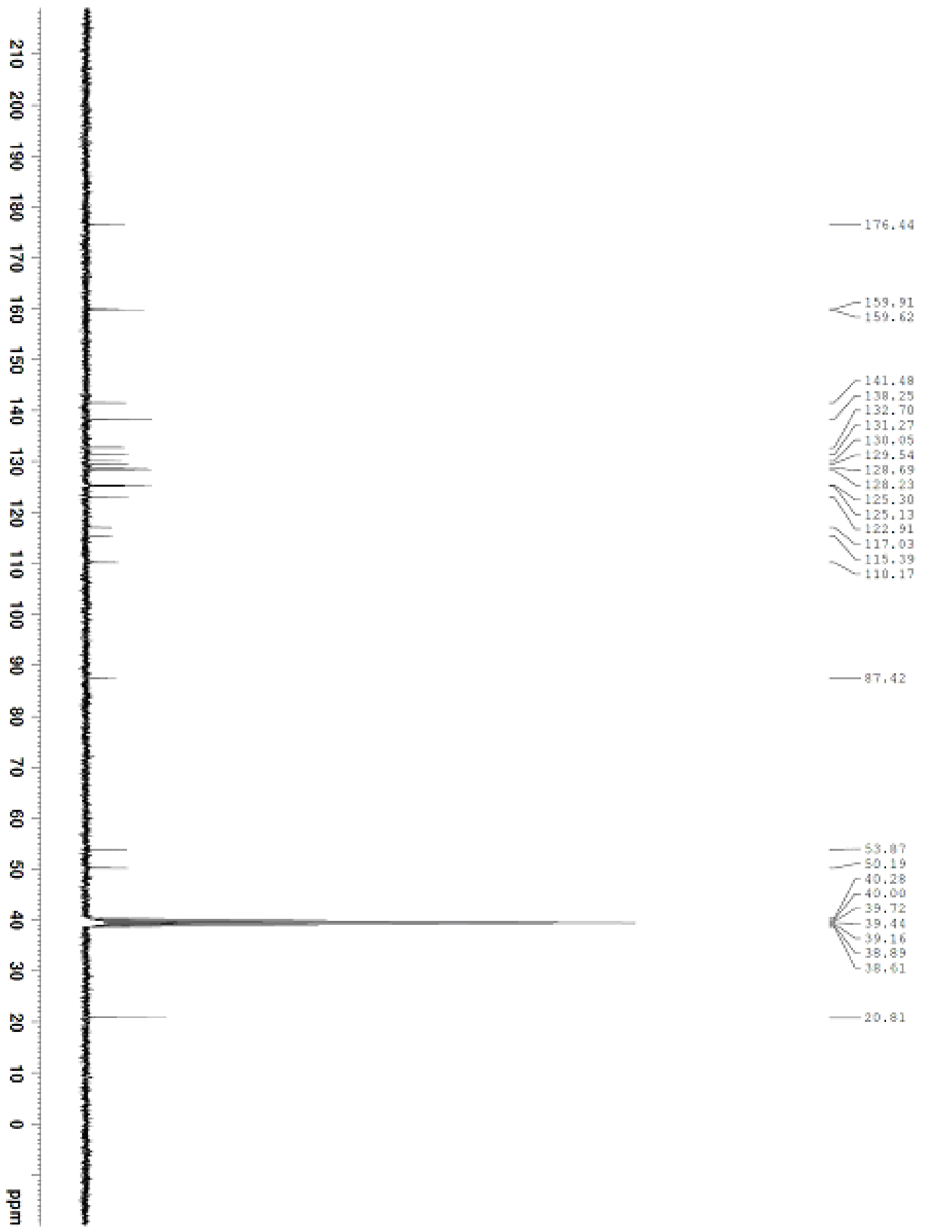
176.44
159.91
159.62
141.48
138.25
132.70
131.27
130.05
129.54
128.69
128.23
125.30
125.13
122.91
117.03
115.39
110.17
87.42
53.87
50.19
40.28
40.00
39.72
39.44
39.16
38.89
38.61
20.81
ppm

(*S*)-2'-amino-2-oxo-6'-p-tolylspiro[indoline-3,4'-pyran]-3',5'-dicarbonitrile (**Q4t**)

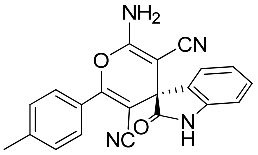

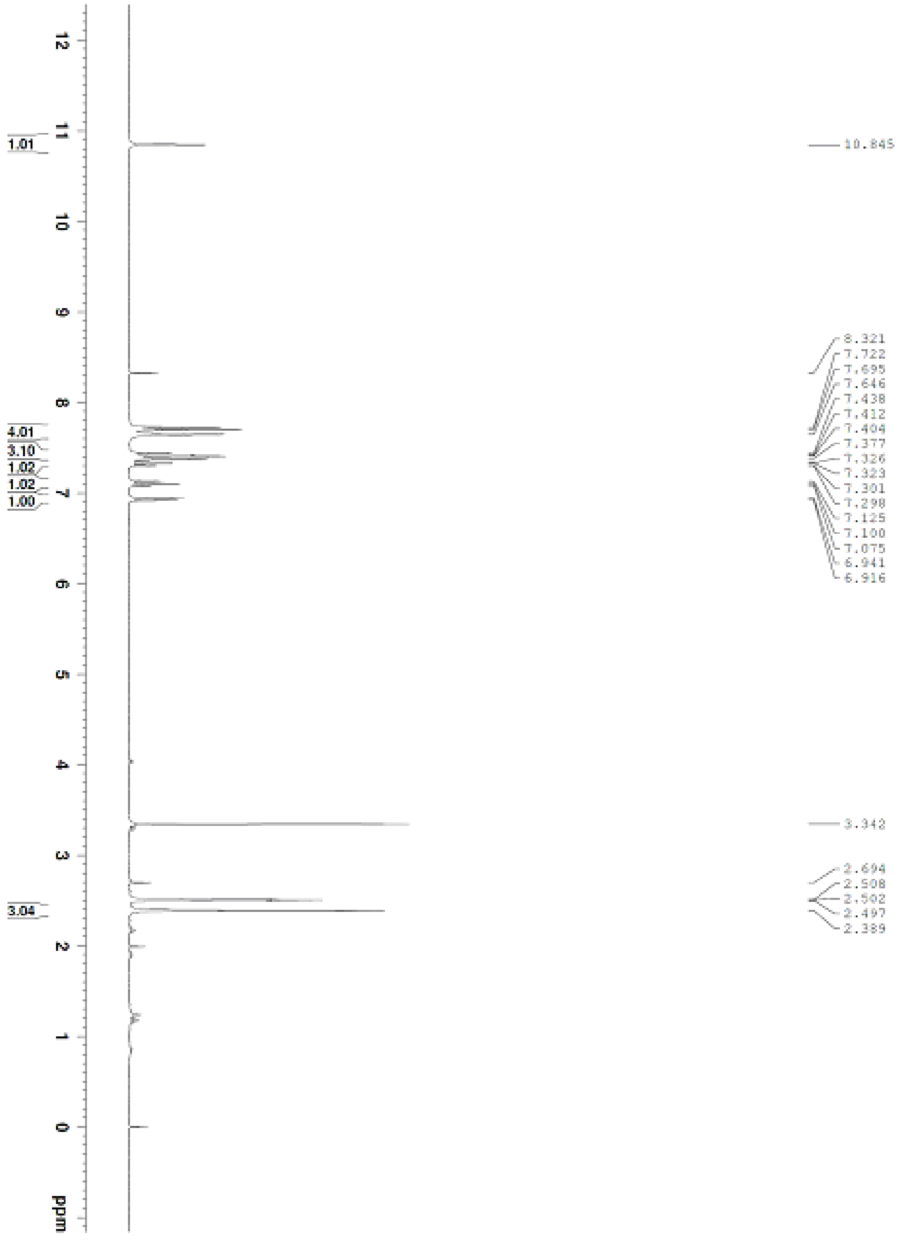

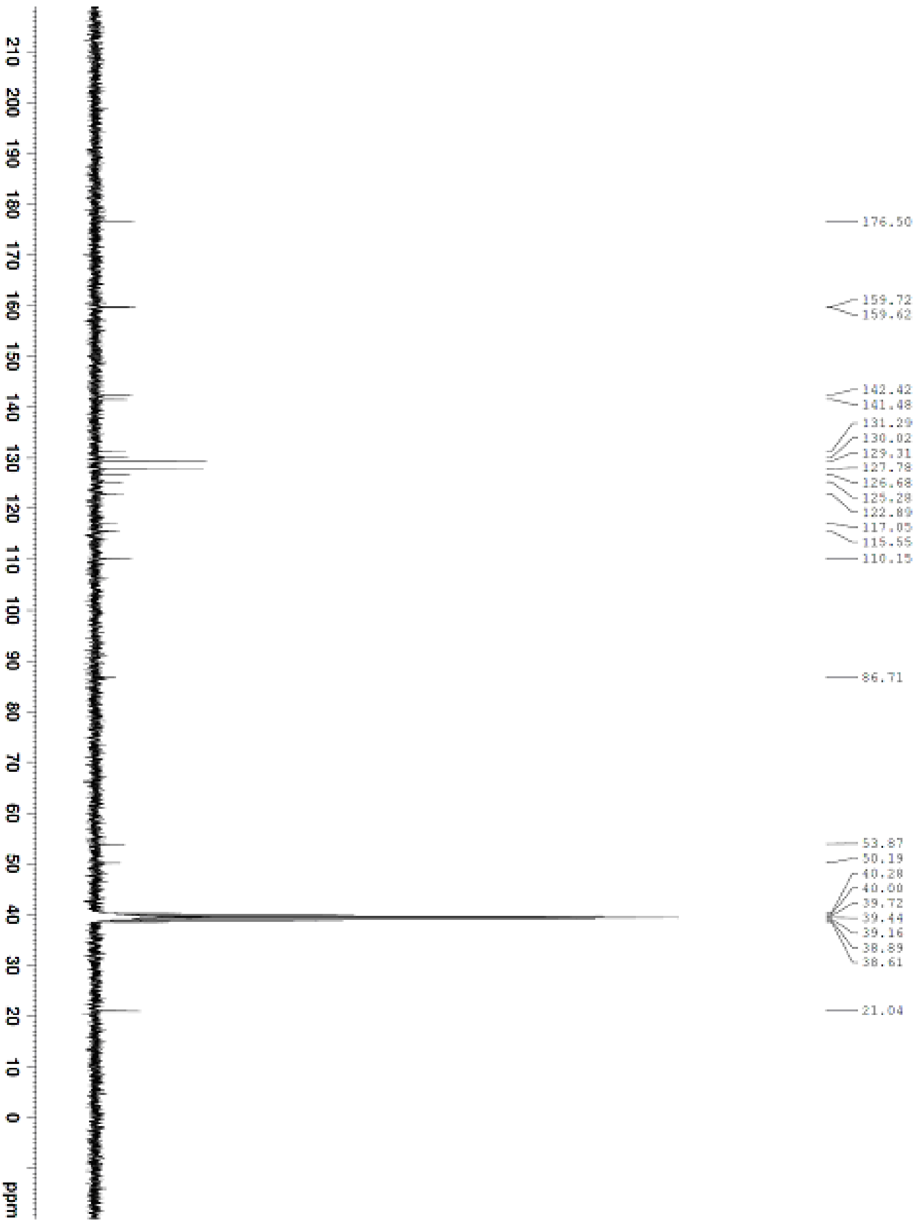
176.50
159.72
159.62
142.42
141.48
131.29
130.02
129.31
127.78
126.68
125.28
122.89
117.05
115.55
110.15
86.71
53.87
50.19
40.28
40.00
39.72
39.44
39.16
38.89
38.61
21.04
210 200 190 180 170 160 150 140 130 120 110 100 90 80 70 60 50 40 30 20 10 0 ppm
